# Ceftobiprole versus ceftriaxone ± linezolid in Community-Acquired Bacterial Pneumonia (CABP): Re-analysis of a randomized, phase 3 study using 2020 FDA guidance

**DOI:** 10.1371/journal.pone.0326758

**Published:** 2025-06-24

**Authors:** Andrew F. Shorr, Mark E. Jones, Silke Friedmann, Daniel Ionescu, Mikael Saulay, Jennifer I. Smart, Marc Engelhardt

**Affiliations:** 1 Pulmonary and Critical Care Medicine, Medstar Washington Hospital, Washington, Columbia, United States of America; 2 Basilea Pharmaceutica International Ltd, Allschwil, Switzerland; Aga Khan University, PAKISTAN

## Abstract

**Background:**

Ceftobiprole is an advanced-generation cephalosporin with activity against methicillin-resistant *Staphylococcus aureus*, resistant pneumococci, and Enterobacterales. In a Phase 3 study in community-acquired bacterial pneumonia (CABP) performed prior to the current US Food and Drug Administration (FDA) guidance, clinical cure at the test-of-cure visit (TOC) was the primary endpoint. We present a re-analysis using early clinical success as the primary endpoint per the 2020 FDA CABP Guidance.

**Methods:**

In this multicenter, double-blind study, patients with CABP requiring hospitalization were randomized to intravenous ceftobiprole (500 mg q8h) or ceftriaxone (2000 mg q24h) ± linezolid (600 mg q12h) for 3–14 days. The primary endpoint was clinical success at Day 3 (improvement in ≥2 symptoms of chest pain, cough, productive sputum, difficulty breathing). A 12.5% non-inferiority margin was used.

**Results:**

Of the original 638 patients, 618 (97%) had ≥ 2 symptoms and were included in the Day 3 Intent-to-Treat (ITT) population for the re-analysis. The Day 3 modified ITT population consisted of 187 patients (29%) meeting additional FDA Guidance including Patient Outcomes Research Team (PORT) classification ≥III. Ceftobiprole was non-inferior to ceftriaxone for clinical success for Day 3 ITT (71.0% vs 71.1%) and Day 3 modified ITT (71.1% vs 66.7%) populations. The 28-day all-cause mortality was 1.6% (ceftobiprole) vs 2.6% (ceftriaxone) in the Day 3 ITT population and 2.1% vs 7.8% in the Day 3 modified ITT population.

**Conclusion:**

Ceftobiprole was non-inferior to ceftriaxone ± linezolid for clinical success at Day 3 according to the current 2020 FDA CABP Guidance.

## Introduction

Community-acquired bacterial pneumonia (CABP) represents a major cause of hospitalization and mortality. CABP is defined as an acute infection of the lung parenchyma accompanied by a new infiltrate on chest radiography with the infection being acquired outside of the hospital. Frequently-involved bacterial pathogens include *Streptococcus pneumoniae*, *Haemophilus influenzae*, *Staphylococcus aureus*, and *Enterobacteriaceae*. [1,2]. Among adults under the age of 65 years in the United States, the incidence of CABP ranges from 24.8/10,000 person-years [3] and 106/10,000 person-years [4]. Among the elderly both the prevalence and burden of CABP are higher. For example, the incidence of CABP in the elderly is approximately 63.0/10,000 person-years in 65–79-year-olds and climbs to 164.3/10,000 person-years for those 80 years of age and older [1]. Furthermore, in the US, CABP results in nearly 1.5 million hospitalizations annually. With respect to mortality, CABP remains a leading cause of death with a 30-day mortality rate of 13% among those requiring hospitalization. In Europe, CABP results in a similar burden to patients and to the healthcare system. The economic burden of CABP is excessive due to its frequency and the fact that it often leads to hospitalization, especially among the elderly and those with multiple comorbidities. Prompt administration of antibiotics which are *in vitro* active against the culprit pathogen is a key determinant of outcomes in hospitalized CABP. Initially-appropriate therapy in CABP reduces not only mortality but also overall costs.

Ceftobiprole, the active moiety of its prodrug ceftobiprole medocaril, is an advanced-generation cephalosporin. This unique agent binds tightly to penicillin-binding proteins (PBPs), including PBPs causing β-lactam resistance in staphylococci (PBP2a) and pneumococci (PBP2x). In addition, the risk of drug resistance developing is low [5–7]. Ceftobiprole also has potent *in vitro* activity against Gram-positive pathogens, including methicillin-resistant *Staphylococcus aureus* (MRSA) and penicillin-resistant *Streptococcus pneumoniae*, and also against Gram-negative pathogens – many of which are often implicated as causes of CABP [8–10]. Ceftobiprole is not active against extended-spectrum β-lactamase (ESBL)- or carbapenemase-producing Gram-negative pathogens.

Ceftobiprole is approved in European countries for treating CABP and hospital-acquired bacterial pneumonia (HABP) excluding ventilator-associated bacterial pneumonia. This approval was based on the findings of Phase 3 studies conducted in patients with CABP and HABP [11,12]. A retrospective analysis of these CABP and HABP studies demonstrated that ceftobiprole resulted in early clinical improvement among severely-ill patients [13].

The original Phase 3 ceftobiprole CABP study was conducted from June 2006 to July 2007 [11] (NCT00326287), before the current Food and Drug Administration (FDA) CABP Guidance for Industry was issued [14]. In that guidance, the FDA specified that the primary endpoint for clinical trials in CABP was to be clinical success at Day 4 (as opposed to investigator-assessed clinical success). Furthermore, the FDA provided a novel definition for measuring this early clinical response. The FDA also modified the entry criteria for clinical trials by eliminating the eligibility of patients with less severe infections. We sought to explore outcomes from the initial ceftobiprole Phase 3 CABP randomized trial based on these revised FDA criteria. Specifically, we aimed to determine retrospectively the efficacy and safety of ceftobiprole for CABP given the current, more stringent FDA criteria.

## Methods

The study design and methods for the original study have been previously published [11]. We describe below the methods employed for the re-analysis based on 2020 FDA CABP Guidance. A detailed comparison of study entry criteria for the original analysis and the present re-analysis is provided in Table S1 in S1 File. An Institutional Review Board or Ethics Committee at each site approved the protocol and informed consent form before study initiation (ClinicalTrials.gov identifier: NCT00326287; http://www.clinicaltrials.gov). As this was a re-analysis of the original study data, ethical oversight was not required for this post-hoc analysis. All patients provided written consent for participation in the study, which was conducted in accordance with the Declaration of Helsinki. Basilea Pharmaceutica International Ltd, Allschwil Switzerland is the owner of the data, which were accessed on 5-April-2023. All data were coded and none of the authors or the Sponsor (Basilea Pharmaceutica International Ltd, Allschwil, Switzerland) had access to information that could identify individual participants.

### Patient Selection

In accordance with the 2020 FDA CABP guidance [14], we restricted the present analysis to patients with at least two of the following symptoms at baseline: difficulty breathing (based on symptoms of dyspnea or tachypnea reported in the study), cough, production of purulent sputum or chest pain (based on symptom of pleuritic chest pain reported in the study). The majority of patients in the original Intent-to-Treat (ITT) population (96.9%, 618/638) satisfied these criteria. Thus, for the purposes of the re-analysis, this group of 618 patients was defined as the ‘Day 3 ITT population’. The ‘Day 3 ITT population’ was used as the ITT population for the re-analyses of primary and secondary efficacy endpoints. In addition, we created a second ‘Day 3 modified ITT population’ for the re-analyses of primary and secondary efficacy endpoints as stipulated in the 2020 FDA CABP Guidance [14]. This population included 187 patients (97 treated with ceftobiprole and 90 with the comparator) who met all of the following criteria:

- A baseline classification of Pneumonia Outcomes Research Team (PORT) Risk Class ≥III [15]
- At least two of either tachypnea (surrogate for “difficulty breathing”), cough, production of purulent sputum or pleuritic chest pain
- At least two of fever, hypothermia, hypotension, tachycardia or tachypnea
- At least one of new-onset hypoxemia, rales or pulmonary consolidation, or leukocytosis or leukopenia
- A new radiographic infiltrate (not related to another disease process) consistent with the diagnosis of bacterial pneumonia

### Re-analysis endpoints

For the current re-analyses, efficacy on Day 3 (in accord with the FDA early endpoint) served as the primary endpoint. Efficacy was defined as clinical success at an early time point [14] based on improvement in important clinical symptoms (see below). In the original study multiple symptoms were recorded and subsequently mapped to correspond with the current key symptoms noted in the 2020 FDA CABP Guidance. Specifically, we examined: 1) pleuritic chest pain, 2) cough, 3) purulent sputum production or respiratory secretion, and 4) tachypnea (note: tachypnea was assessed in the original analysis rather than difficulty breathing). In addition, rigor or shaking chills, hypoxemia, and rales and/or pulmonary consolidation were specifically assessed. Each symptom was scored at study Day 3 as 1) Present; 2) Absent; 3) Worsened; 4) Unchanged; or 5) Improved.

For the primary endpoint, clinical success was defined by at least a 1-point improvement from baseline to the Day 3 in the cardinal clinical symptoms noted above. Furthermore, clinical success required no worsening of other symptoms. Clinical success was analyzed at Day 3 because the study protocol specified that patients could be switched to oral cefuroxime after Day 3. A detailed comparison of definitions for the primary endpoint for the original study and the re-analysis is provided in Table S2 in S1 File.

We additionally noted rates of improvement at Day 3 in at least two of the following symptoms: chest pain, cough, amount of productive sputum or tachypnea; and improvement in vital signs (body temperature, blood pressure, heart rate, and respiratory rate). The following criteria at Day 3 were used for this assessment: 1) Body temperature ≤37.8°C; 2) Heart rate ≤100/min; 3) Respiratory rate ≤24 breaths/min; 4) Systolic blood pressure ≥90 mmHg. Patients were not classified as improved if there was worsening in any symptom. We noted general measures of safety and tolerability, as well,

Other endpoints were also assessed: 1) blinded investigator-assessed clinical outcome, i.e., improvement/resolution of symptoms of CABP at the end of 2) clinical cure at the test-of-cure (TOC) visit, which was the primary endpoint in the original analysis; and all-cause mortality at Day 28.

### Statistical analysis

As with the initial trial, we focused on assessing non-inferiority between ceftobiprole and the comparator (ceftriaxone + /- linezolid). A two-sided 95% confidence interval (CI) using the normal approximation to the difference of two binomial proportions, was calculated for the between-treatment difference (ceftobiprole minus ceftriaxone ± linezolid) at the TOC visit. The result for the primary endpoint of the re-analysis was interpreted in the context of a 12.5% non-inferiority margin, i.e., non-inferiority for ceftobiprole compared with ceftriaxone ± linezolid was considered to be met if the lower limit of this CI was greater than or equal to −12.5%. We examined outcomes in select subgroups of interest. Specifically, the same subgroups explored in the initial trial were analyzed in the present assessment. For all secondary and other analyses included in the re-analysis, a two-sided 95% CI using the normal approximation to the difference of two binomial proportions was calculated for the between-treatment difference (ceftobiprole minus ceftriaxone ± linezolid). Analyses of the secondary and other endpoints were descriptive with no testing against an interpretative non-inferiority margin.

The study was not prospectively powered to perform non-inferiority testing for the re-analysis of the primary endpoint in accordance with the 2020 FDA CABP Guidance [14]. Retrospectively, we calculated that a sample size of 300 patients per group provided >80% power using a point estimate for clinical success at Day 3 of 50% in each treatment group in the ITT population at a one-sided alpha level of 0.025 and a non-inferiority margin of 12.5% for the between- group difference in the primary endpoint. Using a point estimate for clinical success at Day 3 of 70% in each treatment group in the ITT population provided >90% power.

## Results

**Fig 1:**
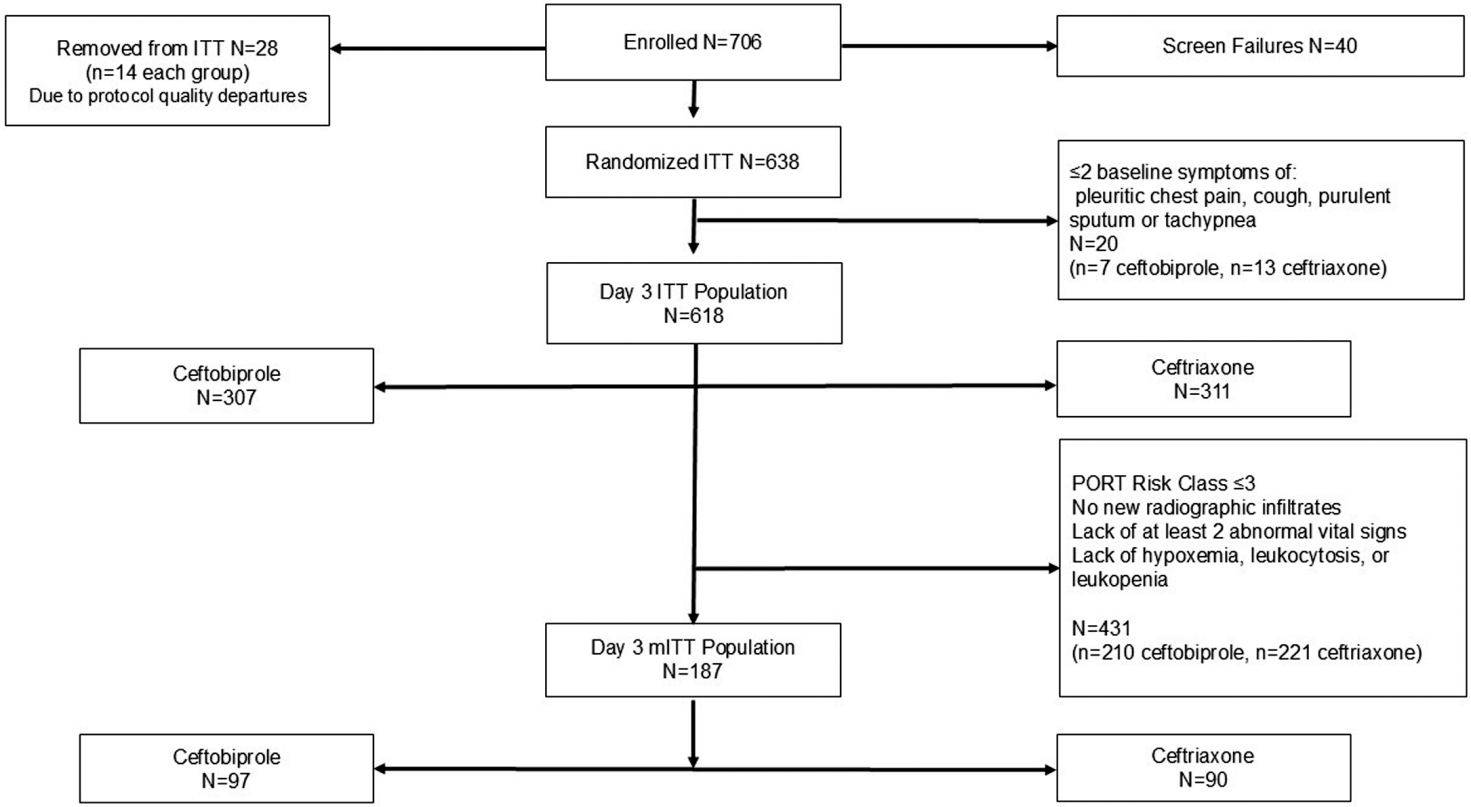
CONSORT flowchart.

Patients enrolment for this study started on 5 June 2006 and the last patient completed the study on 19 July 2007. Patient disposition is shown in Fig 1. Baseline demographic and clinical characteristics for the ceftobiprole and ceftriaxone groups were generally comparable in the reanalysis (Table 1). Some differences were apparent between ITT and modified ITT groups, notably higher median age (56 vs. 67 years) and higher proportions of white and Hispanic patients. Furthermore, by design given the FDA 2020 CABP Guidance, the modified ITT population was notably more severely ill as demonstrated by the distribution of PORT scores ≥IV (22.3% vs. 49.7%) and the prevalence of the systemic inflammatory response syndrome (SIRS) (53.7% vs. 65.2%) in the modified ITT group. Additionally, in the modified ITT group, SIRS was noted more often in subjects randomized to ceftobiprole (72.2% vs 57.8%).

**Table 1:** Baseline characteristics for prespecified Day 3 ITT population and Day 3 modified ITT population.

	Day 3 ITT population			Day 3-modified ITT population		
**Ceftobiprole** **(N = 307)**	**Ceftriaxone** ^*^ **(N = 311)**	**Total** **(N = 618)**	**Ceftobiprole** **(N = 97)**	**Ceftriaxone** ^*^ **(N = 90)**	**Total** **(N = 187)**
Median age (years), (range)	56.0 (18–90)	55.0 (18–94)	56.0 (18–94)	65.0 (21–86)	71.0 (24–89)	67.0 (21–89)
Gender, n (%)						
Female	133 (43.3)	132 (42.4)	265 (42.9)	37 (38.1)	32 (35.6)	69 (36.9)
Male	174 (56.7)	179 (57.6)	353 (57.1)	60 (61.9)	58 (64.4)	118 (63.1)
Race, n (%)						
White	189 (61.6)	194 (62.4)	383 (62.0)	69 (71.1)	65 (72.2)	134 (71.7)
Black.	18 (5.9)	17 (5.5)	35 (5.7)	2 (2.1)	3 (3.3)	5 (2.7)
Asian	66 (21.5)	70 (22.5)	136 (22.0)	11 (11.3)	12 (13.3)	23 (12.3)
Other^a^	34 (11.1)	30 (9.6)	64 (10.4)	15 (15.5)	10 (11.1)	25 (13.4)
Ethnicity, n (%)						
Hispanic or Latino ethnicity	72 (23.5)	81 (26.0)	153 (24.8)	33 (34.0)	32 (35.6)	65 (34.8)
Not Hispanic or Latino	235 (76.5)	228 (73.3)	463 (74.9)	64 (66.0)	57 (63.3)	121 (64.7)
Geographic regions, n (%)						
USA	40 (13.0)	39 (12.5)	79 (12.8)	7 (7.2)	10 (11.2)	17 (9.1)
Europe^b^	129 (42.0)	126 (40.5)	255 (41.3)	43 (44.3)	36 (40.0)	79 (42.2)
Other^c^	138 (45.0)	146 (46.9)	284 (46.0)	47 (48.5)	44 (48.9)	91 (48.7)
Patients with any valid baseline pathogen, n (%)	85 (27.7)	96 (30.9)	181 (29.3)	32 (33.0)	27 (30.0)	59 (31.6)
Gram-positive pathogen	46 (15.0)	52 (16.7)	98 (15.9)	18 (18.6)	14 (15.6)	32 (17.1)
Gram-negative pathogen	49 (16.0)	51 (16.4)	100 (16.2)	19 (19.6)	14 (15.6)	33 (17.6)
Monomicrobial	69 (22.5)	88 (28.3)	157 (25.4)	25 (25.8)	26 (28.9)	51 (27.3)
Polymicrobial	16 (5.2)	8 (2.6)	24 (3.9)	7 (7.2)	1 (1.1)	8 (4.3)
PORT classification^d^, n (%)						
≤ III	238 (77.5)	241 (77.5)	479 (77.5)	53 (54.6)	41 (45.6)	94 (50.3)
≥ IV	69 (22.5)	69 (22.2)	138 (22.3)	44 (45.4)	49 (54.4)	93 (49.7)
PORT classification, n (%)						
I	18 (5.9)	14 (4.5)	32 (5.2)	0 (0.0)	0 (0.0)	0 (0.0)
II	133 (43.3)	152 (48.9)	285 (46.1)	0 (0.0)	0 (0.0)	0 (0.0)
III	87 (28.3)	75 (24.1)	162 (26.2)	53 (54.6)	41 (45.6)	94 (50.3)
IV	65 (21.2)	62 (19.9)	127 (20.6)	41 (42.3)	43 (47.8)	84 (44.9)
V	4 (1.3)	7 (2.3)	11 (1.8)	3 (3.1)	6 (6.7)	9 (4.8)
Missing	0	1 (0.3)	1 (0.2)	0 (0.0)	0 (0.0)	0 (0.0)
Received linezolid/placebo (%)^e^	26 (8.5)	36 (11.6)	62 (10.0)	7 (7.2)	13 (14.4)	20 (10.7)
From baseline	26 (8.5)	31 (10.0)	57 (9.2)	7 (7.2)	11 (12.2)	18 (9.6)
Started after baseline	0 (0.0)	5 (1.6)	5 (0.8)	0 (0.0)	2 (2.2)	2 (1.1)
Had SIRS n (%)	162 (52.8)	170 (54.7)	332 (53.7)	70 (72.2)	52 (57.8)	122 (65.2)
Prior antibiotic therapy n (%)^f^	180 (58.6)	190 (61.1)	370 (59.9)	57 (58.8)	58 (64.4)	115 (61.5)

ITT = Intent-to-Treat; NA: not applicable; PORT = Pneumonia Outcomes Research Team; PSI = Pneumonia Severity Index; SIRS = systemic inflammatory response syndrome.

* With or without linezolid.

^a^ Includes Hispanic and mixed race patients.

^b^ Czech Republic, Germany, Hungary, Lithuania, Poland, Russia, Ukraine.

^c^ Argentina, Brazil, Costa Rica, Hong Kong, Mexico, Panama, People’s Republic of China, Republic of Korea, Taiwan.

^d^ PSI score ≤ 90 corresponds to PORT classifications I, II, III; PSI score ≥ 91 corresponds to PORT classifications IV, V.

^e^ Up to and including Day 3.

^f^ Refers to any prior antibiotic used within 30 days prior to first dose.

### Efficacy analyses

As Table 2 shows, outcomes were similar between patients treated with ceftobiprole and those randomized to the comparator arm. For the primary endpoint, the re-analysis of clinical success at Day 3 in the ITT and modified ITT populations showed that the lower bound of the 95% confidence interval of the difference between ceftobiprole and ceftriaxone treatment groups showed non-inferiority of ceftobiprole versus ceftriaxone considering a 12.5% non-inferiority margin (Table 2). All-cause mortality at Day 28 was generally low, and the rates in the modified ITT population were 2.1% for ceftobiprole and 7.8% for ceftriaxone.

**Table 2:** Efficacy analyses from re-analysis for Day 3 ITT population and modified ITT population.

	Ceftobiprole n/N (%)	Ceftriaxone^*^ N (%)	Difference^†^ (95% CI)
**Efficacy analyses (Day 3-ITT population)**			
Clinical success at Day 3	218/307 (71.0)	221/311 (71.1)	−0.1% (−7.2, 7.1)
28-day all-cause mortality	5/307 (1.6)	8/311 (2.6)	−0.9% (−3.2, 1.3)
Clinical success at Day 3 and improvement in vital signs	178/307 (58.0)	177/311 (56.9)	1.1% (−6.7, 8.9)
Clinical cure or improvement at EOT^‡^	255/307 (83.1)	264/311 (84.9)	−1.8% (−7.6, 4.0)
Clinical cure at TOC	235/307 (76.5)	249/311 (80.1)	−3.5 (−10.0, 3.0)
**Efficacy analyses (Day 3-modified ITT population)**			
Clinical success at Day 3	69/97 (71.1)	60/90 (66.7)	4.5% (−8.8; 17.7)
28-day all-cause mortality	2/97 (2.1)	7/90 (7.8)	−5.7% (−11.9; 0.5)
Clinical success at Day 3 and improvement in vital signs	45/97 (46.4)	47/90 (52.2)	−5.8% (−20.1; 8.5)
Clinical cure or improvement at EOT^‡^	83/97 (85.6)	74/90 (82.2)	3.3% (−7.2; 13.9)
Clinical cure at TOC	77/97 (79.4)	70/90 (77.8)	1.6 (−10.2, 13.4)

n = number of patients with respective outcome; N = total number of patients in the respective category.

CE = clinically evaluable; CI = confidence interval; EOT = End-of-treatment; ITT = Intent-to-Treat.

* Ceftriaxone with or without linezolid.

† Difference of ceftobiprole minus ceftriaxone with or without linezolid. The two-sided 95% CI was based on the Normal approximation to the difference of the two proportions.

‡ Based on investigator assessment.

The result for the primary endpoint of the re-analysis was interpreted in the context of a 12.5 non-inferiority margin, i.e., non-inferiority of ceftobiprole compared with ceftriaxone with or without linezolid was interpreted if the lower limit of this confidence interval was greater than or equal to −12.5%.

**Fig 2:**
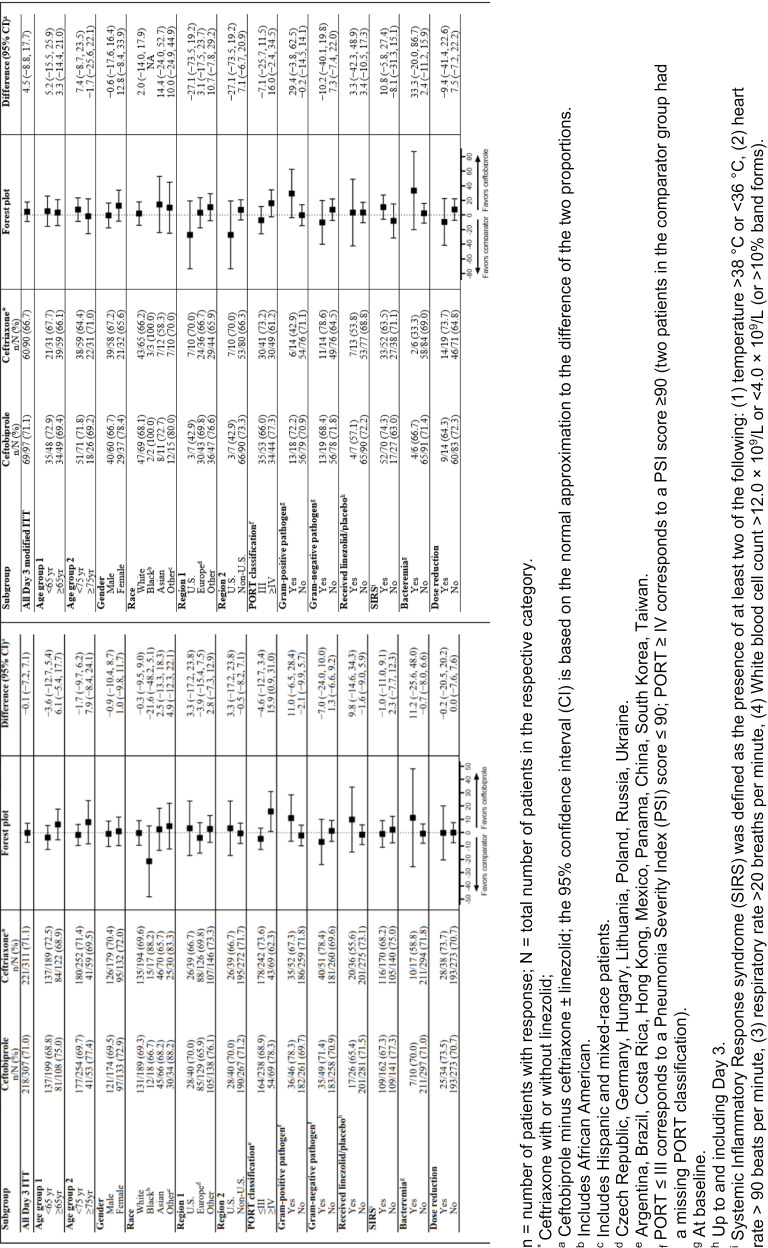
Subgroup analyses of the re-analysis primary endpoint for Day 3 ITT population and modified ITT population.

Subgroup results from the re-analysis of clinical success at Day 3 in the ITT and modified ITT populations revealed that most comparisons resulted in similar point estimates for ceftobiprole and ceftriaxone (Fig 2) However, in the ITT population, differences between ceftobiprole and ceftriaxone exceeding the 12.5% limit were noted for those classified as black 66.7% vs. 88.2% (mean difference −21.6%, 95% CI: −48.2, 5.1) and in the cohort of PORT score ≥IV (78.1% vs. 62.3% (mean difference: 15.9%, 95% CI: 0.9, 31.0)). The wide confidence intervals around these point estimates reflect the relatively small sample sizes in these select subgroups. For the modified ITT population, subgroup analyses demonstrated no meaningful differences between overall point estimates of efficacy and outcomes in the various subpopulations. More importantly, in subgroups of more severely ill patients based on either higher PORT scores, the presence of SIRS or isolation of a bloodstream infection, cure rates were similar between ceftobiprole and ceftriaxone treated patients.

**Fig 3:**
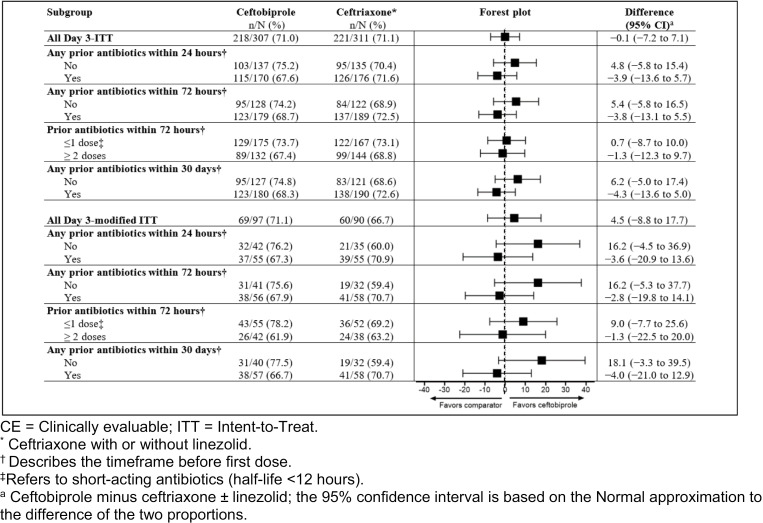
Re-analysis of primary endpoint by prior antibiotic use.

Re-analysis of clinical success at Day 3 stratified by prior antibiotic use indicated consistent efficacy results between ceftobiprole and ceftriaxone treatment groups for both the ITT and modified ITT populations (Fig 3). Clinical success at Day 3 was assessed by causative pathogen (Table 3). All pathogens had a minimum inhibitory concentration (MIC) ≤2 mg/L in both the microbiological ITT and the modified microbiological ITT populations (see **Table S3 in** S1 File). Among patients with positive blood cultures at baseline, clinical success at Day 3 in the microbiological ITT was 75.0% (6/8) with ceftobiprole and 60% (9/15) for ceftriaxone for Gram-positive pathogens, and in the modified microbiological ITT population was 80% (4/5) with ceftobiprole and 25% (1/4) with ceftriaxone (see Table S4 in S1 File).

**Table 3:** Clinical success at Day 3 for microbiological ITT population and modified microbiological ITT population by causative pathogen.

	Clinical success at Day 3 (Day 3-mITT)		Clinical success at Day 3 (Day 3-modified mITT)	
**Pathogen**	**Ceftobiprole** **(N = 85)** **n/N (%)**	**Ceftriaxone** ^*^ **(N = 96)** **n/N (%)**	**Ceftobiprole** **(N = 32)** **n/N (%)**	**Ceftriaxone** ^*^ **(N = 27)** **n/N (%)**
**Any Gram-positive**	**36/46 (78.3)**	**35/52 (67.3)**	**13/18 (72.2)**	**6/14 (42.9)**
*Streptococcus pneumoniae*	27/33 (81.8)	27/41 (65.9)	11/14 (78.6)	4/9 (44.4)
*Staphylococcus aureus*	10/14 (71.4)	7/10 (70.0)	3/4 (75.0)	1/4 (25.0)
MRSA	2/2 (100.0)	0/0 (NA)	0/0 (NA)	0/0 (NA)
MSSA	8/12 (66.7)	7/10 (70.0)	3/4 (75.0)	1/4 (25.0)
**Any Gram-negative**	**35/49 (71.4)**	**40/51 (78.4)**	**13/19 (68.4)**	**11/14 (78.6)**
Enterobacterales	14/18 (77.8)	13/16 (81.3)	5/7 (71.4)	6/7 (85.7)
* Enterobacter cloacae*	0/1 (0.0)	3/3 (100.0)	0/1 (0.0)	2/2 (100.0)
* Escherichia coli*	6/6 (100.0)	2/4 (50.0)	2/2 (100.0)	1/2 (50.0)
* Klebsiella pneumoniae*	7/8 (87.5)	7/8 (87.5)	2/3 (66.7)	2/2 (100.0)
*Haemophilus influenzae*	4/10 (40.0)	11/16 (68.8)	2/3 (66.7)	3/5 (60.0)
*Haemophilus parainfluenzae*	8/9 (88.9)	8/8 (100.0)	2/2 (100.0)	1/1 (100.0)
*Moraxella catarrhalis*	2/4 (50.0)	4/5 (80.0)	1/3 (33.3)	1/1 (100.0)
*Pseudomonas aeruginosa*	2/2 (100.0)	3/4 (75.0)	1/1 (100.0)	0/0 (NA)
*Acinetobacter baumannii*	2/3 (66.7)	1/1 (100.0)	1/2 (50.0)	0/0 (NA)

n = number of patients with a response; N = total number of patients in the respective category.

mITT = microbiological Intent-to-Treat; MRSA = methicillin-resistant *Staphylococcus aureus*; MSSA = methicillin-susceptible *Staphylococcus aureus.*

* Ceftriaxone with or without linezolid.

Additional new analyses were performed in order to provide information supportive to the outcomes reported from the re-analysis (see Tables S5, S6, S7, S8 and S9 in S1 File). These included primary endpoint by prior antibiotic use, microbiological eradication at the TOC visit by causative pathogen, clinical cure at TOC by baseline blood culture, clinical relapse, and clinical cure or microbiological eradication not sustained at the last follow-up visit. No differences between treatments were observed for prior antibiotic use, causative pathogen, baseline blood culture, clinical relapse or lack of sustained effect.

### Safety and tolerability

The Day 3 ITT population constituted 98% (618/632) of the original safety population, and safety and tolerability results were consistent with results published previously (Nicholson 2012). In the Day 3 modified ITT population, at least one adverse event (AE) occurred in 68.0% (66/97) with ceftobiprole versus 78.9% (71/90) comparator. Drug-related AEs were reported in 37.1% (36/97) with ceftobiprole and 33.3% (30/90) with comparator. The most frequent drug-related AEs with ceftobiprole were diarrhea 6.2%, laboratory test abnormalities (5.2%), nausea and phlebitis 4.1% each, alanine aminotransferase increased, headache and vomiting in 3.1% each. With comparator, most common were diarrhea (6.7%), blood triglycerides increased (4.4%), and alanine aminotransferase increased and hyponatremia (3.3% each). Serious AEs were reported in 14.4% (14/97) with ceftobiprole versus 20.0% (18/90) with comparator. Three patients experienced drug-related serious AEs, all in the comparator group, including *Clostridioides difficile* colitis, pneumonia, and respiratory failure. Adverse events leading to treatment discontinuation were reported in 5.2% (5/97) with ceftobiprole versus 6.7% (6/90) with comparator, including drug-related AEs in two patients with ceftobiprole (diarrhea, nausea, and vomiting in one patient and transaminase increased in one patient) and in three patients with comparator (hyperemia, pneumonia, and respiratory failure).

## Discussion

CABP remains a major challenge and most concur that clinicians require newer options for treating this serious infection. This re-analysis of a previously-completed Phase 3 trial of ceftobiprole for CABP shows that ceftobiprole appears to result in non-inferior clinical outcomes relative to ceftriaxone. As noted earlier, the FDA has updated its guidance for clinical trials for regulatory approval of novel agents for bacterial infectious diseases to focus on early outcomes. Hence, in order to explore the data from the original trial so as to place it in the context of other more recent randomized controlled trials in CABP, we re-examined the initial trial findings with a focus on both more stringent trial enrollment criteria and unique clinical endpoints. In this smaller, yet more severely ill population, we confirm the non-inferiority of ceftobiprole vs. ceftriaxone. We further demonstrate consistent results with ceftobiprole across several clinically relevant subgroups. Furthermore, no new safety or tolerability concerns were observed.

Importantly, all causative baseline pathogens had a MIC of 2 mg/L or less for ceftobiprole (see Table S3 in S1 File). In a population pharmacokinetic model using data from a study of patients with HABP [12], ceftobiprole plasma concentrations at the doses used in this study were expected to be effective in 92% of all patients against causative baseline pathogens with a ceftobiprole MIC of 4 mg/L [16]. Intriguingly, in the modified ITT population that most closely matched the patient selection criteria stipulated in the 2020 FDA CABP Guidance [14] and which included more severely ill patients (e.g., PORT Risk Class ≥III), the all-cause mortality at Day 28 was numerically lower in the ceftobiprole group. Of course, this observation can only be viewed as hypothesis generating.

The high rate of pathogen recovery in this study helps to ensure that patients with true CABP were enrolled. The high rate of pathogen isolation also makes the present analysis unique given that, historically, fewer than 50% of patients in epidemiologic analyses of CAP have a bacterial pathogen identified. As such, this differentiation represents a strength of the current analysis. In addition, despite restricting this analysis to a sicker population, we detected no new safety concerns. This is important as more severely ill patients with CABP generally have less reserve for tolerating AEs. The fact that ceftobiprole was well tolerated should reassure clinicians about the safety of ceftobiprole.

Our findings stand in contrast to other recent CABP trials. For example, omadacycline demonstrated early success in 85% patients with CABP, but only 26% of patients fell into PORT class IV class and none were in PORT class V [18]; in the original ceftobiprole study, 22% of patients were PORT IV or V class [11]. One should note that the ceftobiprole study did not enroll patients with atypical pathogens compared with both recent trials with either omadacycline (36%) or lefamulin (28%) [11,17,18].

A central limitation of this re-analysis was its post-hoc, retrospective approach. As such, not all data from the original study could be included in the re-analysis based on 2020 FDA CABP Guidance [14]. However, almost all patients included in the original publication [11] were included in this re-analysis and the outcomes of primary interest were included. Other limitations include the generalizability of these results to patients with CABP given the constraints of a randomized, controlled study design and the lack of enrollment of patients with MRSA.

In summary, the results of this re-analysis of a Phase 3 study with ceftobiprole using the 2020 FDA CABP Guidance are consistent with findings reported from the original study [11] and in agreement with findings from other reports assessing early improvement with ceftobiprole for patients with pneumonia [13,19]. Ceftobiprole is currently approved for treating CABP and HABP (excluding ventilator-associated pneumonia) in European countries, and for CABP, ABSSSI, and *Staphylococcus aureus* bacteremia in the United States. Ceftobiprole represents the potential for an antibiotic with diverse in vitro activity that can be used as monotherapy across a wide range of patients hospitalized with serious infections.

**Key points**

Ceftobiprole was non-inferior to ceftriaxone ± linezolid for clinical success at Day 3 in patients with community-acquired bacterial pneumonia according to the current FDA CABP Guidance.

## Supporting information

S1 File **S1 Table.** Clinical, radiographic, and microbiologic entry criteria in the 2020 FDA CABP Guidance compared to inclusion criteria for study CAP-3001 (Nicholson et al, 2012). **S2 Table**. Comparison of the primary endpoint defined in the 2020 FDA CABP guidance with the equivalent pre-specified endpoint (Nicholson et al, 2012). **S3 Table.** Re-analysis: Clinical success at Day 3 by causative pathogen in accordance with the 2020 FDA Guidance and by MIC (ceftobiprole). **S4 Table.** Re-analysis: Clinical success at Day 3 by blood culture pathogen at baseline in accordance with the 2020 FDA Guidance. **S5 Table.** CAP-3001: Analyses of the pre-specified primary study endpoint by prior antibiotic use. **S6 Table.** CAP-3001: Microbiological eradication at the TOC visit by causative pathogen (mITT and ME populations). **S7 Table**. CAP-3001: Clinical cure at the TOC visit by blood culture pathogens at baseline (pre-specified analysis). **S8 Table**. CAP-3001: Clinical relapse at LFU. **S9 Table**. CAP-3001: Reasons for clinical cure or microbiological eradication not being sustained at the LFU visit (ITT and mITT population).
(ZIP)

S1 Check list (DOC)

S1 Protocol (PDF)
