# Supplementary material for: Ceftobiprole versus ceftriaxone ± linezolid in Community-Acquired Bacterial Pneumonia (CABP): Re-analysis of a randomized, phase 3 study using 2020 FDA guidance
Source: PLoS One. 2025 Jun 24;20(6):e0326758. doi: 10.1371/journal.pone.0326758 (PMC12186948; doi:10.1371/journal.pone.0326758)
Supplement: S1 File — S1 Table. Clinical, radiographic, and microbiologic entry criteria in the 2020 FDA CABP Guidance compared to inclusion criteria for study CAP-3001 (Nicholson et al, 2012). S2 Table. Comparison of the primary endpoint defined in the 2020 FDA CABP guidance with the equivalent pre-specified endpoint (Nicholson et al, 2012). S3 Table. Re-analysis: Clinical success at Day 3 by causative pathogen in accordance with the 2020 FDA Guidance and by MIC (ceftobiprole). S4 Table. Re-analysis: Clinical success at Day 3 by blood culture pathogen at baseline in accordance with the 2020 FDA Guidance. S5 Table. CAP-3001: Analyses of the pre-specified primary study endpoint by prior antibiotic use. S6 Table. CAP-3001: Microbiological eradication at the TOC visit by causative pathogen (mITT and ME populations). S7 Table. CAP-3001: Clinical cure at the TOC visit by blood culture pathogens at baseline (pre-specified analysis). S8 Table. CAP-3001: Clinical relapse at LFU. S9 Table. CAP-3001: Reasons for clinical cure or microbiological eradication not being sustained at the LFU visit (ITT and mITT population). (ZIP) [file pone.0326758.s001.zip › Supporting information/S8 Table. CAP-3001 Clinical relapse at LFU.docx]

**Ceftobiprole Versus Ceftriaxone ± Linezolid in Community-Acquired Bacterial**

**Pneumonia (CABP): Re-analysis of a Randomized, Phase 3 Study Using 2020 FDA Guidance**

Table S8. CAP-3001: Clinical relapse at LFU

Note: Two-sided 95% CI is based on the Normal approximation to the difference of the two proportions.
