# Supplementary material for: Ceftobiprole versus ceftriaxone ± linezolid in Community-Acquired Bacterial Pneumonia (CABP): Re-analysis of a randomized, phase 3 study using 2020 FDA guidance
Source: PLoS One. 2025 Jun 24;20(6):e0326758. doi: 10.1371/journal.pone.0326758 (PMC12186948; doi:10.1371/journal.pone.0326758)
Supplement: S1 File — S1 Table. Clinical, radiographic, and microbiologic entry criteria in the 2020 FDA CABP Guidance compared to inclusion criteria for study CAP-3001 (Nicholson et al, 2012). S2 Table. Comparison of the primary endpoint defined in the 2020 FDA CABP guidance with the equivalent pre-specified endpoint (Nicholson et al, 2012). S3 Table. Re-analysis: Clinical success at Day 3 by causative pathogen in accordance with the 2020 FDA Guidance and by MIC (ceftobiprole). S4 Table. Re-analysis: Clinical success at Day 3 by blood culture pathogen at baseline in accordance with the 2020 FDA Guidance. S5 Table. CAP-3001: Analyses of the pre-specified primary study endpoint by prior antibiotic use. S6 Table. CAP-3001: Microbiological eradication at the TOC visit by causative pathogen (mITT and ME populations). S7 Table. CAP-3001: Clinical cure at the TOC visit by blood culture pathogens at baseline (pre-specified analysis). S8 Table. CAP-3001: Clinical relapse at LFU. S9 Table. CAP-3001: Reasons for clinical cure or microbiological eradication not being sustained at the LFU visit (ITT and mITT population). (ZIP) [file pone.0326758.s001.zip › Supporting information/S1 Table. Clinical, radiographic, and microbiologic entry criteria.docx]

**Ceftobiprole Versus Ceftriaxone ± Linezolid in Community-Acquired Bacterial**

**Pneumonia (CABP): Re-analysis of a Randomized, Phase 3 Study Using 2020 FDA Guidance**

Table S1. Clinical, radiographic, and microbiologic entry criteria in the 2020 FDA CABP Guidance compared to inclusion criteria for study CAP-3001 (Nicholson et al, 2012)

| **2020 FDA CABP Guidance^1^ entry criteria** | **CAP-3001 inclusion criteria^2^** |
| --- | --- |
| **At least two of the following symptoms:**   - Difficulty breathing - Cough - Production of purulent sputum - Chest pain | **At least two of the following criteria:**   - **Dyspnea or tachypnea** - **Cough (new or increased over usual state)** - **Production of purulent sputum** - Note: pleuritic chest pain was assessed but not used as an entry criterion - **Fever or hypothermia or leukocytosis/leukopenia** - Note: hypotension and/or tachycardia were assessed through vital signs but not used as an entry criterion - See above (Dyspnea or tachypnea) - **New-onset hypoxemia on room air** - **Auscultatory rales or pulmonary consolidation** - See above: Fever or hypothermia or leukocytosis/leukopenia |
| **At least two vital sign abnormalities:**   - Fever - Hypothermia - Hypotension - Tachycardia - Tachypnea |  |
| **At least one finding of other clinical signs/laboratory abnormalities:**   - Hypoxemia - Clinical evidence of pulmonary consolidation - An elevated total white blood cell count or leukopenia |  |
| **Radiographic evidence** | X-ray: New radiographic infiltrates (not related to another disease) consistent with the diagnosis of bacterial pneumonia |
| **Appropriate microbiological sample** | Respiratory culture and Gram stain was to be obtained at screening, and to be repeated within 24 hours if an inadequate specimen was obtained. |
| **2020 CABP Draft Guidance^1^ exclusion criteria** | **CAP-3001 exclusion criteria^2^** |
| - Aspiration pneumonia - Hospital-acquired bacterial pneumonia or ventilator-associated bacterial pneumonia - Patients with known bronchial obstruction or a history of post-obstructive pneumonia (this criterion does not exclude patients who have chronic obstructive pulmonary disease) - Patients with primary or metastatic lung cancer - Patients with cystic fibrosis, known or suspected *Pneumocystis jiroveci* pneumonia, or known or suspected active tuberculosis | - Suspected or known pneumonia due to aspiration - HABP or VABP patients were not included - Known bronchial obstruction or a history of post‑obstructive pneumonia (patients with asthma or chronic obstructive pulmonary disease were not excluded provided they met the criteria of acute new-onset pneumonia) - Primary lung cancer or another malignancy to the lungs unless surgically resected - Cystic fibrosis, lung abscess, … active tuberculosis, suspected or known pneumonia due to atypical bacteria…, or *Pneumocystis jiroveci* (carinii) |

CABP = community-acquired bacterial pneumonia; HABP = hospital-acquired bacterial pneumonia;

VABP = ventilator-acquired bacterial pneumonia.

Guidance for Industry. *Community-Acquired Bacterial Pneumonia: Developing Drugs for Treatment*U.S. Department of Health and Human Services, Food and Drug Administration, Center for Drug Evaluation and Research (CDER), June 2020.
