# Supplementary material for: Ceftobiprole versus ceftriaxone ± linezolid in Community-Acquired Bacterial Pneumonia (CABP): Re-analysis of a randomized, phase 3 study using 2020 FDA guidance
Source: PLoS One. 2025 Jun 24;20(6):e0326758. doi: 10.1371/journal.pone.0326758 (PMC12186948; doi:10.1371/journal.pone.0326758)
Supplement: S1 Protocol — (PDF) [file pone.0326758.s003.pdf]

Johnson & Johnson Pharmaceutical Research & Development*

Clinical Protocol

Randomized, Double-Blind, Multicenter Study of Ceftobiprole Medocaril Versus Ceftriaxone with/without Linezolid in Treatment of Subjects Hospitalized With Community-Acquired Pneumonia

Protocol 30982081-CAP-3001; Phase 3

JNJ-30982081 (BAL5788)

Amendment INT-3

[redacted]

This study will be conducted under Food & Drug Administration IND regulations (CFR Part 312).

Issue/Report Date: 22 December 2006
Prepared by: Johnson & Johnson Pharmaceutical Research & Development, L.L.C.
[redacted]

Confidentiality Statement

The information in this document contains trade secrets and commercial information that are privileged or confidential and may not be disclosed unless such disclosure is required by applicable law or regulations. In any event, persons to whom the information is disclosed must be informed that the information is *privileged* or *confidential* and may not be further disclosed by them. These restrictions on disclosure will apply equally to *all* future information supplied to you which is indicated as *privileged* or *confidential*.

INVESTIGATOR AGREEMENT

I have read this protocol and agree that it contains all necessary details for carrying out this study. I will conduct the study as outlined herein and will complete the study within the time designated.

I will provide copies of the protocol and all pertinent information to all individuals responsible to me who assist in the conduct of this study. I will discuss this material with them to ensure that they are fully informed regarding the study drug and the conduct of the study.

Investigator's Signature — Date (Day Month Year)

Name of Investigator (Typed or Printed)

Institution and Address*

Telephone Number*

Coordinating Investigator's Signature* (where required) — Date (Day Month Year)

Name of Coordinating Investigator (Typed or Printed)

[redacted]

* If the address or telephone number of the investigator changes during the course of the study, written notification will be provided by the investigator to the sponsor and will not require protocol amendment(s).

TABLE OF CONTENTS

PROTOCOL AMENDMENTS **6**

SYNOPSIS **28**

TIME AND EVENTS SCHEDULE **34**

ABBREVIATIONS **38**

1. INTRODUCTION **40**
1.1. Background 41
1.1.1. Ceftobiprole 41
1.1.2. Comparator, Ancillary, and Oral Study Drugs 46
1.2. Overall Rationale for the Study 47

2. OBJECTIVES **49**

3. OVERVIEW OF STUDY DESIGN **50**
3.1. Study Design 50
3.2. Study Design Rationale 54

4. STUDY POPULATION **58**
4.1. General Considerations 58
4.2. Inclusion Criteria 58
4.3. Exclusion Criteria 59
4.4. Prohibitions and Restrictions 61

5. RANDOMIZATION AND BLINDING **61**
5.1. Overview 61
5.2. Procedures 62

6. DOSAGE AND ADMINISTRATION **63**
6.1.1. Overview 63
6.1.2. Intravenous and Oral Switch Study Drug Regimen 64
6.1.3. Linezolid/Placebo Therapy for Ceftriaxone-resistant *S. Pneumoniae* and *S. Aureus* 65
6.1.4. Dose Adjustment for Renal Impairment 67

7. COMPLIANCE **67**

8. CONCOMITANT THERAPY **68**

9. STUDY EVALUATIONS **68**
9.1. Study Procedures 68
9.1.1. Overview 68
9.1.2. Prerandomization Phase 68
9.1.3. Double-Blind Treatment Phase 68
9.1.4. Criteria for Intravenous to Oral Switch 70
9.1.5. Follow-Up Phase 72
9.2. Pharmacokinetic Evaluations 72
9.2.1. Sample Collection and Handling 72
9.2.2. Analytical Procedures 73
9.2.3. Pharmacokinetic Parameters 74
9.3. Efficacy Evaluations 74
9.3.1. Clinical Efficacy Assessments 74

TABLE OF CONTENTS (CONTINUED)

9.3.2. Microbiological Procedures and Efficacy Assessments 76
9.3.3. Microbiological Samples and Testing 77
9.3.4. Assessment of Microbiological Outcome 79
9.3.5. Chest X-ray 80
9.4. Efficacy Criteria 80
9.5. Safety Evaluations 81
9.6. Medical Resource Utilization Data Collection 84

10. SUBJECT COMPLETION/WITHDRAWAL 84
10.1. Completion 84
10.2. Discontinuation of Treatment 84
10.3. Withdrawal From the Study 85

11. STATISTICAL METHODS 86
11.1. Sample Size Determination 86
11.2. Pharmacokinetic Analyses 86
11.3. Analyses Methods 87
11.3.1. Definition of Populations 87
11.3.2. Definition of Parameters 87
11.3.3. Subject Baseline Characteristics and Disposition 88
11.3.4. Analysis of Primary Parameter 88
11.3.5. Analysis of Secondary Parameters 89
11.3.6. Analyses of Other Parameters 89
11.4. Safety Analyses 89

12. ADVERSE EVENT REPORTING 90
12.1. Definitions 91
12.1.1. Adverse Event Definitions and Classifications 91
12.1.2. Attribution Definitions 92
12.2. Procedures 92
12.2.1. All Adverse Events 92
12.2.2. Serious Adverse Events 93
12.2.3. Pregnancies 94
12.3. Contacting Sponsor Regarding Safety 94

13. STUDY DRUG INFORMATION 95
13.1. Physical Description of Study Drug(s) 95
13.2. Packaging 95
13.3. Labeling 95
13.4. Preparation and Handling 96
13.5. Drug Accountability 97

14. STUDY-SPECIFIC MATERIALS 98

15. ETHICAL ASPECTS 98
15.1. Study-Specific Design Considerations 98
15.2. Regulatory Ethics Compliance 100
15.2.1. Investigator Responsibilities 100
15.2.2. Independent Ethics Committee or Institutional Review Board 100
15.2.3. Informed Consent 101
15.2.4. Privacy of Personal Data 103

16. ADMINISTRATIVE REQUIREMENTS 104
16.1. Protocol Modifications 104

TABLE OF CONTENTS (CONTINUED)

16.2. Regulatory Documentation 104
16.2.1. Regulatory Approval/Notification 104
16.2.2. Required Prestudy Documentation 104
16.3. Subject Identification Register and Subject Screening Log 105
16.4. Case Report Form Completion 106
16.5. Data Quality Assurance 106
16.6. Record Retention 107
16.7. Monitoring 108
16.8. Study Completion/Termination 109
16.8.1. Study Completion 109
16.8.2. Study Termination 109
16.9. On-Site Audits 109
16.10. Use of Information and Publication 110

17. REFERENCES 112

ATTACHMENTS 116

Attachment 1: Pneumonia Outcomes Research Trial (PORT) Severity Index (PSI) 117

Attachment 2: Preparation and Administration of Intravenous Infusion Solutions of Ceftobiprole Medocaril (500 mg dose) 120

Attachment 3: Procedure for Collection, Culture, Susceptibility Testing, Storage, and Shipment of Microbiological Samples 123

LAST PAGE 127

PROTOCOL AMENDMENTS

Original Protocol issued 18 Jan 2006
Amendments are listed beginning with the most recent amendment.

Amendment INT-3 (22 Dec 2006)

This amendment is considered to be substantial based on the criteria set forth in Article 10(a) of Directive 2001/20/EC of the European Parliament and the Council of the European Union.

Applicable Section(s)	Text Changes<br>(new text in **bold**; deleted text in ~~strikeout~~)	Description of Change / Rationale for Change
Throughout	--	Minor grammatical and formatting errors have been corrected.
Synopsis Dosage and Administration; 6.1.3. Linezolid/Placebo Therapy for Ceftriaxone-resistant S. pneumoniae and S. aureus	**If the incidence of MRSA in CAP isolates is prevalent (ie, greater the 15%) in the local institution or region, or additional subject-specific risk factors are present, such as a history of prior MRSA infection or a surveillance culture positive for MRSA, linezolid**~~Linezolid~~/placebo should be added to cover the possibility of ~~*Staphylococcus aureus* (~~*S. aureus*~~)~~ based on the presence of:…<br>**If the incidence of MRSA in CAP isolates is not prevalent (ie, less than 15%) in the local institution or region, the investigator may wait for definitive confirmation of MRSA sensitive to linezolid in the respiratory specimen collected within 72 hours before randomization prior to adding linezolid/placebo.**	Allows the option to wait for confirmed MRSA prior to adding linezolid in areas of the world where MRSA is not a common CAP pathogen.
	Linezolid/placebo will be discontinued at **48**~~72~~ hours if *S. aureus* ~~or ceftriaxone-resistant pneumococcus~~ is not isolated from the baseline cultures.	Culture results are available in 48 hours or less so the time was reduced from 72 to 48 hours, per FDA request. Ceftriaxone resistant pneumococcus was removed for clarity.

PROTOCOL AMENDMENTS (CONTINUED)

Applicable Section(s)	Text Changes (new text in **bold**; deleted text in ~~strikeout~~)	Description of Change / Rationale for Change
Synopsis Pharmacokinetic Evaluations; 9.2. Pharmacokinetic Evaluations	**All subjects will participate in either sparse or rich pharmacokinetic (PK) sampling**.	Clarification.
Synopsis Pharmacokinetic Evaluations; 9.2.1 Sample Collection and Handling	**Refer to the PK laboratory manual, which will be provided as a separate document (refer to Section 14, Study-Specific Materials) for information regarding handling of biological samples.** The exact dates and times of blood sampling must be recorded in **either** the case report form (CRF) **or the laboratory requisition form as appropriate**.	Rearranged wording in section for clarity.
Synopsis Statistical Methods; 3.1. Study Design	~~An interim analysis is planned when data are available from the first 70% of the clinically evaluable subjects (i.e., 372 subjects) to assess adequacy of sample size. Based on the outcome of the interim analysis, the sample size may be increased.~~	Interim analysis will not be performed.
Time and Events Schedule	Added vital signs, concomitant medications, and adverse events at Day 1 Double-Blind Treatment Phase visit.	Clarification.
	~~[p] Must be performed twice daily while the subject is hospitalized.~~	Minor correction.
	Added hospitalization status from Day 1 through Day 14.	Consistency with case report form.
	Removed Medical Resource Utilization (MRU) data collection from Day 1-14.	Clarification. MRU is only collected at End of Treatment, Test-of-Cure, and Late Follow-Up visits.

PROTOCOL AMENDMENTS (CONTINUED)

Applicable Section(s)	Text Changes (new text in **bold**; deleted text in ~~strikeout~~)	Description of Change / Rationale for Change
3.2. Study Design Rationale	**Atypical pathogens that cause CAP include … to exclude subjects with atypical pneumonias**.<br>**In addition to the above clinical assessments**, … **against the identified atypical pathogen.**	Additional background on *Chlamydophila pneumoniae* and *Mycoplasma pneumoniae* was added for clarity.
4.3. Exclusion Criteria	**Note – Subjects with asthma or chronic obstructive pulmonary disease are not excluded provided they meet the criteria of acute new-onset pneumonia.**	Clarification was needed to differentiate asthma or COPD from bronchial obstruction.
6.1.1. Overview	**Additionally, if it is the local custom for subjects to remain hospitalized for extended periods beyond the need for i.v. therapy for social or logistical reasons, the subject may, in this case, be switched to oral therapy if deemed necessary, while still hospitalized.**	Due to the global nature of the study, allowances were made to accommodate different practices with regard to length of hospitalization.
7. Compliance	Qualified staff **…** including date, start ~~and stop times~~**time** of infusion, ~~and~~ dose**, and whether the entire dose was administered**).	Clarification.
9.1.4. Criteria for Intravenous to Oral Switch	Body temperature improved from baseline (rectal temperature ≥35°C and ≤38.8°C**, axillary temperature ≥35.5°C and ≤37.3°C**, ~~or~~ oral temperature ≥36°C and ≤37.8°C, **or tympanic temperature ≥36.5°C and ≤38.3°C)** for at least 24 consecutive hours in the absence of any antipyretic medications	Clarification.

PROTOCOL AMENDMENTS (CONTINUED)

Applicable Section(s)	Text Changes (new text in **bold**; deleted text in ~~strikeout~~)	Description of Change / Rationale for Change
9.2.3 Pharmacokinetic Parameters	• Pharmacokinetic (PK) parameters will be derived by … will include AUC_τ, AUC_∞, C_{max}, **CL, and Ae** and $t_{1/2}$ of ceftobiprole. In addition, time above minimum inhibitory concentration (MIC) of ceftobiprole will be determined (assuming MIC of 4 µg/mL). Urine concentrations of ceftobiprole will be used to determine urinary excretion.<br><br>• **CL Total systemic clearance, estimated by dose/AUC_τ**<br>• ~~$t_{1/2}$ terminal elimination half-life determined by linear least squares regression of the plasma concentration versus time curve data in the post-distributive phase~~<br>• **Ae Amount excreted into the urine, calculated by the product of urinary volume times the urinary concentration over the collection interval**	Addition and clarification of PK parameters for ceftobiprole.

PROTOCOL AMENDMENTS (CONTINUED)

Applicable Section(s)	Text Changes (new text in **bold**; deleted text in ~~strikeout~~)	Description of Change / Rationale for Change
9.3.3. Microbiological Samples and Testing	Respiratory specimens should be obtained within 24 hours before the first dose of study drug. In the event that a subject has received nonstudy antibiotics before study enrollment, respiratory cultures obtained **before, or as soon as possible, but within 24 hours after the**~~before~~ start of **nonstudy** antibiotic treatment must be available. **This culture must repeated, if necessary, so that a respiratory specimen is collected within 24 hours before the first dose of study drug.**	Additional information was received that antibiotic use within 24 hours of culture and gram stain minimally effects yield or results.
	At least 2 sets of aerobic/anaerobic blood cultures must be obtained according to local practice for each subject, within 24 hours before the start of treatment. In the event that a subject has received nonstudy antibiotics before study enrollment, blood cultures obtained **before, or as soon as possible, but within 24 hours after the** ~~before~~ start of **nonstudy** antibiotic treatment must be available. **These cultures must be repeated, if necessary, so that a blood culture is collected within 24 hours before the first dose of study drug.**	Coordination with change to sputum collection timing.

PROTOCOL AMENDMENTS (CONTINUED)

Applicable Section(s)	Text Changes (new text in **bold**; deleted text in ~~strikeout~~)	Description of Change / Rationale for Change
9.3.5. Chest X-ray	Chest X-rays (preferably both posteroanterior and lateral views) will be obtained according to the Time and Events Schedule, and at any other time deemed appropriate by the investigator. Chest X-rays should be interpreted by ~~an experienced physician~~**the investigator, a pulmonologist, or radiologist** and interpreted with respect to the baseline chest X-ray for clinical management of the subject during the study. Chest X-rays obtained from the predose~~, IOS, EOT,~~ **and** TOC~~, and LFU~~ visits will ~~also~~ be ~~transferred to a~~ **interpreted at a** central location ~~for reading and interpretation~~. **The chest x-rays will be centrally read to confirm the presence of a qualifying infiltrate at baseline and to confirm the site interpretation at TOC.**	Further detail added for clarification.
9.5. Safety Evaluations	**Serum for Mycoplasma pneumoniae and Chlamydia pneumoniae antibody titers**.	Added for consistency.
11. Statistical Methods	Section 11.2 deleted. Subsequent subsections have been renumbered.	Interim analysis will not be performed.
11.3.5. Analysis of Secondary Parameter	The 30-day pneumonia-specific mortality rate will be analyzed to compare the 2 treatment groups using a ~~2-sample t-test~~**Fisher's exact test**.	Correction.
17. References	Added references 38, 39, and 40. Subsequent references have been renumbered.	Clarification.

PROTOCOL AMENDMENTS (CONTINUED)

Applicable Section(s)	Text Changes (new text in **bold**; deleted text in ~~strikeout~~)	Description of Change / Rationale for Change
Attachment 1. Pneumonia Outcomes Research Trial (PORT) Severity Index (PSI)	**Complete the following to determine the PSI:** … **Step 2: Continue to caculate the PSI if any of the items above in step 1 are "YES".**~~After diagnosing the subject with community-acquired pneumonia, complete the following to determine PSI.~~	Clarification.
Attachment 3. Procedure for Collection, Culture, Susceptibility Testing, Storage, and Shipment of Microbiological Samples	**A: Susceptibility Testing of Ceftobiprole (~~BAL/~~BPR)**<br>MIC range: **BPR Etest strips are either 0.002 to 32 µg/mL or** 0.016 – 256 µg/mL ~~labeled with BPR~~.<br>The 3-letter code BPR ~~(BAL)~~ will be …<br>To validate E-test runs and disk diffusion testing, **at least 1 closely related** CLSI quality control strain~~s~~ should be tested ~~with each use~~**in parallel with the subject's isolate. If susceptibility testing is set up more than once a day, ceftobiprole QC should be tested at least daily if there are subject isolates to be tested. Quality control strain results should be** ~~to ensure that values obtained are~~ within the ~~tentative~~ **CLSI** QC ranges **listed below**.<br>Interpret measured zones of inhibition **or Etest result** by reference to the following table ~~of breakpoints~~ to classify test isolate as Susceptible (S)~~, Intermediate (I), or Resistant (R)~~.	Clarification.

Amendment INT-2 (16 Aug 2006)

This amendment is considered to be substantial based on the criteria set forth in Article 10(a) of Directive 2001/20/EC of the European Parliament and the Council of the European Union.

PROTOCOL AMENDMENTS (CONTINUED)

Applicable Section(s)	Text Changes (new text in **bold**; deleted text in ~~strikeout~~)	Description of Change / Rationale for Change
Throughout	--	Minor grammatical and formatting errors have been corrected.
Synopsis Objectives; 2. Objectives; 11.4.2. Definition of Parameters	--	The organization and order of the secondary and other objectives and endpoints were revised for consistency with other protocols in the ceftobiprole program. In addition, the secondary endpoints were put in an order based upon their clinical relevance.
Synopsis Overview of Study Design;	… 18 years of age or older with ~~hospitalized~~ CAP … require hospitalization	Statement was revised for clarity.
Synopsis Overview of Study Design; Time and Events Schedule (column heading and footnote i); 3.1. Study Design; 9.1.1. Overview; 9.1.4. Criteria for Intravenous to Oral Switch	… end-of-treatment (EOT) visit (within 48 hours ~~of~~**after** the end of therapy), …	Statement was revised for clarity.
Synopsis Overview of Study Design; 3.1. Study Design	The total duration of the study participation for a subject will be ~~approximately 35~~**up to 49** days.	The study duration was corrected to account for up to 14 days on study drug plus 35 days for the late follow-up.
Synopsis Study Population; 3. Overview of Study Design	Approximately 670 subjects, 18 years of age or older, ~~hospitalized~~**with** CAP … require hospitalization …	Statement was revised for clarity.
Synopsis Dosage and Administration; 6.1.1. Overview	**Colored sleeves will cover infusion bags and line tubings as needed to maintain the blind.**	Statement added to provide additional details regarding the way the study blind will be maintained.
Synopsis Dosage and Administration; 3.1. Study Design, 6.1.1. Overview	**At the discretion of the investigator, subjects may receive study drug infusions as outpatients …**	Statement was added for clarity.

PROTOCOL AMENDMENTS (CONTINUED)

Applicable Section(s)	Text Changes<br>(new text in **bold**; deleted text in ~~strikeout~~)	Description of Change / Rationale for Change
Synopsis Dosage and Administration; 6.1.2. Intravenous and Oral Switch Study Drug Regimen	Ceftobiprole will be administered at a dose of 500 mg **in 250 mL of 5% dextrose in water (D_5W)** … [e.g., saline, Ringer's **Lactate** solution]). … Ceftriaxone will be administered at a dose of 2 g **in 50 mL of D_5W** … For subjects randomized to ceftobiprole, a 30-minute i.v. infusion of placebo **in 50 mL of D_5W** …	Added for clarification and for consistency with the study diagram and text.
	For subjects randomized to ceftriaxone, a 120-minute i.v. infusion of placebo **of 250 mL of D_5W** will be given **every 8 hours** concomitantly with ceftriaxone**, and will begin with the first dose of ceftriaxone.**	Added for clarification and consistency.
	~~Subjects will receive additional 120-minute placebo infusions every 8 hours.~~**Note: The 30-minute infusion should be administered first (placebo or ceftriaxone), followed by the 120-minute infusion (ceftobiprole or placebo), to ensure that active treatment is delivered to the subject as soon as possible.**	Statement was deleted and combined with the previous bullet for consistency with the rest of the text in the section. Statement regarding the order of the infusions was added as it optimizes the delivery time of active drug to subjects.
Synopsis Dosage and Administration; 6.1.3. Linezolid/ Placebo Therapy for Ceftriaxone-resistant *S. pneumoniae* and *S. aureus*	Antibiotic coverage of ceftriaxone-resistant *S. pneumoniae* (**CRSP**~~CTRP~~):	The abbreviation was corrected for consistency.
	**If the subject meets the criteria listed above but a Gram stain cannot be immediately obtained … If the results do not show gram-positive cocci …**	Statements were added to provide additional clarification of the process.
	Linezolid will be administered to those subjects randomized to ceftriaxone, at a dose of 600 mg **in** ~~(~~300 mL~~)~~ **of D_5W** …	Added for clarification and for consistency

PROTOCOL AMENDMENTS (CONTINUED)

Applicable Section(s)	Text Changes (new text in **bold**; deleted text in ~~strikeout~~)	Description of Change / Rationale for Change
	Placebo **of 300 mL of normal saline (NaCl 0.9% solution)** will be administered to those subjects randomized to ceftobiprole, at a volume of 300 mL …	Normal saline is being used as the placebo solution if linezolid is added for suspected MRSA in order to limit the amounts of excess free water that the subject is receiving.
	**Normal saline (NaCl 0.9% solution) will replace D_5W as the placebo solution for ceftobiprole …**	Statement added to clarify the switch to normal saline as the placebo solution for ceftobiprole if linezolid is added.
	--	A table was added to clearly show the changes to the placebo solution if linezolid is added to the study drug therapy.
Synopsis Efficacy Evaluations/Criteria; 9.4. Efficacy Criteria	--	The efficacy criteria were revised to be consistent with the changes made to the secondary objectives.
Synopsis Pharmacokinetic Evaluations; 9.2.1. Sample Collection and Handling	Approximately 120 subjects (approximately 60 subjects on ceftobiprole~~plus placebo~~ treatment and approximately 60 subjects on ~~linezolid plus~~ceftriaxone **with or without linezolid**) …	Changes were made for clarification and consistency.
	At selected sites for rich PK sampling ~~(North America, Europe, Asia)~~:<br>At sites for sparse PK sampling ~~(North America, Europe, Asia)~~:	Specific regions were removed from the subtitles as all countries will participate in the PK evaluations.
	On Day 4±1, one **3**~~6~~ mL blood sample …<br>On study Day 1, **two**~~2~~ PK blood samples (**3**~~6~~ mL each) …<br>On Day 4±1, another PK blood sample (**3**~~6~~ mL) …	The amount of blood drawn for PK analysis was decreased from 6 mL to 3 mL for consistency with the Phase 1 program.

PROTOCOL AMENDMENTS (CONTINUED)

Applicable Section(s)	Text Changes (new text in **bold**; deleted text in ~~strikeout~~)	Description of Change / Rationale for Change
	On study Day 4±1, one 3 mL blood sample will be obtained immediately before the start of the **120-minute** morning infusion (predose); … Subsequently, urine will be collected during the period of 0 to 8 hours after **the** start of the **120-minute** morning infusion.	Added for clarification and consistency.
	On Day 1, two PK blood samples (3 mL each) will be taken**; one** at approximately 2 hours (**or immediately** before ~~stop~~**the end** of the first ~~ceftobiprole or ceftriaxone~~**120-minute** infusion) and **the other** … but starting at least 30 minutes after the onset of this **120-minute** morning infusion.	Added for clarification and consistency.
Synopsis Statistical Methods; 11.4.4. Analysis of Primary Parameter	In order to claim noninferiority, the lower bound of the 2-sided 95% confidence interval must be ≥~~at least~~ -10%.	Wording was changed for clarity
Time and Events Schedule	**Days 4 to 14** i.v. oral switch (IOS) (optional)[f]	Days were added to the column heading for clarification and consistency.
	Blood sample for mycoplasma and chlamydia ~~*pneumoniae*~~ titers	The types of evaluations were corrected.
	**--**	Added an X to the table to show that drug accountability will be conducted at EOT to be consistent with the text.

PROTOCOL AMENDMENTS (CONTINUED)

Applicable Section(s)	Text Changes (new text in **bold**; deleted text in ~~strikeout~~)	Description of Change / Rationale for Change
	[v]**Repeat within 24 hours if an inadequate specimen is obtained.**	Footnote "v" was added to the Respiratory culture & Gram stain procedure for clarification and for consistency with the text; subsequent footnotes in the Time and Events Schedule were renumbered.
	--	A section on Pharmacokinetic (PK) Sampling, which includes Rich PK blood sampling, PK urine sampling, and Sparse PK blood sampling, was added to the table with corresponding footnotes (z to dd) to be consistent with the text.
	[b]… At each on-**treatment**~~therapy~~ assessment …	Footnote "b" was changed for consistency with the Time and Events Schedule column heading.
Time and Events Schedule; 9.1.3. Double-Blind Treatment Phase; 9.5. Safety Evaluations	[s]**Subjects with serum sodium levels outside of the normal range at baseline or during the study should have their electrolytes measured on a daily basis or more often as clinically indicated until the serum sodium level is within the normal range.**	Statement was added so that there is an ongoing evaluation of subjects to ensure that electrolytes stay within the normal range; subsequent footnotes in the Time and Events Schedule were renumbered.

PROTOCOL AMENDMENTS (CONTINUED)

Applicable Section(s)	Text Changes (new text in **bold**; deleted text in ~~strikeout~~)	Description of Change / Rationale for Change
Time and Events Schedule; 9.1.4. Criteria for Intravenous to Oral Switch	[f]At any time after 3 days (72 hours) of i.v. **therapy**~~studydrug~~ (between Days **4**~~3~~ and 14**) unless the subject was bacteremic upon enrollment,** …<br><br>[y]If blood cultures obtained at the enrollment visit (baseline ~~assessment~~) are positive for a pathogen, blood cultures should be repeated and must be negative after a minimum of ~~5~~**3** days (~~120~~**72** hours) …	Revised for clarification and for consistency with the new column heading in the Time and Events Schedule.<br><br>Timing was changed to reflect clinical practice as results are typically available within 3 days.
Time and Events Schedule; 9.5. Safety Evaluations	[o]**… The vital signs that reflect the highest temperature recorded for the subject on that day should be captured in the case report form (CRF).**	Language was added to clarify which vital signs should be recorded on the CRF.
Abbreviations	**NOTE: Abbreviations for pharmacokinetic parameters are defined …**<br>**AUC … area under …**<br>**GCP … Good Clinical Practice**<br>**ICH … International Conference …**<br>**IEC … Independent Ethics …**<br>**IRB … Institutional Review …**<br>**MedDRA … Medical Dictionary …**<br>**T>MIC … percentage of dosing …**	Added for completeness.
	~~cSSSI~~	Abbreviations deleted (only used once).
	**D_5W … 5% dextrose in water**	Added for consistency with changes to the text.
	PK … ~~PK~~**pharmacokinetic**	Corrected.

PROTOCOL AMENDMENTS (CONTINUED)

Applicable Section(s)	Text Changes (new text in **bold**; deleted text in ~~strikeout~~)	Description of Change / Rationale for Change
Abbreviations; 1. Introduction; 1.1.1. Ceftobiprole; 1.2. Overall Rationale for the Study; 15.1. Study-Specific Design Considerations	…~~cephalosporin~~**ceftriaxone**-resistant …	The change was made because 1) ceftobiprole is also a cephalosporin and 2) the term, ceftriaxone-resistant, is now consistent with the abstract and text in the cited reference.
1. Introduction	--	Reference 4 added for Investigator's Brochure; subsequent references were renumbered.
1.1.1. Ceftobiprole	Due to its unusually potent … ceftobiprole is ~~highly~~ active … as well as exhibiting ~~very~~ useful in vitro activity … Ceftobiprole is ~~highly~~ active, however, against …	Statements revised to be consistent with available data.
	**Please refer to the Investigator's Brochure for a complete description of available toxicology data.[4]**	Statement was added as a reference for the toxicology data.
	For subjects receiving placebo there was a decrease in QTcF of 9 msec on Day 1 and 15 msec on Day 8 compared with baseline**.** **Correction**~~correction~~ of QT for heart rate …	Statement was separated into 2 sentences for clarity.
1.1.2. Comparator, Ancillary, and Oral Study Drugs	~~Ceftriaxone~~**Cefuroxime** is recommended in treatment guidelines for treating hospitalized patients with pneumonia.[1,2]	The drug name was corrected.
3. Overview of Study Design	~~Subjects will have CAP that requires hospitalization and are expected to require treatment with i.v. antibiotics for a minimum of 3 days (72 hours).~~	Statement was removed because it was a duplicate of a previous statement made in the paragraph.
3.1. Study Design – Study Diagram	--	Changes were made for clarification and consistency with the text.

PROTOCOL AMENDMENTS (CONTINUED)

Applicable Section(s)	Text Changes (new text in **bold**; deleted text in ~~strikeout~~)	Description of Change / Rationale for Change
	[b]**The 30-minute infusion should be administered first (placebo or ceftriaxone), followed by the 120-minute infusion (ceftobiprole or placebo).** The target duration of study drug treatment (i.v. and oral) …	The statement was added to footnote “b” as clarification.
3.2. Study Design Rationale	Ceftriaxone was chose as the primary comparator in this study because it is a standard first-line therapy for subjects~~, and is generally preferred over cefotaxime and other cephalosporins in treating patients~~ with CAP infections who require hospitalization.[1,25] Ceftriaxone also has ~~a similar~~**the same** route of administration (i.v.) **as ceftobiprole**.	Statements were changed for clarity.
	… a switch from i.v. study drugs to oral cefuroxime axetil is allowed **after a minimum of 3 days of i.v. therapy**, but only for those subjects who meet protocol-specified criteria …	Wording was added for clarity.
4.3. Exclusion Criteria	History of or suspected condition … [NYHA] Class 4)[44]	Reference citation deleted.
	History of or suspected hypersensitivity … **Subjects in whom cephalosporins would be used as part of normal clinical practice are not excluded.**	The statement was added as clarification.
5.2. Procedures	… subject number and treatment **regimen**~~code~~	Wording was changed for clarity.
	~~The IVRS will then also assign a medication kit that matches the treatment code to which the subject has been randomized.~~	Statement was deleted because it was incorrect.
6.1.4. Dose Adjustment for Renal Impairment	**Subjects whose Cl_{Cr} level falls to ≤10 mL/min are to be discontinued from the study.**	Statement added to ensure the safety of subjects enrolled in the trial.

PROTOCOL AMENDMENTS (CONTINUED)

Applicable Section(s)	Text Changes (new text in **bold**; deleted text in ~~strikeout~~)	Description of Change / Rationale for Change
7. Compliance	The subject will be instructed to return the **study drug** container and any remaining study drug at the **EOT**~~TOC~~ visit.	Text was changed for consistency.
	~~Study personnel will examine the medication container and record the amount of study drug remaining on the CRF.~~	Statement was removed because drug accountability is not captured on the CRF.
9.1.1. Overview	The total volume of blood collected for laboratory evaluations throughout this study is approximately ~~142~~**121** mL for each subject (50 mL for safety, 10 mL for **mycoplasma**~~legionella~~ and chlamydia **titers**~~pneumonia~~, 30 mL for blood cultures, 10 mL for repeat blood samples, and **up to 21**~~42~~ **mL** for PK sampling **[only 9 mL of blood will be drawn for those subjects participating in the sparse PK sampling, thereby yielding a total blood volume collected of 109 mL]**).	The total blood volume collected has been recalculated to be consistent with the revised volume per PK sample, which was decreased from 6 mL to 3 mL.<br><br>The types of evaluations were corrected.
9.2.1. Sample Collection and Handling	Refer to **the PK laboratory manual, which will be provided as a separate document (refer to Section 14, Study-Specific Materials)**~~Attachment 4 and 5~~ for further information regarding handling of biological samples.	Statement was revised because Attachments 4 and 5 have been removed from the protocol and will be included as part of a PK laboratory manual, which will be a separate document from the protocol.
9.2.2. Analytical Procedures;	Plasma samples … ceftobiprole ~~and its prodrug ceftobiprole medocaril~~ … Urinary concentrations of ceftobiprole ~~and its prodrug~~ …	In previous Phase 3 studies, ceftobiprole medocaril concentrations were not detectable. Because the prodrug does not have any bactericidal activity, it will not improve the characterization of the PK of ceftobiprole.

PROTOCOL AMENDMENTS (CONTINUED)

Applicable Section(s)	Text Changes<br>(new text in **bold**; deleted text in ~~strikeout~~)	Description of Change /<br>Rationale for Change
9.2.3. Pharmacokinetic Parameters	~~Assessment of the kinetic parameters of prodrug ceftobiprole medocaril will depend on measurable plasma concentrations.~~ Urine concentrations of ceftobiprole ~~and its prodrug~~ …	Pharmacokinetic parameters will not be estimated for the prodrug ceftobiprole medocaril because PK concentrations will no longer be measured.
	~~If 50% of the values at a given time point are below the limit of quantitation (BLQ),~~ …<br>~~For PK evaluation, BLQ values immediately after the start of administration will be set to 0, … if the following sample(s) show a concentration above the BLQ value.~~	The text was deleted because the details regarding the PK analysis are too excessive for the protocol. This information is included in PK analysis guidelines and is not necessary for the protocol.
9.3.5. Chest X-ray	Chest X-rays (preferably both posteroanterior and lateral views) will be obtained **according to the Time and Events Schedule**~~at baseline and TOC~~ and at any other time deemed appropriate by the investigator. Chest X-rays should be interpreted by ~~a competent~~ **an experienced** physician and interpreted with respect to the baseline chest X-ray **for clinical management of the subject during the** ~~trial~~**study. Chest X-rays obtained from the predose, IOS, EOT, TOC, and LFU visits will also be transferred to a central location for reading and interpretation.**	A general reference has been made to the Time and Events Schedule instead of listing each individual visit when the chest X-ray is conducted.<br>Central reading of the chest X-rays has been added to the protocol.<br>The qualifications of the individual assigned to read the chest X-ray has been clarified.
9.4. Efficacy Criteria	For the definition of these parameters see Section ~~11.3.2~~**11.4.2**, Definition of Parameters.	Section number was corrected for consistency with the text.

PROTOCOL AMENDMENTS (CONTINUED)

Applicable Section(s)	Text Changes (new text in **bold**; deleted text in ~~strikeout~~)	Description of Change / Rationale for Change
9.5. Safety Evaluations	Clinical Laboratory Tests<br>The following tests will be performed by the **central**~~local~~ laboratory.<br><br>*If positive dipstick results are obtained for blood or protein, a microscopic examination of sediment is required~~ at the local laboratory~~.	Changes made to correctly state that clinical laboratory tests will be evaluated by a central laboratory.
	**Note: Blood samples for WBC with differential and chemistries (electrolytes and liver function tests) for evaluation of subject eligibility, as well as other samples deemed essential by the investigator for clinical management may be sent to the local laboratory, as necessary.**	Added to clarify the roles of the central and local laboratories.
11.2. Interim Analysis to Adjust Sample Size	The above sample size estimate depends on a few assumptions:<br>• a common clinical cure rate of 90% in both treatment groups,<br>• a common ~~non~~evaluability rate of **80**~~20~~%.	The changes were made to be consistent with the changes made to the conduct of the interim analysis.
11.4.1. Definition of Populations	Safety Population: All randomized subjects who **are exposed to the study drug**~~received at least 1 dose of study drug~~**.**	The statement was changed to account for all subjects who took study drug including those who took less than the full dose.
11.4.2. Definition of Parameters	Secondary<br>• Microbiological eradication rate, **defined** as the ratio …	Word added for clarity
11.4.3. Subject Baseline Characteristics and Disposition	~~Medical resource utilization data will be summarized descriptively.~~	Statement was removed because it already appears in Section 11.4.6, Analyses of Other Parameters.

PROTOCOL AMENDMENTS (CONTINUED)

Applicable Section(s)	Text Changes<br>(new text in **bold**; deleted text in ~~strikeout~~)	Description of Change / Rationale for Change
11.4.4. Analysis of Primary Parameter	~~The 2-sided 95% confidence interval … combined test statistics of data from 2 stages …~~	The interim analysis will now be performed in a blinded fashion and a 2-stage analysis is no longer needed.
13.1. Physical Description of Study Drug(s)	The study drugs consist of ceftobiprole medocaril 500 mg, ceftriaxone 2 g, linezolid 600 mg, and cefuroxime axetil **500 mg**, …	Added for consistency and clarification.
14. Study-Specific Materials	• **PK laboratory manual**	Added the PK laboratory manual to the list of study supplies because Attachments 4 and 5 have been removed from the protocol to be maintained in a separate document.
17. References	1. Mandell L, Bartlett J, … ~~CID~~**Clin Infect Dis** 2003;31:1405-1433.	Journal name corrected.
	3. Davies T, Bush K. Activity of BAL9141 and other β-lactams against *Streptococcus pneumoniae* in U.S. clinical isolates with defined substitutions in penicillin binding proteins (PBP) PBP1a, PBP2b, and PBP2x. ~~ICAAC 2005 (submitted)~~**Antimicrob Agents Chemother 2006 (in press)**.	Reference updated.
	14. Ginesu F, ~~Pirana~~**Pirina** P, … J Chemother 1997;**9:**285-292.	Author's name corrected; volume number added.
	~~43. File TM, Garau J, Jacobs MR, et al. … Int J Antimicrob Agents 2005;25(2):110-119.~~	Reference deleted (no citation in the text).

PROTOCOL AMENDMENTS (CONTINUED)

Applicable Section(s)	Text Changes (new text in **bold**; deleted text in ~~strikeout~~)	Description of Change / Rationale for Change
Attachments - Attachment 1. Pneumonia Outcomes Research Trial (PORT) Severity Index (PSI)	--	All text on the first page after the initial statement, "After diagnosing the subject with community-acquired pneumonia, complete the following to determine PSI." has been deleted because subjects will not need a minimum PSI score to be enrolled in the study.
Attachments - Attachment 3. Procedure for Collection, Culture, Susceptibility Testing, Storage, and Shipment of Microbiological Samples	--	The interpretations for intermediate and resistant were removed from the table entitled Proposed breakpoint for ceftobiprole (BAL9141) because J&JPRD does not have the data to support these interpretations.<br><br>The disk diffusion zone sizes were updated to the approved CLSI zone sizes for some of the QC bacterial strains in the table entitled Quality control.
	The **E-test** MIC range is **either** 0.016 to 256 µg/mL_**or 0.002 to 32 µg/mL**.	The text was changed because once the supply of 256 µg/mL E-test strips is done, future supplies of E-test strips will be manufactured with the 32 to 0.002 ug/mL range.
Attachments Attachment 4. Pharmacokinetic Sample Collection and Handling; Attachment 5. Labeling Instructions for Pharmacokinetic Samples	--	Attachments 4 and 5 have been removed from the protocol and will be included as part of a PK laboratory manual, which will be a separate document from the protocol.

PROTOCOL AMENDMENTS (CONTINUED)

Applicable Section(s)	Text Changes (new text in **bold**; deleted text in ~~strikeout~~)	Description of Change / Rationale for Change

<u>Amendment INT-1</u> (2 Mar 2006)

This amendment is considered to be non-substantial based on the criteria set forth in Article 10(a) of Directive 2001/20/EC of the European Parliament and the Council of the European Union

Applicable Section(s)	Text Changes (new text in **bold**; deleted text in ~~strikeout~~)	Description of Change / Rationale for Change
General	Minor formatting and spelling errors were corrected. They are not specified in this Amendment table nor shown in the text.	
Synopsis Overview of Study Design; Study Population; Statistical Methods; 3. Overview of Study Design; 3.1. Study Design; 4.1. General Considerations ; 11.1. Sample Size Determination; 11.2. Interim Analysis to Adjust Sample Size	1:1 randomization and increased sample size was added in order to provide sufficient power to meet the primary endpoint in both the CE and ITT population.<br>Approximately 670 instead of 500 subjects will be randomized to achieve 532, instead of 399 of clinically evaluable subjects (266 in each group, instead of 133 in the comparator group and 266 in the ceftobiprole group).	
Synopsis, Pharmacokinetic Evaluations; 9.2. Pharmacokinetic Evaluations; 11.3. Pharmacokinetic Analyses	Sections were added for PK population to provide a more robust PK/PD population for the ceftobiprole pneumonia indications (NP + CAP)<br>All subsequent sections have been renumbered.	
Synopsis Objectives; 2. Objectives	**To assess the PK of ceftobiprole in subjects treated with ceftobiprole.**	Added as a result of inclusion of PK evaluations in the protocol.
3.2. Study Design Rationale	**In order to develop a PK structure model … sampling in additional subjects are needed.**<br>**The combination of rich PK sampling and sparse PK sampling … on plasma ceftobiprole concentrations.**	Rationale for including PK assessments was added.

PROTOCOL AMENDMENTS (CONTINUED)

Applicable Section(s)	Text Changes (new text in **bold**; deleted text in ~~strikeout~~)	Description of Change / Rationale for Change
9.1.1. Overview	The total volume of blood collected for laboratory evaluations throughout this study is approximately ~~100~~ **142** mL for each subject… **and 42 for PK sampling.**	The PK sampling amount was added as a result of inclusion of PK evaluations in the protocol.
9.1.4 Criteria for Intravenous. to Oral Switch	Body temperature **improved from baseline** ~~within the normal range~~…	Clarification for temperature criteria for oral switch.
Attachment 3 Procedure for Collection, Culture, Susceptibility Testing, Storage, and Shipment of Microbiological Samples	Sensitive ~~>~~≥ 20mm…<br>Intermediate **17-19 mm**…<br>Resistant **≤16mm**…	Information on disk diffusion interpretation expanded.
Attachment 4 Pharmacokinetic Sample Collection and Handling; Attachment 5. Labeling Instructions for Pharmacokinetic Samples	Added as a result of inclusion of PK evaluations in the protocol.	

Randomized, Double-Blind, Multicenter Study of Ceftobiprole Medocaril Versus Ceftriaxone with/without Linezolid in Treatment of Subjects Hospitalized With Community-Acquired Pneumonia

SYNOPSIS

OBJECTIVES:

The primary objective is to demonstrate the noninferiority of ceftobiprole medocaril compared with ceftriaxone with/without linezolid (comparator) with respect to the clinical cure rate in subjects hospitalized with community-acquired pneumonia (CAP) at the test-of-cure (TOC) visit.

The secondary objectives are:

- To compare the microbiological eradication rate following treatment with ceftobiprole versus the comparator in subjects with hospitalized CAP at the TOC visit
- To compare the clinical cure rate following treatment with ceftobiprole versus the comparator of hospitalized CAP subjects that require mechanical ventilation within the first 48 hours of enrollment
- To compare the microbiological eradication rate following treatment with ceftobiprole versus the comparator of hospitalized CAP subjects that require mechanical ventilation within the first 48 hours of enrollment
- To compare the clinical and microbiological relapse rates following treatment with ceftobiprole versus the comparator in subjects with hospitalized CAP at the late follow-up (LFU) visit
- To compare the 30-day pneumonia-specific mortality rates following treatment with ceftobiprole or the comparator of hospitalized CAP subjects

Other objectives are:

- To assess the pharmacokinetics (PKs) of ceftobiprole in subjects treated with ceftobiprole
- To collect medical resource utilization (MRU) data that may be used in future economic modeling

Safety/tolerability objectives are:

- To characterize the safety and tolerability of treatment with ceftobiprole in subjects hospitalized with CAP.

Hypotheses

Efficacy: The clinical cure rate, with respect to treatment with ceftobiprole with/without switch to oral therapy, will be noninferior to a regimen of the comparator, with/without a switch to oral therapy, in the clinically evaluable subjects hospitalized with CAP.

Safety and tolerability: The safety and tolerability of treatment with ceftobiprole will be similar to a regimen of the comparator in hospitalized CAP subjects.

OVERVIEW OF STUDY DESIGN:

This randomized, double-blind, comparator-controlled, multicenter study is designed to assess the efficacy and safety of ceftobiprole compared with the comparator in subjects 18 years of age or older with CAP of sufficient severity to require hospitalization and treatment with intravenous (i.v.) antibiotics for a minimum of 3 days (72 hours). The study will consist of a prerandomization phase (baseline), a double-blind treatment phase with an end-of-treatment (EOT) visit (within 48 hours after the end of therapy), and a follow-up phase with a TOC/early termination visit 7 to 14 days after the last

SYNOPSIS (CONTINUED)

dose of study drug, and a LFU visit 28 to 35 days after the last dose of study drug. The total duration of the study participation for a subject will be up to 49 days.

Approximately 670 subjects will be randomized to achieve 266 clinically evaluable subjects receiving ceftobiprole and 266 clinically evaluable subjects receiving the comparator. This study will be conducted in a manner to ensure that investigators, those administering study drug, and subjects are blinded to treatment regimen. Placebo will be used to maintain the blind for both the cephalosporin (ceftobiprole and ceftriaxone) and linezolid therapies. Central randomization will be used to randomly assign subjects in a 1:1 ratio to receive ceftobiprole or the comparator. Subjects will be stratified by the Pneumonia Outcomes Research Trial (PORT) Severity Index (PSI) score at randomization (PSI ≤90 versus PSI ≥91). Subjects will also be stratified by the need for anti-staphylococcal therapy (placebo or linezolid) based on signs, symptoms, and medical history at enrollment. All subjects should receive a minimum of 3 days (72 hours) of the i.v. study drug(s) and should continue to receive i.v. therapy while hospitalized (see DOSAGE AND ADMINISTRATION below). The total duration of antibiotic treatment (i.v. and oral) will be at least 5 days and up to a maximum of 10 days. If more than 10 days are necessary to complete treatment, therapy may be further extended to a maximum of 14 days for subjects with a history of persistent bacteremia or necrotizing pneumonia. Initially, all subjects will be hospitalized.

STUDY POPULATION:

Approximately 670 subjects, 18 years of age or older, with CAP severe enough to require hospitalization (PSI ≤90 versus PSI ≥91) and treatment with i.v. antibiotics for at least 3 days (72 hours), will participate in this study to achieve 532 clinically evaluable subjects; 266 in each group.

DOSAGE AND ADMINISTRATION*:

Dose and Schedule of Study Drug and Comparator

The unblinded pharmacist will be responsible for preparation of the study drug for each subject in such a way that investigators and staff remain blinded to the study drug being administered. Colored sleeves will cover infusion bags and line tubings as needed to maintain the blind. All subjects should receive a minimum of 3 days (72 hours [9 doses of ceftobiprole or 3 doses of ceftriaxone; with placebo administered as described below to maintain the blind]) of the i.v. study drug and should continue on i.v. therapy while hospitalized. At the discretion of the investigator, subjects may receive study drug infusions as outpatients or through a home-care agency, provided they meet all other protocol requirements, but all i.v. study drug infusions must be administered and monitored by a qualified nurse, a qualified member of the study staff, or an infusion specialist. However, if after 3 days (72 hours) the subject's signs and symptoms have improved from baseline and the subject will be discharged from the hospital, the i.v. therapy may be discontinued at the investigator's discretion and the subject treated with oral cefuroxime axetil 500 mg every 12 hours.

All subjects should receive the first dose of study drug as quickly as possible after randomization. In order to ensure subject safety and to avoid potential overdose, if a subject has received a nonstudy antibiotic before enrollment, the timing of the first dose of study drug may be delayed so it is consistent with the recommended dosing interval of the nonstudy antibiotic. In the event that a subject received a nonstudy, beta-lactam antibiotic (e.g., penicillin, cephalosporin, carbapenem) before enrolling in the study, the timing of the first dose of study cephalosporin (ceftobiprole or ceftriaxone) may be delayed.

* This section of the protocol has been revised. Please refer to the section of this document entitled PROTOCOL AMENDMENTS (Amendment INT-3, 22 December 2006) for a detailed description of the specific changes.

SYNOPSIS (CONTINUED)

Intravenous and Oral Switch Study Drug Regimen

Study drug (i.v. plus oral) will be administered for 5 to 10 days. Therapy can be extended to a maximum of 14 days after approval by the sponsor's Medical Monitor, for subjects with a history of persistent bacteremia or X-ray evidence of necrotizing pneumonia.

Ceftobiprole and ceftriaxone will be administered as follows:

Ceftobiprole will be administered at a dose of 500 mg in 250 mL of 5% dextrose in water (D_5W) every 8 hours at a constant-rate by i.v. infusion over a period of 120 minutes (must not be administered through an i.v. line containing sodium chloride [NaCl] [e.g., saline, Ringer's Lactate solution]).

Ceftriaxone will be administered at a dose of 2 g in 50 mL of D_5W every 24 hours by i.v. infusion over a period of 30 minutes.

Placebo will be used to maintain the blind for both cephalosporins:

- For subjects randomized to ceftobiprole, a 30-minute i.v. infusion of placebo of 50 mL of D_5W will be given every 24 hours concomitantly with ceftobiprole and will begin with the first dose of ceftobiprole.
- For subjects randomized to ceftriaxone, a 120-minute i.v. infusion of placebo of 250 mL of D_5W will be given every 8 hours concomitantly with ceftriaxone and will begin with the first dose of ceftriaxone.

Note: The 30-minute infusion should be administered first (placebo or ceftriaxone) followed by the 120-minute infusion (ceftobiprole or placebo), to ensure that active treatment is delivered to the subject as soon as possible.

Optional oral switch regimen:

- Cefuroxime axetil 500 mg every 12 hours orally.

Linezolid/Placebo Therapy for Ceftriaxone-resistant *S. pneumoniae* and *S. aureus*

Antibiotic coverage of ceftriaxone-resistant *S. pneumoniae* (CRSP):

Linezolid or placebo should be added to study treatment for subjects with confirmed ceftriaxone-resistant *S. pneumoniae* provided the sensitivity of the isolate to linezolid has been confirmed.

Anti-staphylococcal Therapy:

If the incidence of methicillin-resistant *S. aureus* (MRSA) in CAP isolates is prevalent (i.e., greater than 15%) in CAP in the local institution or region, or additional subject-specific risk factors are present, such as a history of prior MRSA infection or a surveillance culture positive for MRSA, linezolid/placebo should be added to cover the possibility of *S. aureus* based on the presence of:

- Gram-stain from adequate sputum with gram-positive cocci in clusters as predominant organism (>10/hpf) or respiratory culture positive for *Staphylococcus* within 72 hours before randomization.

SYNOPSIS (CONTINUED)

In addition, 2 or more of the following criteria:

- Temperature >39°C
- Heart rate >140 per minute
- Respiratory rate >30 breaths per minute
- Hemoptysis
- Leukopenia
- Influenza symptoms within last 2 weeks (cough, coryza, fever)
- Necrotizing lesion(s) on chest X-ray or CT scan
- Known local case(s) of panton-valentine leukocidin (PVL) positive *S. aureus* infections

Linezolid/placebo will be discontinued at 48 hours if *S. aureus* is not isolated from the baseline cultures.

If the subject meets the criteria listed above but a Gram stain cannot be immediately obtained (i.e., the sputum sample is obtained at night), linezolid can be added empirically until the Gram stain results are available. If the results do not show gram-positive cocci in clusters from an adequate specimen, then treatment with linezolid should be discontinued.

If the incidence of MRSA in CAP isolates is not prevalent (i.e., less than 15%) in the local institution or region, the investigator may wait for definitive confirmation of MRSA sensitive to linezolid in the respiratory specimen collected within 72 hours before randomization prior to adding linezolid/placebo.

The following dosage of linezolid/placebo will be administered:

- Linezolid will be administered to those subjects randomized to ceftriaxone, at a dose of 600 mg in 300 mL of D_5W every 12 hours by i.v. infusion over a period of 60 minutes. Placebo of 300 mL of normal saline (NaCl 0.9% solution) will be administered to those subjects randomized to ceftobiprole, at a volume of 300 mL every 12 hours by i.v. infusion over a period of 60 minutes. Normal saline (NaCl 0.9% solution) will replace D_5W as the placebo solution for ceftobiprole in order to minimize the amount of free water delivered to subjects who are randomized to receive additional coverage with linezolid/placebo as follows:

Ceftobiprole Arm	Ceftriaxone Arm
Placebo (50 mL D_5W) every 24 hours	Ceftriaxone (2g in 50 mL D_5W) every 24 hours
Ceftobiprole (500 mg in 250 mL D_5W) every 8 hours	Placebo (250 mL *normal saline*) every 8 hours)
Placebo (300 mL *normal saline*) every 12 hours	Linezolid (600 mg in 300 mL D_5W) every 12 hours

EFFICACY EVALUATIONS/CRITERIA:

The primary endpoint, clinical cure rate, is defined as the ratio of the number of clinically cured subjects to the total number of subjects in the population, at the TOC visit.

The secondary endpoints consist of: microbiological eradication rate at the TOC visit, clinical cure rate in subjects requiring mechanical ventilation within 48 hours of enrollment, microbiological cure rate in subjects requiring mechanical ventilation within 48 hours of enrollment, clinical and microbiological relapse at LFU visit, and 30-day pneumonia-specific mortality rates (all deaths due to pneumonia within 30 days after randomization).

SYNOPSIS (CONTINUED)

At the baseline (Day 1) assessment, the following clinical signs and symptoms will be reported as present or absent: cough, production of purulent sputum or respiratory secretions, presence of rales or evidence of pulmonary consolidation on chest auscultation, rigors or shaking chills, pleuritic chest pain, tachypnea, and hypoxemia.

At all subsequent assessments, changes in signs and symptoms will be reported in comparison with the baseline (Day 1) assessments as: worsened from baseline visit, unchanged from baseline visit, improved from baseline visit, or absent.

At the TOC assessment, the clinical outcome will be rated as follows: Cure, Failure, and Unable to evaluate (see Section 9.3, Efficacy Evaluations for clinical outcome definitions).

At the LFU assessment, the TOC clinical outcome of failure or unable to evaluate will be carried forward as the LFU outcome assessment. Subjects with TOC clinical outcome of cure will be categorized as: Cure, Relapse, and Unable to evaluate.

SAFETY EVALUATIONS*:

Safety will be evaluated by examining the incidence and type of adverse events, and changes in clinical laboratory test results, physical examination results, 12-lead ECGs, and vital sign measurements.

An Independent Data Monitoring Committee (IDMC) will be been established to monitor data on an ongoing basis to ensure the continuing safety and efficacy of subjects enrolled in this study study and efficacy of the study drug. Thereafter, the committee will meet periodically to ensure continued safety and efficacy. After each review, the IDMC will make recommendations regarding the continuation of the study. The details will be provided in a separate IDMC charter.

The IDMC will consist of independent, multidisciplinary experts relevant to the study. The IDMC responsibilities, authorities, and procedures will be documented in its charter. Minutes of IDMC meetings will be provided to the sponsor with comments and recommendations after the completion of the study in keeping with the established charter.

PHARMACOKINETIC EVALUATIONS*:

All subjects will participate in either sparse or rich PK sampling.

Refer to the PK laboratory manual, which will be provided as a separate document, for information regarding handling of biological samples. The exact dates and times of blood sampling must be recorded in either the case report form (CRF) or the laboratory requisition form as appropriate.

At selected sites for rich PK sampling:

Seven blood samples and 1 urine sample (over 8 hours) for each subject will be obtained for PK analysis at selected sites with appropriate facilities and equipment. Approximately 120 subjects (approximately 60 subjects on ceftobiprole treatment and approximately 60 subjects on ceftriaxone with or without linezolid) will participate in PK sample collections. The selected sites may also include some elderly subjects (>65 years) for the PK sampling, if they are available.

* This section of the protocol has been revised. Please refer to the section of this document entitled PROTOCOL AMENDMENTS (Amendment INT-3, 22 December 2006) for a detailed description of the specific changes.

SYNOPSIS (CONTINUED)

On Day 4±1, one 3 mL blood sample will be obtained immediately before the start of the 120-minute morning infusion (predose); subsequent blood samples will be obtained at 15 minutes, 1, 2 (immediately before the end of infusion), 4, 6, and 8 hours after the start of the morning infusion. On this day, subjects will be asked to void and discard urine immediately before the start of the infusion. Subsequently, urine will be collected during the period of 0 to 8 hours after the start of the 120-minute morning infusion. Subjects will be encouraged to drink water ad libitum.

Subjects may refuse rich PK blood sampling and still be enrolled in the study. In this circumstance, sparse PK samples should be collected for these subjects.

At sites for sparse PK sampling:

Three sparse PK blood samples will be collected for each subject who does not participate in the rich PK sampling. On study Day 1, two PK blood samples (3 mL each) will be taken; one at approximately 2 hours (or immediately before the end of the first 120-minute infusion) and the other 6 hours after the start of this infusion (the first dose of the entire treatment). On Day 4±1, another PK blood sample (3 mL) will be taken at any time during the dosing interval, but starting at least 30 minutes after the onset of this 120-minute morning infusion. No PK urine sample will be collected for subjects with sparse sampling.

STATISTICAL METHODS*:

The primary analysis of clinical cure rate will be based on the clinically evaluable (CE) population and the intent-to-treat (ITT) population as coprimary populations.

A noninferiority hypothesis will be assessed based on clinical cure rates at the TOC visit. The hypotheses will be: 1) H0: The clinical cure rate of ceftobiprole is more than 10% inferior to that of the comparator and 2) H1: The clinical cure rate of ceftobiprole is not more than 10% inferior to that of the comparator. This hypothesis will be operationally tested by presenting the 2-sided 95% confidence limits of the between treatment difference in clinical cure rate at the TOC visit. In order to claim noninferiority, the lower bound of the 2-sided 95% confidence interval must be ≥-10%.

The sample size determination is based on a noninferiority test of ceftobiprole versus the comparator in terms of the primary efficacy parameter, clinical cure rate. The assumptions on which the sample size estimate is based are as follows: 1) clinical cure rates of 90% for both randomized study treatments ceftobiprole and the comparator; 2) limit of noninferiority of ceftobiprole versus the comparator is 10%; 3) type 1 error probability (α) = 0.05 (2 sided); 4) type 2 error probability (β) = 0.2 (power = 0.8 = 1 - β); and 5) the evaluable rate is 80%.

Based on these assumptions, 670 subjects will need to be randomized to ensure 532 clinically evaluable subjects, 266 subjects in each treatment group.

* This section of the protocol has been revised. Please refer to the section of this document entitled PROTOCOL AMENDMENTS (Amendment INT-3, 22 December 2006) for a detailed description of the specific changes.

TIME AND EVENTS SCHEDULE*

	Prerandomization Phase	Double-Blind Treatment Phase							Follow-Up Phase	
	Baseline	On-treatment[a,b]								
Study Day	Predose (Day 1)[c]	Day 1	Day 3[b,d,e]	Days 4 to 14 i.v. oral switch (IOS) (optional)[f]	Day 5[b,e,g]	Day 10[b,e,h]	Day 14[b]	End-of-Treatment (EOT) (within 48 hours after the end of therapy)[i]	Test-of-Cure (TOC)/Early Termination (7 to 14 days after last dose of study drug)[j]	Late Follow–Up (LFU) (28 to 35 days after last dose of study drug)[k]
Visit	1	1	2	3	4	5	6	7	8	9
**Screening/Administrative Procedures**										
Informed consent	X									
Inclusion/exclusion criteria	X									
PORT Severity Index (PSI) score	X									
Medical history and demographics	X									
Prestudy therapy (including previous 3 days of antibiotics)	X									
Preplanned surgery/procedures	X									
Urine for *Legionella pneumophila* urine antigen test[l]	X									
Blood sample for mycoplasma and chlamydia titers	X									X
Pregnancy test[m]	X								X	
**Study Drug Administration**										
Randomization	X									
Study drug administration[b,n]		X------	------	------	------	------	------X			
Drug accountability[n]		X------	------	------	------	------	------X	X		
**Safety/Efficacy Procedures**										
Vital signs[o]	X	X	X	X	X	X	X	X	X	X[p]
Physical examination	X			X[q]				X[q]	X[q]	X[p]

* This section of the protocol has been revised. Please refer to the section of this document entitled PROTOCOL AMENDMENTS (Amendment INT-3, 22 December 2006) for a detailed description of the specific changes.

TIME AND EVENTS SCHEDULE (CONTINUED)

	Prerandomization Phase	Double-Blind Treatment Phase							Follow-Up Phase	
	Baseline	On-treatment[a,b]								
**Study Day**	**Predose (Day 1)[c]**	**Day 1**	**Day 3[b,d,e]**	**Days 4 to 14 i.v. oral switch (IOS) (optional)[f]**	**Day 5[b,e,g]**	**Day 10[b,e,h]**	**Day 14[b]**	**End-of-Treatment (EOT) (within 48 hours after the end of therapy)[i]**	**Test-of-Cure (TOC)/Early Termination (7 to 14 days after last dose of study drug)[j]**	**Late Follow–Up (LFU) (28 to 35 days after last dose of study drug)[k]**
**Visit**	**1**	**1**	**2**	**3**	**4**	**5**	**6**	**7**	**8**	**9**
12-lead ECG	X			X					X	
Laboratory tests (hematology/chemistry)	X[r]			X[r]				X	X	X[p]
Urinalysis	X			X				X	X	X[p]
Chest X-ray	X			X[t]				X[t]	X[s]	X[p]
Assessment of signs and symptoms	X		X	X	X	X	X	X	X	X
Respiratory culture & Gram stain[u]	X		X[v]	X[v]	X[v]	X[v]	X[v]	X[v]	X[v]	X[p,v]
Blood culture	X		X[w]	X[x]	X[w]	X[w]	X[w]	X[w]	X[w]	X[p]
Assessment for eligibility for IOS			X[x]	X	X[w]	X[w]	X[w]			
Assessment of need for continued therapy					X[e]	X[e]	X[e]			
Assessment of clinical outcome				X				X	X	X
**Pharmacokinetic (PK) Sampling**										
Rich PK blood sampling[y]				X (Day 4±1)[z]						
PK urine sampling				X (Day 4±1)[aa]						
Sparse PK blood sampling		X[bb]		X (Day 4±1)[cc]						
**Ongoing Subject Review**										
Concomitant medications		X	X	X	X	X	X	X	X	X
Adverse Events	X	X	X	X	X	X	X	X	X	X
Hospitalization status		X	X	X	X	X	X			
Medical Resource Utilization (MRU) data collection								X	X	X

Footnotes are on the following page.

TIME AND EVENTS SCHEDULE (CONTINUED)

[a] While hospitalized, subjects must be seen daily.

[b] The total duration of study drug therapy (intravenous [i.v.] plus oral) for all subjects will be a minimum of 5 days and a target of 7 days. If, in the investigator's opinion, a subject requires additional days of study therapy, the duration of the therapy may be extended (up to a maximum of 10 days). Therapy can be further extended to a maximum of 14 days for subjects with a history of persistent bacteremia or necrotizing pneumonia on X-ray after approval by the sponsor's Medical Monitor. At each on-treatment assessment, the subject should be seen early enough and a decision made concerning the need for additional therapy so that no doses of study drug are missed. A 24-hour window to see the subject is allowable.

[c] The baseline assessments will be performed before dosing on Day 1.

[d] All subjects will be evaluated at Day 3 and all subjects should be assessed daily while hospitalized until eligibility for IOS is achieved (switch is optional).

[e] Determine if additional therapy is required. If so, perform the assessments as noted. If additional therapy is not required, perform the assessments for EOT visit.

[f] At any time after 3 days (72 hours) of i.v. therapy (between Days 4 and 14) unless the subject was bacteremic upon enrollment, if the subject meets the protocol defined criteria (see Section 9.1.4, Criteria for i.v. to Oral Switch) for improvement and hospital discharge is desirable, at the investigator's discretion the subject may be changed to oral medication. Assessments will be performed at the time of switch and before hospital discharge.

[g] At the investigator's discretion, therapy may be stopped anytime after the fifth day of therapy when the infection is considered clinically cured (based on normalization of the clinical signs and symptoms of infection and improvement in the chest X-ray and after a minimum of 5 total treatment days).

[h] If treatment has been extended to 10 days, and it is determined that the subject needs additional antibiotics, the assessments should be performed and any significant findings discussed with the Medical Monitor. If the subject does not need additional antibiotics, the EOT assessment should be performed.

[i] End-of-treatment (EOT) assessments must be performed within 48 hours after the end of therapy.

[j] If a subject withdraws prematurely, TOC/early termination visit procedures must be completed at time of withdrawal.

[k] Late follow-up by telephone contact unless an examination is needed to evaluate relapse (need for further antibiotic treatment) or abnormalities at TOC assessment.

[l] The results of the *legionella* urine antigen test should be available within 24 hours after enrollment. Subjects with proven *legionella* infection should be discontinued from the study and treated appropriately.

[m] Women of childbearing potential only. Pregnancy testing will be performed by serum or urine according to local regulations. Additional serum or urine pregnancy tests will be conducted throughout the study in sufficient number, as determined by the investigator or according to local regulations, to establish the absence of pregnancy during the study.

[n] The pharmacist will be unblinded to prepare appropriate dosing. An unblinded monitor will perform drug accountability.

[o] To include temperature, pulse, respiratory rate, and blood pressure and must be done twice daily at least 8 hours apart, while hospitalized. The vital signs that reflect the highest temperature recorded for the subject on that day should be captured in the case report form (CRF).

[p] To be measured at the discretion of the investigator, (e.g., clinically relevant abnormalities, findings, or values or to evaluate relapse).

[q] Only clinically significant changes in abnormalities from previous assessment should be recorded.

[r] Subjects with serum sodium levels outside of the normal range at baseline or during the trial should have their electrolytes measured on a daily basis or more often as clinically indicated until the serum sodium level is within the normal range.

[s] Chest X-ray is mandatory, demonstrating an improved or stable infiltrate.

[t] Performed only as part of routine subject management.

[u] Repeat within 24 hours if an inadequate specimen is obtained.

[v] If sputum or other respiratory tract specimens are available.

[w] If screening blood cultures were positive, blood culture must be repeated at each visit or more frequently if clinically indicated until negative cultures are obtained.

JNJ-30982081 (BAL5788) (ceftobiprole): Clinical Study 30982081-CAP-3001

TIME AND EVENTS SCHEDULE (CONTINUED)

[x] If blood cultures obtained at the enrollment visit (baseline) are positive for a pathogen, blood cultures should be repeated and must be negative after a minimum of 3 days (72 hours) incubation before change to oral antibiotics.

[y] Will be conducted at selected sites. Site/subjects not participating in the rich PK blood sampling will participate in sparse PK blood sampling.

[z] Pharmacokinetic (PK) blood samples to be drawn at the following time points: predose, 15 minutes, 1, 2 (immediately before the end of infusion), 4, 6, and 8 hours after the start of the morning infusion.

[aa] Pharmacokinetic (PK) urine collection from 0 to 8 hours after the start of the morning infusion (subject is required to void and discard urine immediately before the start of the infusion).

[bb] Pharmacokinetic (PK) blood samples to be drawn at 2 hours (or immediately before the end of the first infusion) and 6 hours after the start of the infusion on Day 1.

[cc] A single PK blood sample to be drawn at any time after 30 minutes of the start of the morning infusion on Day 4 ± 1.

ABBREVIATIONS

NOTE: Abbreviations for PK parameters are defined in Section 9.2.3, Pharmacokinetic Parameters.

ALT	alanine aminotransferase
ANC	absolute neutrophil count
AST	aspartate aminotransferase
AUC	area under the plasma concentration-time curve
BAL	bronchoalveolar lavage
β-hCG	beta-human chorionic gonadotripin
BUN	blood urea nitrogen
CA-MRSA	community-acquired methicillin-resistant *Staphylococcus aureus*
CAP	community-acquired pneumonia
CE	clinically evaluable
CLSI	Clinical and Laboratory Standards Institute
C_{max}	maximum concentration of drug
Cl_{Cr}	creatinine clearance
CRF	case report form
CRSP	ceftriaxone-resistant *Streptococcus pneumoniae*
D_5W	5% dextrose in water
EOT	end-of-treatment
GCP	Good Clinical Practice
ICH	International Conference on Harmonisation
ICU	intensive care unit
IDMC	Independent Data Monitoring Committee
IEC	Independent Ethics Committee
IOS	i.v. to oral switch
IRB	Institutional Review Board
ITT	intent-to-treat
i.v.	intravenous
IVRS	interactive voice response system
LFU	late follow-up

ABBREVIATIONS (CONTINUED)

LPF	low powered field
MIC	minimum inhibitory concentration
mITT	modified Intent-to-Treat
MRSA	methicillin-resistant *Staphylococcus aureus*
MRU	medical resource utilization
NaCl	sodium chloride
PBP	penicillin-binding protein
PK	pharmacokinetic
PORT	Pneumonia Outcomes Research Trial
PRSP	penicillin-resistant *Streptococcus pneumoniae*
PSI	PORT Severity Index
PVL	panton-valentine leukocidin
QTcF	QT interval corrected with Fridericia's formula
spp	species
TOC	test-of-cure
U.K. SmPC	United Kingdom summary of product characteristics
VISA	vancomycin-intermediate *Staphylococcus aureus*
VRSA	vancomycin-resistant *Staphylococcus aureus*

1. INTRODUCTION

Ceftobiprole (BAL9141, Ro-63-9141) is the first extended-spectrum cephalosporin with activity against methicillin-resistant *Staphylococcus aureus* (MRSA) to enter therapeutic trials. Ceftobiprole medocaril (BAL5788, Ro-65-5788) is the water-soluble prodrug suitable for intravenous (i.v.) administration. It has activity against both gram-positive and gram-negative bacterial pathogens. Like other cephalosporins, ceftobiprole is bactericidal. However, in contrast to older cephalosporins, its C-3 substituent is not eliminated during the reaction with penicillin-binding proteins (PBPs), thus allowing a tight drug-protein interaction leading to marked inhibition even of resistant target enzymes. Its enhanced activity against PBP2a, the main resistance determinant in MRSA, results in clinically significant activity against this pathogen. Ceftobiprole is currently under investigation for treatment of skin infections and nosocomial pneumonia.

Cephalosporins (i.e., cefotaxime and ceftriaxone) are often the drugs of choice to treat community-acquired pneumonia (CAP) as they are bactericidal, penetrate into pulmonary tissues, and have a well-known safety profile.[1,2] Consistent with other cephalosporins, ceftobiprole is active against many pathogens associated with CAP such as *Streptococcus pneumoniae* (including penicillin-resistant strains), *Haemophilus influenzae*, *Moraxella catarrhalis*, and *Klebsiella pneumoniae*. In addition, ceftobiprole demonstrates in vitro microbiological activity against ceftriaxone-resistant strains of *S. pneumoniae*.[3] Therefore, it is appropriate to study ceftobiprole for the treatment of CAP.

For more detailed information, refer to the Investigator's Brochure for ceftobiprole.[4]

The term sponsor used throughout this document refers to the entities listed in the Contact Information page(s), which will be provided as a separate document.

1.1. Background

1.1.1. Ceftobiprole

Nonclinical Studies

Microbiologic Profile

Ceftobiprole has demonstrated potent in vitro activity against many gram-positive and gram-negative bacterial pathogens. Due to its unusually specific inhibition of the resistance enzyme PBP2a, ceftobiprole is active against multi-resistant staphylococci including MRSA (the mean inhibitory concentration of ceftobiprole at which 90% of tested strains are susceptible [MIC_{90}] is 2 μg/mL). Ceftobiprole displayed little or no resistance development in vitro against pathogens such as MRSA and drug-resistant pneumococci.[5,6] In an endocarditis model of rat infected with multi-resistant staphylococci, ceftobiprole was superior to vancomycin.[7] Ceftobiprole exhibited potent in vitro and in vivo activity against penicillin-resistant pneumococci and strains of *Enterococcus faecalis* as well as exhibiting useful in vitro activity against most of the common gram–negative pathogens including *Escherichia coli, K. pneumoniae, Proteus mirabilis* (but not *Proteus vulgaris), Morganella morganii*, *Providencia* species (spp)*, Citrobacter* spp*, Enterobacter* spp, many ceftazidime-susceptible *Pseudomonas aeruginosa* and some strains of *Acinetobacter* spp; Ceftobiprole is not stable to the extended spectrum β−lactamases (ESBLs), has no activity against *Enterococcus faecium* and no useful activity against *Bacteroides* spp. Ceftobiprole is active, however, against *H. influenzae*, *Neisseria gonorrhoeae, M. catarrhalis,* and *Vibrionaceae*.

Compared to third generation cephalosporins, ceftobiprole has enhanced activity against multi-drug-resistant gram-positive pathogens such as MRSA and penicillin-resistant *S. pneumoniae* (PRSP),[8] including strains that are ceftriaxone-resistant.[3]

Animal Pharmacokinetic and Toxicology Data

After i.v. administration, ceftobiprole medocaril was rapidly converted in vivo to ceftobiprole in all animal species studied. Ceftobiprole had a volume of distribution equal to the extracellular compartment, and was eliminated as unchanged drug in urine by passive glomerular filtration. Excretion was complete, without retention of drug-related material.

Relevant animal species were selected for the toxicological investigation. Ceftobiprole doses up to 175 mg/kg given twice daily for 2 weeks or 250 mg/kg once daily for 4 weeks were well tolerated in rats, and doses up to 100 mg/kg given twice daily for 2 weeks or once daily for 4 weeks were well tolerated in marmosets. Local tolerance at the site of infusion was good. In all these studies, animals were exposed in a dose proportional manner to ceftobiprole at plasma and urinary levels significantly higher than the human exposure. There was also no indication of accumulation, time-dependent changes in exposure, or sex differences in exposure to ceftobiprole. Main findings at high doses included renal toxicity associated with drug precipitation in the distal tubules (related to low solubility of ceftobiprole), occasional vomiting in marmosets, and central nervous system (CNS) toxicity at very high doses. In conscious dogs, significant vomiting was noted at doses ≥50 mg/kg after short (30 minutes) infusions, but could be attenuated by extension of the infusion time to 2 hours. Ceftobiprole was non-nephrotoxic in rabbits, nonteratogenic and nonembryotoxic in rats and cynomolgus monkeys, and had no adverse effect on the fertility of rats. Ceftobiprole was not mutagenic, phototoxic or skin sensitizing, and had no relevant effect on cardiovascular function in rats, marmosets, and conscious dogs. Its convulsive potential in mice was similar to imipenem, another β-lactam antibiotic with a defined seizure potential in humans. Please refer to the Investigator's Brochure for a complete description of available toxicology data.[4]

Clinical Studies

Human Pharmacokinetics

A total of 133 subjects have received ceftobiprole in 8 Phase 1 studies. Ceftobiprole medocaril was very rapidly converted to the active drug ceftobiprole. The plasma concentration-time profiles of ceftobiprole at a given dose level were comparable from one study to the other, with a low variability among subjects and among studies. In these studies, the half-life of ceftobiprole was approximately 3 to 4 hours.

After single and repeated administration, pharmacokinetic (PK) parameters (maximum plasma concentration [C_{max}] and area under the plasma concentration-time curve [AUC]) of ceftobiprole were dose proportional from 125 to 1,000 mg.

After repeated administration (up to 12 days), the PK properties of ceftobiprole were time independent; no decrease in exposure was observed to indicate induction, and the observed accumulation factor (1.02 to 1.06) with twice-daily dosing over a 0.5-hour infusion was equal to the predicted value of 1.1, calculated by the terminal half-life ($t_{1/2}$) (assuming 4 hours of $t_{1/2}$), indicating negligible accumulation.

The main route of elimination of ceftobiprole was urinary with mean urinary recoveries of 53% to 96% in various dose groups in healthy subjects. The highest individual peak urinary concentration for a dose of 500 mg (given as a 30-minute infusion) was approximately 2 to 12 times lower than the concentration at the no observed effect dose level in rat toxicological studies. In a study conducted in subjects with renal dysfunction, the systemic clearance of ceftobiprole correlated well with creatinine clearance (Cl_{Cr}), so that desired drug levels can be maintained in subjects with renal dysfunction by dose adjustments based on Cl_{Cr}.

In a study conducted in men versus women, systemic exposure to ceftobiprole was approximately 15% higher in women compared with men and was primarily related to the lower body weight. When PK parameters were corrected for body weight, no sex differences were apparent; therefore, dose adjustment is not required in women.

Safety and Tolerability in Healthy Subjects

Ceftobiprole was well tolerated in 133 subjects who received ceftobiprole in 8 Phase 1 studies. A total of 33, 63, and 12 subjects received one or more doses of 500, 750, and 1,000 mg, respectively. Of these, 24 (22%) subjects were exposed to more than a single dose. No serious adverse events occurred in any of these studies. No subject was withdrawn from treatment due to adverse events. The most common drug-related adverse events were nausea, vomiting, disturbance of taste, flatulence, headache, and fatigue. Other temporary effects observed at different dosing regimens of ceftobiprole than used in the current study include sleepiness and malaise, restlessness, feeling hot or cold, dizziness, sore throat, lower abdominal pain, diarrhea, indigestion, skin sensations, fainting, tiredness, swelling in the extremities, itching, low blood pressure, abnormal urine color or urine sediment, restless leg syndrome, toothache, insomnia (difficulty falling asleep or staying asleep), itchy rash, increased sweating, abscess (localized collection of pus

surrounded by inflamed tissue), anxiety, decreased heart rate, and a mild reaction at the injection site. No clinically relevant findings were reported for vital signs (blood pressure, body temperature, and pulse).

A mild and temporary effect on liver transaminases has been reported, but this was thought to be unconnected to the ceftobiprole treatment and occurred at a different dosing regimen than used in the current study. In a few cases, eosinophilia has been reported after treatment with ceftobiprole. Other reported laboratory abnormalities observed at different dosing regimens of ceftobiprole than used in the current study include anemia, increased serum creatine, thrombocytopenia, increased international normalized ratio (INR), hypomagnesemia, hypo- and hyper-kalemia and hypoglycemia.

As part of the routine assessment in the Phase 1 studies, the effect of ceftobiprole on QTc was screened. A small, multiple-dose, Phase 1 study (BAP000393: 6 placebo, 6 treated) of 1,000 mg of ceftobiprole infused over 90 minutes, 3-times-a-day demonstrated a QT interval corrected with Fridericia's formula (QTcF) compared with baseline for subjects receiving ceftobiprole of 1 msec on Day 1 and 3 msec on Day 8. For subjects receiving placebo there was a decrease in QTcF of 9 msec on Day 1 and 15 msec on Day 8 compared with baseline. Correction of QT for heart rate using linear regression revealed no clinically or statistically significant difference of QTc between ceftobiprole and placebo-treated subjects. Pooled analysis of data from 79 subjects in the Phase 1 studies (BAP00010, BAP00034, BAP00036, and BAP00058) revealed no clinically significant prolongation in QTcF across the studies in doses ranging from 125 to 1,000 mg. In these studies, there was also no dose effect on QTcF, and no correlation of QTcF with C_{max} after single or repeat dosing.

Renal function and urine parameters did not provide any evidence of renal toxicity; no drug precipitation was detected in urine sediment. No subjects in the Phase 1 program demonstrated signs of acute tubular necrosis or interstitial nephritis, the 2 renal toxicities that have been previously associated with cephalosporins.

Phase 2 Study of Ceftobiprole in Complicated Skin and Skin Structure Infections

Forty hospitalized subjects with complicated skin and skin structure infections were treated with 30-minute i.v. infusions of 750 mg ceftobiprole medocaril, twice daily for 7 to 14 days.

All 40 subjects completed at least 7 days of therapy. Six subjects could not be clinically evaluated, 4 because of antibiotic treatments for other conditions, one subject had a skin carcinoma (infected site was removed), and one subject was withdrawn for an adverse event (suspected gout attack).

The primary efficacy variable was clinical cure rate, assessed at a test-of-cure (TOC) evaluation 7 to 10 days after the end of therapy. All 34 clinically evaluable subjects were clinically cured at TOC. The investigators also reported clinical cures for the 6 subjects who were judged to be nonevaluable for clinical efficacy. One clinical relapse was reported and attributed to an underlying osteomyelitis that had not been previously diagnosed.

The microbiological eradication rate was 91% among 23 (58% of the study population) evaluable subjects. All 4 infections caused by MRSA were reported as clinical cures at TOC, with microbiological eradication in 3 cases and 1 case of persistent colonization with no need of further antibiotic treatment.

The most frequently reported related adverse events were vomiting (30%), nausea (28%), insomnia (23%), eosinophilia (18%), elevated blood triglycerides (18%), hypomagnesemia (15%), and anxiety (13%). Most reported adverse events were mild to moderate in severity. Serious adverse events were reported for 5 subjects. Two subjects experienced treatment emergent adverse events of severe or life-threatening intensity (severe polyarthritis; severe acute respiratory failure and life–threatening respiratory arrest). One nontreatment-emergent adverse event, which may have been attributable to a pre–existing condition that was undiagnosed at the time of study enrollment, also had severe intensity (osteomyelitis); this event was considered serious. The other 4 serious adverse events were acute allergic reaction with signs of skin rash, face edema, and oropharyngeal swelling without shortness of breath (resolved); respiratory arrest with pinpoint pupils and a positive urine test for opiates (responded to naloxone); polyarthralgia

with raised inflammatory markers in a subject with a history of chronic polyarthritis and gout (resolved); and central line infection requiring broad-spectrum treatment with antibacterials and antifungals (resolved).

The PK properties of ceftobiprole and its prodrug in subjects with skin infections were similar to those observed in previous studies with healthy subjects.

1.1.2. Comparator, Ancillary, and Oral Study Drugs

Comparator Study Drugs

In this study, ceftobiprole will be compared with i.v. ceftriaxone with/without linezolid (comparator).

Ceftriaxone is a cephalosporin antibiotic that has been shown to be safe and efficacious in treating CAP and has good activity against the major bacterial pathogens including *S. pneumoniae*, *H. influenzae*, *S. aureus* (but not MRSA), and *K. pneumoniae.*[9] Ceftriaxone is recommended in treatment guidelines for treating hospitalized patients with pneumonia.[1,2] The most common side effects (incidence ≥1%) of ceftriaxone are hematology disturbances (eosinophilia, thrombocytosis, and leukopenia), elevations of liver transaminases, diarrhea, rash, elevations of blood urea nitrogen (BUN), and irritation at the site of injection. Some less common side effects (incidence <1%) of ceftriaxone are phlebitis at the i.v. site, pruritus, fever, chills, anemia (including hemolytic), neutropenia, thrombocytopenia, prolongation of prothrombin time, elevations of alkaline phosphatase and bilirubin, elevation of serum creatinine, urinary casts, headache, dizziness, vaginitis, diaphoresis, and flushing. Rare but serious side effects include anaphylaxis, seizures, renal failure, agranulocytosis, and pseudomembranous colitis.

Linezolid is an oxazolidinone antibiotic that has been shown to be safe and efficacious in treating CAP due to gram-positive pathogens including MRSA and PRSP.[10] The most common side effects (incidence ≥1%) of linezolid are diarrhea, headache, nausea, vomiting, insomnia, constipation, rash, dizziness, and fever. Some less common side effects (incidence <1%) of linezolid include oral moniliasis, vaginal moniliasis, hypertension, dyspepsia, localized abdominal pain, pruritus, and tongue discoloration. Rare

but serious side effects include myelosuppression, optic or peripheral neuropathy, and pseudomembranous colitis.

Subjects who meet the criteria in Section 9.1.4, Criteria for Intravenous to Oral Switch, may (at the investigator's discretion) be switched to cefuroxime axetil. Cefuroxime axetil is an oral cephalosporin that has been shown to be safe and efficacious in treating CAP and has good activity against the major bacterial pathogens including *S. pneumoniae*, *H. influenzae*, *S. aureus* (but not MRSA), and *K. pneumoniae*.[11] Cefuroxime is recommended in treatment guidelines for treating hospitalized patients with pneumonia.[1,2] The most common side effects (incidence ≥1%) of cefuroxime axetil are diarrhea, nausea, vomiting, and transient elevations in liver transaminases. Some less common side effects (incidence <1%) of cefuroxime axetil include rash, abdominal pain and headache. Rare but serious side effects include allergic reactions (anaphylaxis and Stevens-Johnson Syndrome) and pseudomembranous colitis.

1.2. Overall Rationale for the Study

Despite the availability of effective antimicrobial agents, CAP remains a potentially life-threatening disease. Although most patients with CAP are treated as outpatients, approximately 20% of patients with CAP are hospitalized (18% on medical wards and 2% in intensive care units [ICUs]).[12] The Pneumonia Outcomes Research Trial (PORT) developed a clinical prediction rule that quantifies short-term mortality risk for subjects with CAP.[13] The PORT Severity Index (PSI) was validated in 2 independent samples and had a high ability to discriminate between those patients who were at a high risk of dying and those who were not. Thus, the PSI will be used to stratify subjects based on initial pneumonia severity.

The overall mortality rate among patients requiring hospitalization for CAP ranges from 5% to 25% and rises to 20% to 50% in those with severe CAP requiring treatment in the ICU.[12,14,15] Bacteremia is a potential complication in patients with CAP, especially those who are hospitalized, and raises the mortality rate approximately 3-fold.[16,17] Thus, it is especially important to treat CAP patients who require hospitalization with an antimicrobial agent that achieves effective concentrations in plasma and pulmonary tissues and is bactericidal against the most common etiologic agents. *Streptococcus pneumoniae* is the most common etiologic agent isolated from hospitalized

patients with CAP, especially those with severe disease.[18,19] Other less common causative agents include *S. aureus*, *L. pneumophila*, *H. influenzae* and the enteric gram-negative bacilli.[12] Although *S. aureus* is not a common cause of CAP in otherwise healthy patients, this pathogen can be an important cause of pneumonia in patients with diabetes, patients who abuse i.v. drugs, patients with renal failure, and patients who develop secondary bacterial infections during influenza epidemics. Pneumonia due to *S. aureus* tends to be a more severe infection and frequently requires hospitalization.[20,21]

Recently a strain of MRSA has occurred with increasing frequency in the community that differs from hospital-acquired MRSA in several ways including susceptibility to antibiotics and presence of virulence factors (such as the Panton-Valentine leukocidin [PVL]).[22-24] These community-acquired MRSA (CA-MRSA) strains have been associated with necrotizing skin and pulmonary infections,[25-32] especially following influenza infection.[33] The combination of a new strain of influenza causing infection in large numbers of patients plus the increasing prevalence of CA-MRSA, raises the concern that the incidence of CAP due to CA-MRSA may increase over the next few years. Signs and symptoms often present in CA-MRSA typically include high fevers (temperature >39°C), tachycardia (heart rate >140 beats per minute), tachypnea (respiratory rate >30 breaths per minute), hemoptysis, leukopenia, a history of influenza, or necrotizing lesion(s) on chest X-ray or CT scan.[25-32] Due to the high mortality rate of pneumonia due to *S. aureus,* linezolid will be part of the comparator regimen when subjects present with signs and symptoms suggestive of staphylococcal infections.

Beta-lactam antibiotics (penicillins and cephalosporins) have been the mainstay of treatment for CAP for decades. They have a well-known safety profile and penetrate well into the pulmonary tissues. Two cephalosporins, cefotaxime and ceftriaxone, are recommended as first-line treatment for patients with hospitalized CAP in a variety of practice guidelines.[1,2] However, the emergence of *S. pneumoniae* and *S. aureus* strains resistant to the older β-lactams and the increased incidence of these resistant strains in the community setting has raised concern about the utility of empiric treatment of severe CAP with a single, traditional beta-lactam agent. Alternative therapy for resistant gram-positive pathogens, vancomycin and linezolid, are not as rapidly bactericidal as the beta-lactams.

Ceftobiprole is a new cephalosporin with good in vitro activity against PRSP and MRSA, including strains of *S. aureus* with decreased susceptibility to glycopeptides (glycopeptide-intermediate *S. aureus* [GISA] or vancomycin-intermediate *S. aureus* [VISA], and glycopeptide-resistant *S. aureus* [GRSA] or vancomycin-resistant *S. aureus* [VRSA]). Ceftobiprole also has in vitro activity against many enteric gram-negative rods and *H. influenzae.* Recent investigation suggests that ceftobiprole maintains activity with minimum inhibitory concentrations (MICs) ≤1 μg/mL against ceftriaxone-resistant strains of *S. pneumoniae.*[3]

2. OBJECTIVES

The primary objective is to demonstrate the noninferiority of ceftobiprole medocaril compared with ceftriaxone with/without linezolid (comparator) with respect to the clinical cure rate in subjects hospitalized with CAP at the TOC visit.

The secondary objectives are:

- To compare the microbiological eradication rate following treatment with ceftobiprole versus the comparator in subjects with hospitalized CAP at the TOC visit
- To compare the clinical cure rate following treatment with ceftobiprole versus the comparator of hospitalized CAP subjects that require mechanical ventilation within the first 48 hours of enrollment
- To compare the microbiological eradication rate following treatment with ceftobiprole versus the comparator of hospitalized CAP subjects that require mechanical ventilation within the first 48 hours of enrollment
- To compare the clinical and microbiological relapse rates following treatment with ceftobiprole versus the comparator in subjects with hospitalized CAP at the late follow-up (LFU) visit

To compare the 30-day pneumonia-specific mortality rates following treatment with ceftobiprole or the comparator of hospitalized CAP subjects

Other objectives are:

- To assess the PKs of ceftobiprole in subjects treated with ceftobiprole
- To collect medical resource utilization (MRU) data that may be used in future economic modeling

Safety/tolerability objectives are:

- To characterize the safety and tolerability of treatment with ceftobiprole in subjects hospitalized with CAP

Hypotheses

Efficacy:

The clinical cure rate, with respect to treatment with ceftobiprole with/without switch to oral therapy, will be noninferior to a regimen of the comparator, with/without a switch to oral therapy, in the clinically evaluable subjects hospitalized with CAP.

Safety and tolerability:

The safety and tolerability of treatment with ceftobiprole will be similar to a regimen of the comparator in hospitalized CAP subjects.

3. OVERVIEW OF STUDY DESIGN

Approximately 670 subjects, 18 years of age or older, with CAP severe enough to require hospitalization (PSI ≤90 versus PSI≥91) and treatment with i.v. antibiotics for at least 3 days (72 hours), will participate in this study to achieve 532 clinically evaluable subjects (266 in the ceftobiprole group and 266 in the comparator group). Subjects who withdraw from the study will not be replaced.

3.1. Study Design*

This randomized, double-blind, comparator-controlled, multicenter study is designed to assess the efficacy and safety of ceftobiprole compared with the comparator in subjects 18 years of age or older with CAP of sufficient severity to require hospitalization and treatment with i.v. antibiotics for a minimum of 3 days (72 hours). The study will consist of a prerandomization phase (baseline), a double-blind treatment phase with an end-of-treatment (EOT) visit (within 48 hours after the end of therapy), and a follow-up phase with a TOC/early termination visit 7 to 14 days after the last dose of study drug, and a LFU visit 28 to 35 days after the last dose of study drug. The total duration of the study participation for a subject will be up to 49 days.

* This section of the protocol has been revised. Please refer to the section of this document entitled PROTOCOL AMENDMENTS (Amendment INT-3, 22 December 2006) for a detailed description of the specific changes.

Approximately 670 subjects will be randomized to achieve 266 clinically evaluable subjects receiving ceftobiprole and 266 clinically evaluable subjects receiving the comparator. This trial will be conducted in a manner to ensure that investigators, those administering study drug, and subjects are blinded to treatment regimen. Placebo will be used to maintain the blind for both the cephalosporin (ceftobiprole and ceftriaxone) and linezolid therapies. Central randomization will be used to randomly assign subjects in a 1:1 ratio to receive ceftobiprole or the comparator. Subjects will be stratified by the Pneumonia Outcomes Research Trial (PORT) Severity Index (PSI) score at randomization (PSI ≤90 versus PSI ≥91; see Attachment 1, PORT Severity Index [PSI]). Subjects will also be stratified by the need for anti-staphylococcal therapy (placebo or linezolid) based on signs, symptoms, and medical history at enrollment.

All subjects should receive a minimum of 3 days (72 hours) of the i.v. study drug(s) and should continue to receive i.v. therapy while hospitalized (see Section 6, Dosage and Administration). At the discretion of the investigator, subjects may receive study drug infusions as outpatients or through a home-care agency, provided they meet all other protocol requirements, but all i.v. study drug infusions must be administered and monitored by a qualified nurse, a qualified member of the study staff, or an infusion specialist.

Clinical evaluations will include assessment of signs and symptoms of pneumonia and results of laboratory tests and chest X-rays and occur according to the Time and Event Schedule. Clinical outcome will be assessed at the TOC and LFU visits, as well as at the time of optional i.v. to oral switch (IOS).

Microbiological assessments will include cultures of pulmonary secretions and blood, as well as urine antigen test for *L. pneumophila*.[34,35] Pulmonary secretions can be obtained by induction of sputum, bronchoalveolar lavage (BAL), or needle aspirate. Selection and identification of pathogens at each local laboratory will be conducted according to local procedures. Each pathogen isolated will be tested for susceptibility to study antibiotics at the local laboratory and will also be submitted to a central laboratory for further testing.

Rich PK sampling will be done at selected sites in approximately 120 subjects (60 subjects from each treatment group). For all other subjects who do not participate in the rich PK sampling, sparse PK sampling will be conducted.

Safety will be assessed by physical examination, vital signs, adverse events, ECGs, and laboratory tests.

Medical resource utilization data will be collected including resource use data associated with hospitalization, outpatient treatment (other than study-related procedures), and concomitant therapies.

In addition to routine monitoring of ongoing safety data during the study, an Independent Data Monitoring Committee (IDMC) will be used to assess clinical benefit including safety and efficacy during the trial. The IDMC will review unblinded safety and efficacy data at regular time intervals during the study (see Section 9.5, Safety Evaluations).

The following figure presents a schematic overview of the overall study design.

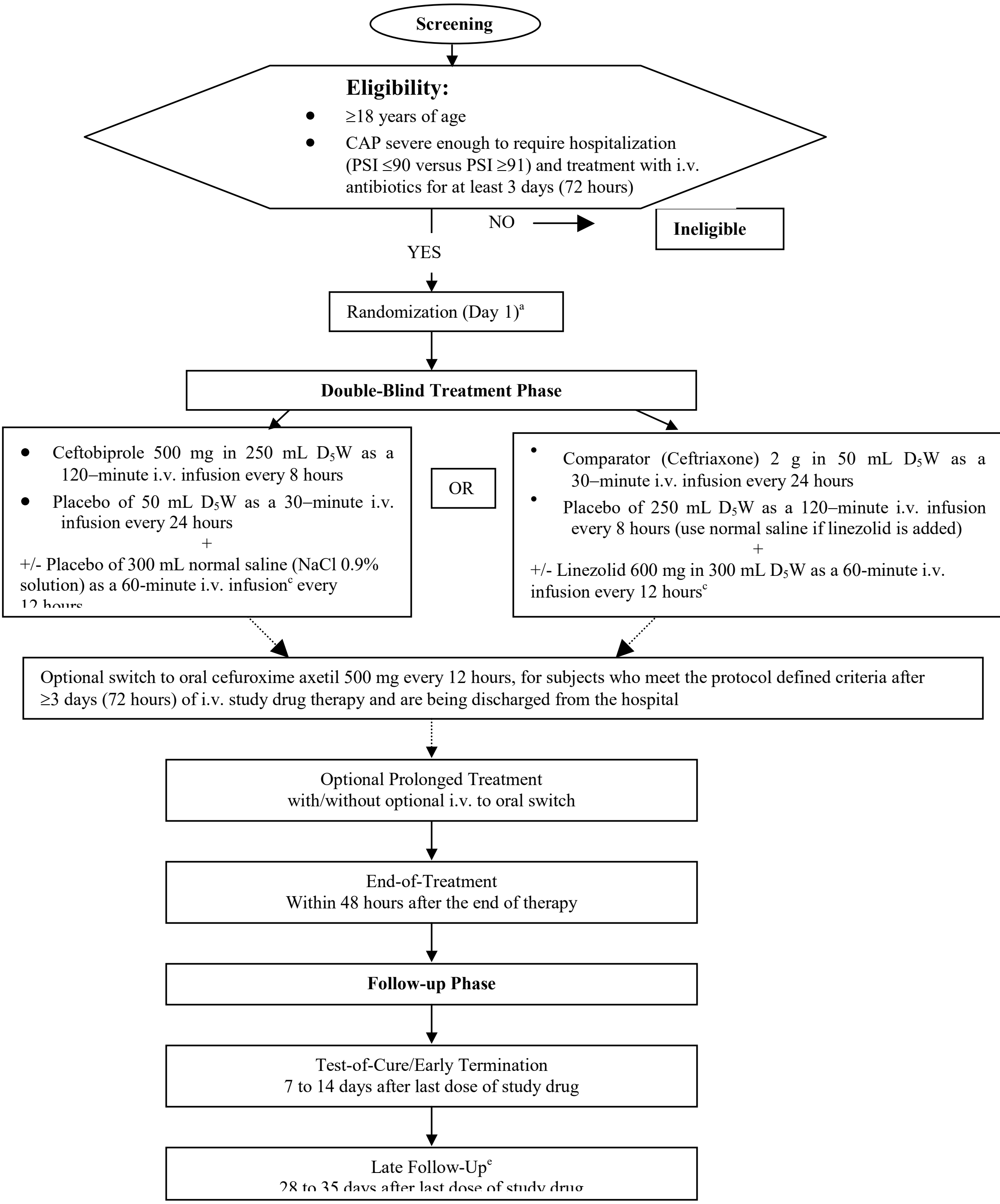

Day of the first infusion. Baseline assessments will be performed before first infusion.
The 30-minute infusion should be administered first (placebo or ceftriaxone), followed by the 120-minute infusion (ceftobiprole or placebo).
arget duration of study drug treatment (i.v. and oral) for all subjects will be a minimum of 5 days and a target of 7 days. If, in the investigato
opinion, a subject requires additional days of study therapy, the duration of the therapy may be extended (up to a maximum of 10 days). Sub
be evaluated at Day 5 and 10 while on therapy.
Linezolid or placebo will be added as concomitant therapy based on initial signs and symptoms suggestive of MRSA or *S. aureus*. Linezolid
placebo should be discontinued once infection with MRSA or CRSP is ruled out.
Therapy can be further extended to a maximum of 14 days after approval by the sponsor's Medical Monitor. Subjects will be evaluated at
Day 14 while on prolonged therapy.
Late Follow-up Visit is by telephone contact. Subjects reporting signs or symptoms consistent with pulmonary infection should be evaluated
person.

3.2. Study Design Rationale*

Rationale for Dose Selection

The ceftobiprole dose selected for this study is 500 mg as a 120-minute infusion every 8 hours.

In experimental studies, the efficacy of ceftobiprole was directly related to the percentage of the dosing interval during which the concentration of ceftobiprole in plasma was above the MIC of target pathogens (T>MIC). This is typical for beta-lactam antibiotics where, in most cases, a T>MIC of 30% to 50% (depending on the pathogen) of the dosing interval is predictive of adequate clinical efficacy in humans. In most cases the enteric gram-negative pathogens (e.g., *E. coli*, *K. pneumoniae*) require a higher T>MIC than do the gram-positive pathogens (e.g., *S. pneumoniae*, *S. aureus*).

The MIC_{90} is 1 to 2 µg/mL for strains of MRSA from Europe, Japan, and the United States. The MIC_{90} of ceftobiprole for *S. pneumoniae* strains ranges from 0.016 µg/mL (penicillin-susceptible strains) to 1.0 µg/mL (penicillin-resistant strains). Extensive Monte Carlo simulations based on PKs in healthy subjects have been performed. The probability of attaining 30% to 60% T>MIC for ceftobiprole MICs ranging from <0.12 to 4 µg/mL with the dosing regimen of 500 mg infused over 120-minutes every 8 hours in subjects with normal renal function is shown in the table below.[36]

Probability of target attainment based on Monte Carlo simulation

% T>MIC	**<0.12** µg/mL	**0.25** µg/mL	**0.50** µg/mL	**1.00** µg/mL	**2.00** µg/mL	**4.00** µg/mL
30	1	0.9998	0.9998	0.9976	0.991399	0.959296
40	0.9999	0.9996	0.9991	0.994899	0.980298	0.880988
50	0.9989	0.9977	0.9953	0.984798	0.947695	0.768477
60	0.9965	0.992099	0.984498	0.961896	0.89729	0.654165

* This section of the protocol has been revised. Please refer to the section of this document entitled PROTOCOL AMENDMENTS (Amendment INT-3, 22 December 2006) for a detailed description of the specific changes.

These analyses indicate that exceeding T>MIC targets of 30% for key pathogens that cause CAP, including *S. pneumoniae* and *S. aureus*, are highly likely (>99%). Furthermore, these analyses indicate that the dose regimen that will be used in this study, will provide sufficient T>MIC (30%) to predict good activity against gram-positive pathogens with MICs as high as 4 μg/mL and is likely to provide sufficient T>MICs for periods as long as 60% of the dosing interval for gram-negative pathogens with MICs ≤2 μg/mL.

For detailed information about PKs and pharmacodynamics of ceftobiprole, refer to the Investigator's Brochure.[4]

Ceftobiprole is available as an i.v. formulation only. No oral formulation has been tested in humans. Intravenous drug treatments must be administered and monitored by a qualified nurse, a member of the study staff, or infusion specialist. There are no controlled studies to assess the appropriate duration of treatment for pneumonia. In most cases, the decision of how long to treat is based on the pathogen, response of the subject to therapy, comorbid conditions, and any complications of pneumonia. Subjects with uncomplicated pneumonia due to *S. pneumoniae* are often treated until they are afebrile for 3 days (72 hours). Subjects with more severe disease and those with necrotizing pathogens (e.g., *S. aureus*, *Klebsiella* spp) are usually treated longer.[1] In this protocol, the treatment duration of 5 to 10 days with the option of further prolongation to 14 days, if needed, will allow for adequate treatment of subjects with a variety of pathogens and conditions.

Rationale for Choice of Comparators and Oral Switch Therapy

Ceftriaxone was chosen as the primary comparator in this study because it is a standard first-line therapy for subjects with CAP infections who require hospitalization.[1,25] Ceftriaxone also has the same route of administration (i.v.) as ceftobiprole.

In order to allow subjects at-risk for MRSA to participate in the study, an additional drug with anti-MRSA activity will be added to ceftriaxone in subjects who meet specific inclusion criteria. Vancomycin, a glycopeptide, is effective against most strains of MRSA, but there are some strains of *S. aureus* with low-level resistance to vancomycin (VISA), and recently strains have been identified with high-level resistance to vancomycin

(VRSA). Linezolid, an oxazolidinone, has a narrow spectrum of activity with no useful gram-negative activity. It has been proven to be effective in treating MRSA infections and has in vitro activity against MRSA strains resistant to vancomycin.[10] A recent analysis has suggested that linezolid may be superior to glycopeptides for the treatment of *S. aureus* pneumonia[37] and it was therefore, chosen as part of the comparator regimen.

Atypical pathogens that cause CAP include *Chlamydophila pneumoniae, Mycoplasma pneumoniae, and Legionella pneumophila.* These pathogens are estimated to cause from 6% to greater than 40% of CAP, with regional and age-related differences.[38,39] However, there is no consensus as to whether these are the primary causative organisms in all cases, and the impact of treatment of these pathogens on patient outcomes remains unclear. *M. pneumoniae* is the most common atypical pathogen, and generally, the onset of disease caused by this pathogen is insidious over several days.[40] A dry hacking cough that is worse at night is common. Community-acquired pneumonia caused by *M. pneumoniae* is usually mild and self-limiting, although occasionally it can cause severe disease, particularly in the elderly. *Chlamydophila pneumoniae* is likely to occur in older people with comorbid conditions or in younger patients with significant comorbid diseases.[40] Sore throat, headache, and non-productive cough are common. Often patients have been sick for more than a few days. Untreated, symptoms can persist for months. Generally, pneumonia caused by either *C. pneumoniae* or *M. pneumoniae* can be differentiated from pneumonia caused by a typical bacteria because it is acute in onset with shaking chills, cough productive of purulent sputum, fever, and an elevated white count. Additionally, atypical pneumonias typically have chest X-ray findings that are bilateral and diffuse. Importantly, a gram stain that does not show organisms but has abundant white cells is consistent with an atypical pneumonia. Therefore, gram stain results, chest X-ray findings, and clinical features will be evaluated in concert to exclude subjects with atypical pneumonias.

In addition to the above clinical assessments, serum for mycoplasma IgG and IgM and chlamydia IgG, IgM, and IgA will be collected at the predose visit and at LFU (if the subject necessitates a visit), to be analyzed at the central laboratory. If a single abnormal mycoplasma IgM titer result of ≥1:28 or a single chlamydia IgM titer of ≥1:10 is obtained at the predose visit, suggestive of an acute (active) infection, the subject may be discontinued

from the study and treated with an agent with activity against the identified atypical pathogen.

As neither of the drug regimens in this protocol are known to be effective against *Legionella* spp, subjects suspected of pneumonia due to *Legionella* should not be enrolled. The urinary antigen assay for *L. pneumophila* serogroup 1 is not technically demanding, and reliably and rapidly detects up to 80% to 95% of community-acquired cases of Legionnaires disease.[41] *Legionella pneumophila* has been implicated in 0.5% to 6% of CAP cases in recent hospital-based series[42,43] and mortality rates ranged from 5% to 25% in immunocompetent hosts.[43] In one series, *L. pneumophila* was a major respiratory tract pathogen in subjects with CAP who required admission to the ICU.[43] *Legionella* urine antigen will be performed in all subjects. It is anticipated that the results of the *legionella* urine antigen test will be available within 24 hours after enrollment. Subjects with proven *legionella* infection should be discontinued from the study and treated appropriately.

It is commonplace, especially for subjects with PSI scores ≤90, to transition from inpatient i.v. therapy to outpatient therapy in the course of treatment for CAP.[44-46] Recognizing that many subjects in the trial will not require hospitalization for the entire duration of antibiotic therapy, to facilitate discharge from the hospital, a switch from i.v. study drugs to oral cefuroxime axetil is allowed after a minimum of 3 days of i.v. therapy, but only for those subjects who meet protocol-specified criteria (see Section 9.1.4, Criteria for Intravenous to Oral Switch) for improvement and are candidates for hospital discharge. Use of cefuroxime axetil is consistent with the recommendation of current practice guidelines.[1,2]

Rationale for PK Assessments

In order to develop a PK structure model and evaluate the dependence of the PK of the active ceftobiprole on subject population covariates, collection of PK samples with rich sampling in a limited number of subjects and with sparse sampling in additional subjects are needed.

The combination of rich PK sampling and sparse PK sampling will be utilized to explore the effects of age, sex, weight, and renal function on plasma ceftobiprole concentrations.

4. STUDY POPULATION

4.1. General Considerations

Approximately 670 subjects will be randomized to achieve 532 clinically evaluable subjects, 266 subjects in each group.

The specific inclusion and exclusion criteria for enrolling subjects in this study are described in the following sections.

4.2. Inclusion Criteria

Subjects must satisfy the following criteria to be enrolled in the study:

- Men or women, 18 years of age or older
- Women must be postmenopausal (for at least 1 year), surgically sterile (have had a hysterectomy or bilateral oophorectomy, tubal ligation, or otherwise be incapable of pregnancy), abstinent, or, if sexually active, be practicing an effective method of birth control (e.g., prescription oral contraceptives, contraceptive injections, intrauterine device, double-barrier method [e.g., condoms, diaphragm, or cervical cap with spermicidal foam, cream, or gel], contraceptive patch, male partner sterilization) before entry and continue to use the same method of contraception throughout the study
- Women must have a negative serum beta-human chorionic gonadotropin (β-hCG) or urine pregnancy test (depending on local regulations) before enrollment
- Willing to adhere to the prohibitions and restrictions specified in this protocol
- Subjects (or their legally acceptable representatives) must have signed an informed consent document indicating that they understand the purpose of and procedures required for the study and are willing to participate in the study

Disease Specific

Subjects with CAP severe enough to require hospitalization and treatment with i.v. antibiotics for at least 3 days (72 hours) defined as follows:

- Subjects must have a, b, c, d, AND e

a) Clinical diagnosis of acute bacterial pneumonia acquired in the community. Subjects must have resided in the community (i.e., NOT in a chronic care facility such as a nursing home or rehabilitation facility) and NOT have been hospitalized during the 14 days before the onset of

symptoms of pneumonia. Residence in an assisted-living facility where the subject has regular access to the community is allowed.

b) Clinical signs or symptoms of acute bacterial pneumonia with AT LEAST 2 of the following criteria:

- Cough (new or increased over usual state)
- Production of purulent sputum or a worsening in the character of sputum
- Auscultatory findings on pulmonary examination of rales or evidence of pulmonary consolidation (dullness on percussion, bronchial breath sounds, or egophony)
- Dyspnea or tachypnea (respiratory rate ≥20 breaths per minute), that is new or worse than usual state
- New onset hypoxemia on room air (PO_2 <60 mmHg on arterial blood gas or O_2 saturation <90% by pulse oximetry), or respiratory failure requiring mechanical ventilation.

c) New radiographic infiltrates (not related to another disease process) consistent with the diagnosis of bacterial pneumonia

d) Fever or leukocytosis/leukopenia (temporally associated with the onset of pneumonia symptoms) with AT LEAST 1 of the following:

- Fever (in the absence of antipyretics) defined as an axillary temperature >37.5°C, oral temperature >38°C, a tympanic temperature >38.5°C, a rectal temperature >39°C, OR hypothermia, defined as a rectal body temperature of <35°C.
- Leukocytosis defined as an elevated total peripheral WBC count ≥10 x 10^9/L or ≥15% immature neutrophils (bands), regardless of total peripheral white count; OR leukopenia with total WBC ≤4.5 x 10^9/L.

e) Severity of pneumonia requiring i.v. antibiotic therapy.

4.3. Exclusion Criteria*

Potential subjects who meet any of the following criteria will be excluded from participating in the study:

* This section of the protocol has been revised. Please refer to the section of this document entitled PROTOCOL AMENDMENTS (Amendment INT-3, 22 December 2006) for a detailed description of the specific changes.

- History of or suspected condition that may jeopardize adherence to protocol requirements (e.g., severe cardiac disease such that even minimal physical activity causes discomfort, New York Heart Association [NYHA] Class 4)[47]
- History of or suspected hypersensitivity to any related anti-infective (including beta-lactam antibiotics such as penicillins and cephalosporins, oxazolidinones). Subjects in whom cephalosporins would be used as part of normal clinical practice are not excluded.
- History of or suspected condition or concurrent treatment that would be contraindicated by the prescribing information for ceftriaxone or linezolid
- History of or suspected severe renal impairment, (i.e., Cl_{Cr} ≤30 mL/min or oliguria <20 mL/h unresponsive to fluid challenge) or any form of dialysis
- History of or suspected hepatic dysfunction (total bilirubin, or alanine aminotransferase [ALT], or aspartate aminotransferase [AST] ≥3 times the upper limit of the normal range [ULN]).
- History of suspected extra-pulmonary infection, including concomitant meningitis, endocarditis, septic arthritis, or osteomyelitis
- Known to be human immunodeficiency virus (HIV) positive with CD4 counts of $\leq 0.2 \times 10^9$/L (≤200 cells/mm^3). (Subjects with HIV and $>0.2 \times 10^9$/L [>200 cells/mm^3] may be included)
- Presence of neutropenia, (absolute neutrophil count [ANC] $\leq 0.5 \times 10^9$/L [<500 Neutrophils [polymorphonuclear leukocytes] [PMNs/mm^3]) severe anemia (hemoglobin <6.5 g/dL), or severe thrombocytopenia ($<49.9 \times 10^9$/cm). *Note: Subjects receiving immunosuppressive therapy who are expected to reach a nadir of <500 PMNs/mm^3 during administration of study drug should not be enrolled*
- Previous enrollment in this study or any other ceftobiprole protocol
- Have received an experimental drug or used an experimental medical device within 30 days before the planned start of treatment
- Is pregnant or breast-feeding
- Employees of the investigator or study center, with direct involvement in the proposed study or other studies under the direction of that investigator or study center, as well as family members of the employees or the investigator

Clinical conditions that may interfere with assessments of efficacy:

- Sustained shock
- Subjects with any of the following pulmonary conditions:
  – known bronchial obstruction or a history of postobstructive pneumonia

- primary lung cancer or another malignancy to the lungs unless surgically resected
- cystic fibrosis
- lung abscess
- pleural effusion as a primary source of infection
- active tuberculosis
- suspected or known pneumonia due to aspiration, atypical bacteria (*Legionella* spp, *Mycoplasma pneumoniae*, and *Chlamydia pneumoniae*), viruses, or *Pneumocystis jiroveci (carinii)*.

Note – Subjects with asthma or chronic obstructive pulmonary disease are not excluded provided they meet the criteria of acute new-onset pneumonia.

Microbiological conditions that may interfere with assessments of efficacy:

- Systemic antimicrobial therapy for more than 24 hours in the 3 days before enrollment.

  Exception: Systemic antimicrobial therapy for more than 24 hours is permitted in the case of a subject with an infection caused by microbiologically confirmed pathogen(s) that are resistant to the previous antimicrobial agents (e.g., pneumonia due to a macrolide resistant staphylococci or pneumococci being treated with a macrolide).

4.4. Prohibitions and Restrictions

Potential subjects must be willing to adhere to the following prohibitions and restrictions during the course of the study to be eligible for participation.

- Women must avoid pregnancy through the use of contraception or abstinence during the course of this study. The same method of contraception must be continued throughout the study. Pregnancy testing will be done at screening and at the TOC visit.
- Use of nonstudy antibacterial agents is prohibited until after the LFU.

5. RANDOMIZATION AND BLINDING

5.1. Overview

Randomization will be used to avoid bias in the assignment of subjects to treatment, to increase the likelihood that known and unknown subject attributes (e.g., demographics and baseline characteristics) are evenly balanced across treatment groups, and to enhance the validity of statistical comparisons across treatment groups. Blinded treatment will be used to

reduce potential bias during data collection and evaluation of clinical endpoints.

5.2. Procedures

Subjects will be assigned to 1 of 2 treatment groups based on a computer-generated randomization schedule prepared by the sponsor before the study. The randomization will be balanced between the 2 treatment groups by using randomly permuted blocks. Subjects will be stratified at entry by PSI score to 1 of 2 strata: ≤90 and ≥91. Subjects will also be stratified by the need for anti-staphylococcal therapy (placebo or linezolid) based on signs, symptoms, and medical history at enrollment. Based on this randomization code, the study drug will be packaged and labeled for each subject. Medication code numbers will be preprinted on the study drug labels and assigned as subjects qualify for the study and are randomly assigned to treatment. An unblinded monitor will perform drug accountability.

Central randomization will be implemented in conducting this study. The subject number and treatment regimen will be assigned after phoning into the Interactive Voice Response System (IVRS) provided by a vendor designated by the sponsor. The caller must use their own user ID and PIN, and then give the requested subject details (e.g., subject initials and subject's date of birth). Based on this information, the IVRS will assign a unique subject number and treatment regimen, which will dictate the treatment assignment for that subject.

The investigator will not be provided with randomization codes. The codes will be maintained within the IVRS, which has the functionality to allow the investigator to break the blind for an individual subject.

Under normal circumstances, the blind should not be broken until all subjects have completed the study and the database is finalized. Otherwise, the blind should be broken only if specific emergency treatment would be dictated by knowing the treatment status of the subject. In such cases, the investigator must contact the sponsor. If the investigator is unable to contact the sponsor, the investigator may in an emergency determine the identity of the treatment by telephoning IVRS. The sponsor must be informed as soon as possible. The date, time, and reason for the unblinding must be documented in the appropriate section of the case report form (CRF) and in

the source document. The fax copy received from the IVRS indicating the code break must be retained with the CRF.

6. DOSAGE AND ADMINISTRATION

6.1.1. Overview*

The unblinded pharmacist will be responsible for preparation of the study drug for each subject in such a way that investigators and staff remain blinded to the study drug being administered. Colored sleeves will cover infusion bags and line tubings as needed to maintain the blind. The study drug regimen is discussed in Section 6.1.2, Intravenous and Oral Switch Study Drug Regimen and Section 6.1.3, Linezolid/Placebo Therapy for ceftriaxone-resistant *S. pneumoniae* and *S. aureus*.

All subjects should receive the first dose of study drug as quickly as possible after randomization. In order to ensure subject safety and to avoid potential overdose, if a subject has received a nonstudy antibiotic before enrollment, the timing of the first dose of study drug may be delayed so it is consistent with the recommended dosing interval of the nonstudy antibiotic. In the event that a subject received a nonstudy, beta-lactam antibiotic (e.g., penicillin, cephalosporin, carbapenem) before enrolling in the study, the timing of the first dose of study cephalosporin (ceftobiprole or ceftriaxone) may be delayed. All subjects should receive a minimum of 3 days (72 hours [9 doses of ceftobiprole or 3 doses of ceftriaxone; with placebo administered as described below to maintain the blind]) of the i.v. study drug and should continue on i.v. therapy while hospitalized. At the discretion of the investigator, subjects may receive study drug infusions as outpatients or through a home-care agency, provided they meet all other protocol requirements, but all i.v. study drug infusions must be administered and monitored by a qualified nurse, a qualified member of the study staff, or an infusion specialist. If after 3 days (72 hours) the subject's signs and symptoms have improved from baseline as defined in the protocol and the subject will be discharged from the hospital, the i.v. therapy may be discontinued at the investigator's discretion and the subject treated with oral cefuroxime axetil 500 mg every 12 hours.

* This section of the protocol has been revised. Please refer to the section of this document entitled PROTOCOL AMENDMENTS (Amendment INT-3, 22 December 2006) for a detailed description of the specific changes.

Subjects should continue i.v. study drugs for as long as they are hospitalized. However, as discussed above, it is recognized that some subjects will improve to the point where it is appropriate, based on local medical practice,[44,45,48] to be discharged from the hospital and to complete antimicrobial therapy with oral medication. To align with this practice, it will be possible for subjects to complete antimicrobial therapy by switching to oral medications (cefuroxime axetil as discussed above) provided ALL of the criteria in Section 9.1.4, Criteria for Intravenous to Oral Switch, are met. Additionally, if it is the local custom for subjects to remain hospitalized for extended periods beyond the need for i.v. therapy for social or logistical reasons, the subject may, in this case, be switched to oral therapy if deemed necessary, while still hospitalized.

6.1.2. Intravenous and Oral Switch Study Drug Regimen

Study drug (i.v. plus oral) will be administered for 5 to 10 days. The total duration of study drug therapy (i.v. plus oral) for all subjects will be a minimum of 5 days and a target of 7 days. If, in the investigator's opinion, a subject requires additional days of study therapy, the duration of the therapy may be extended (up to a maximum of 10 days). Therapy can be extended to a maximum of 14 days after approval by the sponsor's Medical Monitor, for subjects with a history of persistent bacteremia or X-ray evidence of necrotizing pneumonia.

At the investigator's discretion, therapy may be stopped when the infection is considered clinically cured (based on normalization of the clinical signs and symptoms of infection and improvement in the chest X-ray, and after a minimum of 5 total treatment days).

Ceftobiprole and ceftriaxone will be administered as follows:

– Ceftobiprole will be administered at a dose of 500 mg in 250 mL of 5% dextrose in water (D_5W) every 8 hours at a constant-rate i.v. infusion over a period of 120 minutes (must not be administered through an i.v. line containing sodium chloride [e.g., saline, Ringer's Lactate solution]). Further details of the preparation and administration of ceftobiprole are presented in Attachment 2, Preparation and Administration of Intravenous Infusion Solutions of Ceftobiprole Medocaril (500 mg dose).
– Ceftriaxone will be administered at a dose of 2 g in 50 mL of D_5W every 24 hours by i.v. infusion over a period of 30 minutes.

Placebo will be used to maintain the blind for both cephalosporins:

- For subjects randomized to ceftobiprole, a 30-minute i.v. infusion of placebo of 50 mL of D_5W will be given every 24 hours concomitantly with ceftobiprole and will begin with the first dose of ceftobiprole.
- For subjects randomized to ceftriaxone, a 120-minute i.v. infusion of placebo of 250 mL of D_5W will be given every 8 hours concomitantly with ceftriaxone, and will begin with the first dose of ceftriaxone.

Note: The 30-minute infusion should be administered first (placebo or ceftriaxone), followed by the 120-minute infusion (ceftobiprole or placebo), to ensure that active treatment is delivered to the subject as soon as possible.

Optional oral switch regimen:

- Cefuroxime axetil 500 mg every 12 hours orally.

6.1.3. Linezolid/Placebo Therapy for Ceftriaxone-resistant *S. Pneumoniae* and *S. Aureus**

Antibiotic coverage of Ceftriaxone-resistant *S. pneumoniae (CRSP)*

Linezolid or placebo should be added to study treatment for subjects with confirmed CRSP provided the sensitivity of the isolate to linezolid has been confirmed.

Anti-staphylococcal Therapy

If the incidence of MRSA in CAP isolates is prevalent (i.e., greater than 15%) in the local institution or region, or additional subject-specific risk factors are present, such as a history of prior MRSA infection or a surveillance culture positive for MRSA, linezolid/placebo should be added to cover the possibility of *S. aureus* based on the presence of:

- Gram-stain from adequate sputum with gram-positive cocci in clusters as predominant organism (>10/hpf) or respiratory culture positive for *Staphylococcus* within 72 hours before randomization.

  In addition, 2 or more of the following criteria:
- Temperature >39°C

* This section of the protocol has been revised. Please refer to the section of this document entitled PROTOCOL AMENDMENTS (Amendment INT-3, 22 December 2006) for a detailed description of the specific changes.

- Heart rate >140 beats per minute
- Respiratory rate >30 breaths per minute
- Hemoptysis
- Leukopenia
- Influenza symptoms within last 2 weeks (cough, coryza, fever)
- Necrotizing lesion(s) on chest X-ray or CT scan
- Known local case(s) of PVLpositive *S. aureus* infections

Linezolid/placebo will be discontinued at 48 hours if *S. aureus* is not isolated from the baseline cultures.

If the subject meets the criteria listed above but a Gram stain cannot be immediately obtained (i.e., the sputum sample is obtained at night), linezolid can be added empirically until the Gram stain results are available. If the results do not show gram-positive cocci in clusters from an adequate specimen, then treatment with linezolid should be discontinued.

If the incidence of MRSA in CAP isolates is not prevalent (i.e., less than 15%) in the local institution or region, the investigator may wait for definitive confirmation of MRSA sensitive to linezolid in the respiratory specimen collected within 72 hours before randomization prior to adding linezolid/placebo.

The following dosage of linezolid/placebo will be administered:

– Linezolid will be administered to those subjects randomized to ceftriaxone, at a dose of 600 mg in 300 mL of D_5W every 12 hours by i.v. infusion over a period of 60 minutes. Placebo of 300 mL of normal saline (NaCl 0.9% solution) will be administered to those subjects randomized to ceftobiprole, at a volume of 300 mL every 12 hours by i.v. infusion over a period of 60 minutes. Normal saline (NaCl 0.9% solution) will replace D_5W as the placebo solution for ceftobiprole in order to minimize the amount of free water delivered to subjects who are randomized to receive additional coverage with linezolid/placebo as follows:

Ceftobiprole Arm	Ceftriaxone Arm
Placebo (50 mL D_5W) every 24 hours	Ceftriaxone (2g in 50 mL D_5W) every 24 hours
Ceftobiprole (500 mg in 250 mL D_5W) every 8 hours	Placebo (250 mL *normal saline*) every 8 hours)
Placebo (300 mL *normal saline*) every 12 hours	Linezolid (600 mg in 300 mL D_5W) every 12 hours

6.1.4. Dose Adjustment for Renal Impairment

Dose adjustment for renal impairment will be made according to Cl_{Cr} at enrollment and will be adjusted if there are any changes during the study. Additional assessments of serum creatinine or renal function may be performed at the local laboratory at any time during the study, as clinically indicated. NOTE: Although subjects with Cl_{Cr} of ≤30 mL/min are initially excluded, if a subject's Cl_{Cr} decreases to ≤30 mL/min after enrollment they may remain in the study and the dosage of study drug(s) should be changed accordingly. Placebo infusions should be added as needed to maintain the blind.

- Ceftobiprole: in subjects with moderate renal impairment (Cl_{Cr} >30 to ≤50 mL/min) ceftobiprole will be infused at a dose of 500 mg every 12 hours over 120 minutes. In subjects with severe renal impairment (Cl_{Cr} >10 to ≤30 mL/min) 250 mg of ceftobiprole will be infused every 12 hours over 120 minutes. Subjects whose Cl_{Cr} level falls to ≤10 mL/min are to be discontinued from the study.
- Ceftriaxone: No adjustment of ceftriaxone is recommended in subjects with renal impairment provided the daily dose is 2 g/day or less. To maintain the blind, subjects with Cl_{Cr} ≤50 mL/min, will receive placebo 2-hour infusions every 12 hours instead of every 8 hours.
- Linezolid: No adjustment is required.
- Cefuroxime axetil: Do not use in subjects with Cl_{Cr} <30 mL/min.

7. COMPLIANCE*

Qualified staff will administer i.v. study drug and details of each administration will be recorded in the CRF (including date, start time of infusion, dose, and whether the entire dose was administered).

Oral study drug will be dispensed by the pharmacist and the number of doses noted on the CRF. The subject will be instructed to return the study drug container and any remaining study drug at the EOT visit.

* This section of the protocol has been revised. Please refer to the section of this document entitled PROTOCOL AMENDMENTS (Amendment INT-3, 22 December 2006) for a detailed description of the specific changes.

8. CONCOMITANT THERAPY

Concomitant medications are recorded at baseline and throughout the study, in the appropriate section of the CRF. Treatment of pre-existing chronic diseases may be continued during the study.

No other systemic concomitant antibacterial treatment is permitted. The use of antiseptic in a bronchial lavage during the study is not allowed.

Any concurrent treatment that would be contraindicated by the prescribing information for linezolid, ceftriaxone, or cefuroxime axetil is prohibited.

The sponsor must be notified in advance (or as soon as possible thereafter) of any instances in which prohibited therapies are administered.

9. STUDY EVALUATIONS

9.1. Study Procedures

9.1.1. Overview

The Time and Events Schedule included in the Synopsis summarizes the frequency and timing of efficacy, safety, or other measurements.

The total volume of blood collected for laboratory evaluations throughout this study is approximately 121 mL for each subject (50 mL for safety, 10 mL for mycoplasma and chlamydia titers, 30 mL for blood cultures, 10 mL for repeat blood samples, and up to 21 mL for PK sampling [only 9 mL of blood will be drawn for those subjects participating in the sparse PK sampling, thereby yielding a total blood volume collected of 109 mL]). There will be 3 phases to this study: a prerandomization phase (baseline); a double-blind treatment phase with an EOT visit (within 48 hours after the end of therapy); and a follow-up phase with a TOC/early termination visit 7 to 14 days after the last dose of study drug, and a LFU visit 28 to 35 days after the last dose of study drug.

9.1.2. Prerandomization Phase

Subjects who have CAP of sufficient severity to necessitate hospitalization and treatment with i.v. antibiotics for at least 3 days (72 hours), will be asked to sign the informed consent form. The investigator will then complete the baseline assessments listed in the Time and Events Schedule and determine if the subject is eligible for enrollment in this study.

9.1.3. Double-Blind Treatment Phase

The total duration of study drug therapy (i.v. plus oral) for all subjects will be a minimum of 5 days and a target of 7 days. If, in the investigator's opinion, a subject requires additional days of study therapy, the duration of the therapy may be extended (up to a maximum of 10 days) (see Section 6, Dosage and Administration).

If by Day 3, a subject has resolution of signs and symptoms of pneumonia (including fever, WBC, tachypnea, chest X-ray), therapy can be discontinued after all doses on Day 5.

If a subject has a positive blood culture at baseline, they must remain on i.v. study drug for a minimum of 5 days pending results of repeat blood cultures.

Administration of study drug(s) beyond 7 days is recommended only for subjects with persistent signs and symptoms consistent with active infection. These criteria include fever ≥38.3°C, circulating WBC count greater than 10,000 mm^3, lack of improvement on chest X-ray, continued purulent sputum, or bacteremia at enrollment and at least one positive blood culture on therapy.

If more than 10 days are necessary to complete treatment, therapy may be further extended to a maximum of 14 days for subjects with a history of persistent bacteremia or necrotizing pneumonia.

Dosing will begin on Day 1 after baseline assessments are completed. Subjects will have daily assessments while in the hospital to ensure adequate clinical progress and to ascertain appropriateness for change to oral medication and discharge if desired.

Subjects with serum sodium levels outside of the normal range at baseline or during the study should have their electrolytes measured on a daily basis or more often as clinically indicated until the serum sodium level is within the normal range.

9.1.4. Criteria for Intravenous to Oral Switch*

Beginning on Day 4 all subjects will be evaluated daily to determine if they meet the criteria for optional switch to oral medication. Once the subject meets the IOS criteria (see below), daily assessments are no longer required. At any time after 3 days (72 hours) of i.v. therapy (between Days 4 and 14), unless the subject was bacteremic upon enrollment, if the subject meets the IOS criteria (see below) for improvement and hospital discharge is desirable, to a location where i.v. therapy is not possible, at the investigator's discretion, the subject may switch from using i.v. to using oral medication. (Change to oral medication is not required.) Assessments will be performed at the time of IOS, before hospital discharge. Subjects with pathogens resistant to cefuroxime axetil will need to complete the full course of i.v. therapy. In order to be eligible for switch to oral medication, a subject must meet ALL of the following criteria:

- Able to swallow and absorb oral medications
- Body temperature improved from baseline (rectal temperature ≥35°C and ≤38.8°C, axillary temperature ≥35.5°C and ≤37.3°C, oral temperature ≥36°C and ≤37.8°C, or tympanic temperature ≥36.5°C and ≤38.3°C) for at least 24 consecutive hours in the absence of any antipyretic medications
- White blood cell (WBC) count and bands (%) within the normal range for the laboratory
- Respiratory rate ≤20 breaths per minute

* This section of the protocol has been revised. Please refer to the section of this document entitled PROTOCOL AMENDMENTS (Amendment INT-3, 22 December 2006) for a detailed description of the specific changes.

- Systolic blood pressure ≥90 mmHg and pulse ≤100 beats per minute for at least 24 consecutive hours
- Oxygen saturation of ≥92% by pulse oximeter or $PO_2 \geq 80$ mmHg on arterial blood gas while subject is breathing room air OR return to documented pre-infection baseline
- Significant improvement in clinical symptoms and signs (cough, sputum production, ausculatory findings, chills, and chest pain) compared with enrollment visit (baseline)
- No clinically significant deterioration observed on the chest X-ray
- Negative blood cultures
  a. At the enrollment visit (baseline) OR
  b. If blood cultures obtained at the enrollment visit (baseline) are positive for a pathogen, blood cultures should be repeated and must be negative after a minimum of 3 days (72 hours) incubation.

These criteria for oral switch are consistent with standard medical practice and have been shown to be associated with a positive outcome[41,43] including in subjects with bacteremic *S. pneumoniae* pneumonia.[42]

At the investigator's discretion, therapy may be stopped anytime after the fifth day of therapy if the infection is considered clinically cured (based on normalization of the clinical signs and symptoms of infection and improvement in the chest X-ray).

If treatment has been extended to 10 days or to 14 days, and it is determined that the subject needs additional antibiotic therapy, the assessments (Visit 5 or 6) will be performed and any significant findings discussed with the Medical Monitor. If the subject does not need additional antibiotic therapy, the EOT assessments should be performed.

End-of-Treatment Visit

End-of-treatment assessments must be performed within 48 hours after the end of therapy. If a subject withdraws prematurely, the TOC/early termination visit assessments must be done at the time of withdrawal.

9.1.5. Follow-Up Phase

Test-of-Cure Visit

Study assessments will be performed as specified in the Time and Events Schedule at the TOC/early termination visit, which will occur 7 to 14 days after the last dose of study drug. In the event a subject discontinues study drug prematurely, the TOC/early termination visit assessments must be done at the time of study drug discontinuation.

Late Follow-Up Visit

Late follow-up (28 to 35 days after last dose of study drug) will be conducted by telephone unless an examination is needed to evaluate relapse (need for further antibiotic treatment) or abnormalities that were present at TOC visit.

9.2. Pharmacokinetic Evaluations*

All subjects will participate in either sparse or rich PK sampling.

9.2.1. Sample Collection and Handling*

Refer to the PK laboratory manual, which will be provided as a separate document (refer to Section 14, Study-Specific Materials) for information regarding handling of biological samples. The exact dates and times of blood sampling must be recorded either in the CRF or the laboratory requisition form as appropriate.

At selected sites for rich PK sampling:

Seven blood samples and 1 urine sample (over 8 hours) for each subject will be obtained for PK analysis at selected sites with appropriate facilities and equipment. Approximately 120 subjects (approximately 60 subjects on ceftobiprole treatment and approximately 60 subjects on ceftriaxone with or without linezolid) will participate in PK sample collections. The selected

* This section of the protocol has been revised. Please refer to the section of this document entitled PROTOCOL AMENDMENTS (Amendment INT-3, 22 December 2006) for a detailed description of the specific changes.

* This section of the protocol has been revised. Please refer to the section of this document entitled PROTOCOL AMENDMENTS (Amendment INT-3, 22 December 2006) for a detailed description of the specific changes.

sites may also include some elderly subjects (>65 years) for the PK sampling if they are available.

On Day 4±1, one 3 mL blood sample will be obtained immediately before the start of the 120-minute morning infusion (predose); subsequent 3-mL blood samples will be obtained at 15 minutes, 1, 2 (immediately before the end of infusion), 4, 6, and 8 hours after the start of the morning infusion. On this day, subjects will be asked to void and discard urine immediately before the start of the infusion. Subsequently, urine will be collected during the period of 0 to 8 hours after the start of the 120-minute morning infusion. Subjects will be encouraged to drink water ad libitum.

Subjects may refuse to participate in rich PK blood sampling and still be enrolled in the study. In this circumstance, sparse PK samples should be collected for these subjects.

At sites for sparse PK sampling:

Three sparse PK blood samples will be collected for each subject who does not participate in the rich PK sampling. On study Day 1, 2 PK blood samples (3 mL each) will be taken; one at approximately 2 hours (or immediately before the end of the first 120-minute infusion) and the other 6 hours after the start of this infusion (the first dose of the entire treatment). On Day 4±1, another PK blood sample (3 mL) will be taken at any time during the dosing interval, but starting at least 30 minutes after the onset of this 120-minute morning infusion. No PK urine sample will be collected for subjects with sparse sampling.

9.2.2. Analytical Procedures

Plasma samples will be analyzed for ceftobiprole by a validated specific liquid chromatography coupled to tandem mass spectrometer (LC-MS/MS) method. Urinary concentrations of ceftobiprole will be analyzed by LC-MS/MS. The sponsor or its designee will perform all analyses.

9.2.3. Pharmacokinetic Parameters*

In order to develop a structural PK model of ceftobiprole and to evaluate the dependence of PK of the active ceftobiprole on population covariates, all PK data from subjects in this study with both rich and sparse sampling will be combined with data from other clinical studies of ceftobiprole for a pooled population PK analysis. This pooled analysis will be reported separately.

Pharmacokinetic parameters will be derived by noncompartmental analysis using WinNonlin™ (Version 4.0.1 or an upgraded version if available, Pharsight Corporation, California). Pharmacokinetic parameters will include AUC_{τ}, C_{max}, CL, and Ae of ceftobiprole. In addition, time above MIC of ceftobiprole will be determined (assuming MIC of 4 μg/mL).

- C_{max} maximum drug concentration observed in plasma
- AUC_{τ} area under the plasma concentration versus time curve from time during the dosing interval (τ = 8 hours)
- CL Total systemic clearance, estimated by dose/AUC_{τ}
- Ae Amount excreated into the urine, calculated by the product of urinary volume times the urinary concentration over the collection interval

Plasma concentrations at each time point of measurement will be evaluated by descriptive statistics, including arithmetic mean, standard deviation, minimum, maximum, and median.

All PK parameters will be presented in listings and using descriptive summary statistics.

For subjects with sparse PK sampling, plasma concentrations will be listed with the corresponding sampling time for all subjects.

9.3. Efficacy Evaluations

9.3.1. Clinical Efficacy Assessments

Clinical assessments will be done during the study after review of the inclusion/exclusion criteria and documented informed consent has been obtained.

* This section of the protocol has been revised. Please refer to the section of this document entitled PROTOCOL AMENDMENTS (Amendment INT-3, 22 December 2006) for a detailed description of the specific changes.

At the baseline (Day 1) assessment, the following clinical signs and symptoms will be reported as present or absent:

- Cough
- Production of purulent sputum or respiratory secretions
- Presence of rales or evidence of pulmonary consolidation on chest auscultation
- Rigors or shaking chills
- Pleuritic chest pain
- Tachypnea
- Hypoxemia

At all subsequent assessments, changes in signs and symptoms will be reported in comparison with the baseline (Day 1) assessment as:

- Worsened from baseline visit
- Unchanged from baseline visit
- Improved from baseline visit
- Absent

Overall clinical assessment will be compared with the baseline (Day 1) visit:

- Worsened from baseline visit
- Unchanged from baseline visit
- Improved from baseline visit
- Cured

At the TOC assessment, the clinical outcome will be rated as follows:

Cure: resolution of signs and symptoms of the infection (return to pre–infection baseline), or improvement to such an extent that no further antimicrobial therapy is necessary and improvement or stabilization of chest X-ray findings.

Failure: death due to pneumonia occurring more than 48 hours after first infusion or a lack of efficacy (incomplete resolution of signs and symptoms of the infection, i.e., remains worse than pre-infection baseline or worsening of the chest X-ray) and requirement for additional nonstudy antibiotics to treat this episode of pneumonia after at least 48 hours of study therapy.

Unable to evaluate: Discontinued early (before completing full course of study antibiotic) for any reason other than lack of efficacy and requires further antibiotics.

Subjects, who have missing data from the TOC visit, will be considered clinical failures, if they are worsened or unchanged from baseline at the EOT visit, provided that they meet no other criteria for nonevaluability.

At the LFU assessment, the TOC clinical outcome of failure or unable to evaluate will be carried forward as the LFU outcome assessment. Subjects with TOC clinical outcome of cure will be categorized at the LFU assessment as:

Cure: no further antimicrobial therapy was necessary for treatment of the infection.

Relapse: signs and symptoms of CAP reappeared such that additional antimicrobial therapy was necessary. Relapse will be categorized as failure at LFU.

Unable to evaluate: Absence of clinical assessments at LFU, or concomitant treatment with a systemic antibiotic active against gram–positive or gram–negative respiratory bacteria, administered for a reason other than the CAP.

9.3.2. Microbiological Procedures and Efficacy Assessments

Samples for appropriate culture (respiratory secretions and blood) must be obtained before the start of study therapy. Microbiological procedures will be done according to local practice. The sponsor will provide E-test® and Discs for susceptibility testing to ceftobiprole. Quantification or respiratory cultures should be recorded according to standard procedures of the local laboratory (see Attachment 3, Procedure for Collection, Culture, Susceptibility Testing, Storage, and Shipment of Microbiological Samples).

An aliquot of each potential pathogen isolated, including duplicate (follow–up) isolates from all assessments, will be shipped to the central laboratories for centralized antimicrobial susceptibility testing and pathogen identification, using Clinical and Laboratory Standards Institute (CLSI) methods. Sample handling and shipping will follow instructions provided by

the central laboratories. In cases where pathogens are characterized at the study center and centrally, the central laboratory data will be considered definitive.

The clinical investigator should be informed at the earliest occasion (overnight culture, 48–hour report) of suspected infection/colonization/ superinfection with resistant pathogens.

The sponsor will provide the test kit for *L. pneumophila* urine antigen testing, which will be performed by the local laboratory.

9.3.3. Microbiological Samples and Testing*

Respiratory Culture, Gram Stain and Sensitivity Testing

Respiratory specimens should be obtained within 24 hours before the first dose of study drug. In the event that a subject has received nonstudy antibiotics before study enrollment, respiratory cultures obtained before, or as soon as possible, but within 24 hours after the start of nonstudy antibiotic treatment must be available. This culture must be repeated, if necessary, so that a respiratory specimen is collected within 24 hours before the first dose of study drug. All procedures should be performed according to local best practice. Specimens obtained by deep expectoration or nasotracheal aspiration are only acceptable in nonventilated subjects. Suitable specimens from expectorated samples are defined as having <10 squamous epithelial cells and >25 leukocytes per low powered field (LPF) (10x objective). In a Gram stain, an organism is considered predominant if there are >10 organisms per oil-immersion field in 10 fields (100x objective). Suitable specimens for ventilated subjects include bronchoscopy with BAL/protected-brush sampling, or transtracheal/endotracheal aspiration at baseline for Gram stain, culture, and sensitivity testing. Where possible, invasive specimens (BAL or protected–brush sampling) with quantitative cultures should be obtained on all ventilated subjects.

In all cases of clinical failure, new cultures must be attempted, with susceptibility testing on isolated pathogens.

* This section of the protocol has been revised. Please refer to the section of this document entitled PROTOCOL AMENDMENTS (Amendment INT-3, 22 December 2006) for a detailed description of the specific changes.

If a primary isolate was unsuccessfully transferred to the central laboratory, the local laboratory results may be considered as definitive.

See Attachment 3, Procedure for Collection, Culture, Susceptibility Testing, Storage, and Shipment of Microbiological Samples.

Blood Cultures

At least 2 sets of aerobic/anaerobic blood cultures must be obtained according to local practice for each subject, within 24 hours before the start of treatment. In the event that a subject has received nonstudy antibiotics before study enrollment, blood cultures obtained before, or as soon as possible, but within 24 hours after the start of nonstudy antibiotic treatment must be available. These cultures must be repeated, if necessary, so that a blood culture is collected within 24 hours before the first dose of study drug. At least 1 culture should be taken from a peripheral vein. Cultures must either be drawn one after the other from different sites or drawn from the same site, 10 minutes apart. Any potential pathogen should be stored and shipped to the central laboratory.

Positive blood cultures at baseline or follow-up (concordant growth of the same pathogen in at least 2 bottles or corresponding to a respiratory isolate) must be repeated at each visit or more frequently, if indicated, until negative cultures are obtained. If blood cultures on 3 consecutive days are positive, the subject should be withdrawn from the study and considered a treatment failure. If negative blood cultures are obtained at baseline, blood cultures should be repeated at subsequent visits only if clinically indicated. Culture procedures will be according to local practice.

Urine Antigen Testing

At the screening visit, a urine sample must be obtained and *L. pneumophila* antigen testing performed by the local laboratory.

The Binax NOW® Legionella Urinary Antigen Test is an immunochromatographic membrane assay (ICT) to detect *L. pneumophila* serogroup 1 soluble antigen in human urine. It is not technically demanding and requires no specialized laboratory equipment. Using concentrated urine, it has a sensitivity of 70% to 95% for patients with moderate to severe community-acquired Legionnaires' disease.[33,37,49] Its purpose in this study is

to quickly rule out *L. pneumophila* as a cause of the subject's pneumonia so they can be discontinued and treated appropriately (the study drug regimens are not effective against *Legionella* spp). Urinary Legionella antigen testing will be performed in all subjects.

A serum blood sample for mycoplasma and *C. pneumoniae* titers will be evaluated at baseline. Follow-up titers will be obtained at the LFU visit for treatment failures.

9.3.4. Assessment of Microbiological Outcome

In all cases of clinical failure (see Section 9.3.1, Clinical Efficacy Assessments), samples for new cultures must be obtained and all pathogens isolated will be shipped to the central laboratory for re-identification and susceptibility testing.

Based on information provided on the CRF, at the TOC assessment the sponsor will categorize each pathogen isolated from admission culture (respiratory or blood) according to the following information.

Eradication: No growth of a potential pathogen from a culture taken at the original site of infection.

Presumed Eradication: No culturable material can be obtained from the original site of infection, in the absence of clinical signs or symptoms of infection.

Colonization: Growth of a potential pathogen other than the original pathogen from a culture taken at any time after baseline at the original site of infection, in the absence of clinical signs or symptoms of infection.

Persistence: Growth of the original pathogen from a culture at the TOC (or early termination) taken at the original site of infection, with/without the presence of clinical signs or symptoms of infection.

Presumed Persistence: No culturable material can be obtained from the original site of infection, in the presence of clinical signs or symptoms of infection.

Superinfection: Growth of a pathogen other than the original pathogen from a culture taken at any time after baseline at the original site of infection, in the presence of clinical signs or symptoms of infection.

Unable to evaluate: Not able to evaluate microbiological outcome because:

- No pathogen identified at baseline, or
- Absence of clinical determination at TOC, or
- Requires further antibiotics for conditions other than CAP, or
- Other (specified by the investigator).

Only microorganisms from valid samples and those generally accepted as pathogens will be considered when determining the microbiological evaluability of a subject. Isolates from invalid samples may be used for resistance development investigations.

9.3.5. Chest X-ray*

Chest X-rays (preferably both posteroanterior and lateral views) will be obtained according to the Time and Events Schedule, and at any other time deemed appropriate by the investigator. Chest X-rays should be interpreted by the investigator, a pulmonologist, or radiologist and interpreted with respect to the baseline chest X-ray for clinical management of the subject during the study. Chest X-rays obtained from the predose and TOC visits will be interpreted at a central location. The chest x-rays will be centrally read to confirm the presence of a qualifying infiltrate at baseline and to confirm the site interpretation at TOC.

9.4. Efficacy Criteria

Primary Endpoint

The clinical cure rate is defined as the ratio of the number of clinically cured subjects to the total number of subjects in the population at the TOC visit.

Secondary Endpoints

- Microbiological eradication rate at TOC visit
- Clinical cure rate in subjects requiring mechanical ventilation within 48 hours of enrollment
- Microbiological cure rate in subjects requiring mechanical ventilation within 48 hours of enrollment

* This section of the protocol has been revised. Please refer to the section of this document entitled PROTOCOL AMENDMENTS (Amendment INT-3, 22 December 2006) for a detailed description of the specific changes.

- Clinical and microbiological relapse at LFU visit
- Thirty-day pneumonia-specific mortality rates (all deaths due to pneumonia within 30 days after randomization).

For the definition of these parameters see Section 11.4.2, Definition of Parameters.

9.5. Safety Evaluations*

The study will include the following evaluations of safety and tolerability (refer to Time and Events Schedule for when these assessments are to be completed):

- **Adverse Events**
  Adverse events will be reported by the subject (or, when appropriate, by a caregiver, surrogate, or the subject's legally acceptable representative) for the duration of the study. Adverse events will be followed by the investigator for a length of time as determined by the sponsor. Specific details on adverse event reporting are provided in Section 12.
- **Clinical Laboratory Tests**
  Blood samples will be collected for serum chemistry and hematology and a random urine sample will be collected for urinalysis. The investigator must review the laboratory report, document this review, and record any clinically relevant changes occurring during the study in the adverse event section of the CRF. The following tests will be performed by the central laboratory:

  Hematology Panel

hemoglobin	ANC
hematocrit	platelet count
red blood cell (RBC) count	
WBC count with differential	

Subjects with moderate anemia (hemoglobin between 6.5 and 7.9 g/dL) or moderate thrombocytopenia (50.0 to 74.9 x 10^9/cm) should have a complete blood count performed at least every other day.

* This section of the protocol has been revised. Please refer to the section of this document entitled PROTOCOL AMENDMENTS (Amendment INT-3, 22 December 2006) for a detailed description of the specific changes.

Serum Chemistry Panel

sodium
potassium
urea
bicarbonate
AST
ALT
glucose
gamma glutamyltransferase (GGT)
total bilirubin
serum creatinine
alkaline phosphatase
creatine phosphokinase
lactate dehydrogenase (LDH)
uric acid
calcium
triglycerides
albumin
total protein
cholesterol
chloride

Serum for Mycoplasma pneumoniae and Chlamydia pneumoniae antibody titers

Creatinine clearance will be estimated by use of the Cockcroft and Gault equation.

Men: Cl_{Cr} (mL/min) = Weight (kg) x (140 – age in years)/ 72 x serum creatinine (mg/dL)

Women: 0.85 x above value

To convert serum creatinine in mol/L to mg/dL divide by 88.4.

Additional tests of liver function will be performed if indicated.

Subjects with serum sodium levels outside of the normal range at baseline or during the study should have their electrolytes measured on a daily basis or more often as clinically indicated until the serum sodium level is within the normal range.

Urinalysis

Dipstick
specific gravity
pH
glucose
protein
blood
ketones
bilirubin
urobilinogen
nitrite

Sediment*
RBC
WBC
epithelial cells
crystals
casts
bacteria

leukocyte esterase

*If positive dipstick results are obtained for blood or protein, a microscopic examination of sediment is required.

A urine or serum monoclonal antibody test for β-hCG must be performed at baseline and at the TOC visit for all women of childbearing potential. The result must be negative before first dosing of study drug. Additional serum or urine pregnancy tests will be conducted throughout the study in sufficient number, as determined by the investigator, to establish the absence of pregnancy during the study.

Note: Blood samples for WBC with differential and chemistries (electrolytes and renal and liver function tests) for evaluation of subject eligibility, as well as other samples deemed essential by the investigator for clinical management may be sent to the local laboratory, as necessary.

- **Electrocardiogram**
  Twelve-lead ECGs will be recorded at a paper speed of 25 mm/sec so that the different ECG intervals (RR, PR, QRS, QT) can be measured.
- **Vital Signs** (temperature, pulse, respiratory rate, and blood pressure) must be measured twice daily at least 8 hours apart, while the subject is hospitalized (in addition to other time points listed in the Time and Events Schedule). The vital signs that reflect the highest temperature recorded for the subject on that day should be captured in the CRF.
- **Physical Examination**

Any clinically significant treatment-emergent abnormalities persisting at the end of the study will be followed by the investigator until resolution or until reaching a clinically stable endpoint.

Independent Data Monitoring Committee

An IDMC will be established to monitor data on an ongoing basis to ensure the continuing safety of the subjects enrolled in this study and efficacy of the study drug. Thereafter, the committee will meet periodically to ensure continued safety and efficacy. After each review, the IDMC will make recommendations regarding the continuation of the study. The details will be provided in a separate IDMC charter.

The IDMC will consist of independent, multidisciplinary experts relevant to the study. The IDMC responsibilities, authorities, and procedures will be documented in its charter. Minutes of IDMC meetings will be provided to

the sponsor with comments and recommendations after the completion of the study in keeping with the established charter.

9.6. Medical Resource Utilization Data Collection

During the study, and until completion of LFU, MRU data associated with medical encounters related to CAP or treatment (e.g., intubation, placement of chest tubes, tracheostomy) will be collected in the CRF by the investigator staff for all subjects. Protocol mandated procedures, tests, and encounters are excluded. These data will be used to conduct exploratory economic analyses.

Specifically, data collected will include:

- Number and duration of medical care encounters
- Duration of hospitalization
- Duration of hospitalization in ICU setting
- Number and character of diagnostic and therapeutic tests and procedures
- Concomitant medications

10. SUBJECT COMPLETION/WITHDRAWAL

10.1. Completion

A subject will be considered as having completed the study if he/she has completed all assessments at the LFU visit and received at least 5 days of study treatment. Subjects who discontinue study treatment after 48 hours of treatment due to lack of efficacy are also considered to have completed the study.

10.2. Discontinuation of Treatment

A subject should be discontinued from study treatment if:

- the investigator believes that for safety reasons (e.g., adverse event) it is in the best interest of the subject to stop treatment
- the subject becomes pregnant
- creatinine clearance is <10 mL/min or the subject requires dialysis
- significant myelosuppression occurs during therapy with linezolid/placebo; treatment with linezolid/placebo should be stopped unless it is considered absolutely necessary to continue therapy, in which case, intensive monitoring of blood counts and appropriate management strategies should be implemented
- optic or peripheral neuropathy occurs during therapy with linezolid/placebo; treatment with linezolid/placebo should be stopped

unless it is considered absolutely necessary to continue therapy. Optic and peripheral neuropathy are potential adverse effects of linezolid therapy. Subjects will be educated on symptoms associated with optic neuropathy (e.g., acute blurred vision, vision loss, color loss) and peripheral neuropathy (e.g., paresthesia, sensory loss) and instructed to report them if they occur. If subjects report symptoms of visual impairment, such as changes in visual acuity, changes in color vision, blurred vision, or visual field defect, prompt ophthalmic evaluation is recommended. Visual function should be monitored in all subjects reporting new visual symptoms, regardless of the length of linezolid therapy. Subjects with significant or progressive symptoms will be discontinued from linezolid/placebo therapy and referred for specialty evaluation if symptoms persist.

If a subject discontinues treatment early, TOC assessments should be performed at the time of study drug discontinuation. The subject should be contacted at the LFU visit to obtain MRU information and to follow up on any ongoing adverse events.

10.3. Withdrawal From the Study

A subject will be withdrawn from the study for any of the following reasons:

- lost to follow-up
- withdrawal of consent (subjects who withdraw consent should be treated appropriately).

In case a subject is lost to follow-up, every possible effort must be made by the study site personnel to contact the subject and determine the reason for discontinuation. The measures taken to follow up must be documented.

When a subject withdraws before completing the study, the reason for withdrawal is to be documented on the CRF and in the source document. Study drug assigned to the withdrawn subject may not be assigned to another subject. Subjects who withdraw will not be replaced.

11. STATISTICAL METHODS*

11.1. Sample Size Determination

The sample size determination is based on a noninferiority test of ceftobiprole versus the comparator in terms of the primary efficacy parameter, clinical cure rate. The assumptions on which the sample size estimate is based are as follows:

- Clinical cure rates of 90% for both randomized study treatments ceftobiprole and the comparator
- Limit of noninferiority of ceftobiprole versus the comparator is 10%
- Type 1 error probability (α) = 0.05 (2 sided)
- Type 2 error probability (β) = 0.2 (power = 0.8 = 1 – β)
- The evaluable rate is 80%.

Based on these assumptions, 670 subjects will need to be randomized to ensure 532 clinically evaluable subjects, 266 subjects in each treatment group.

11.2. Pharmacokinetic Analyses

Within each subject group, individual and overlay plasma concentration-time profiles of ceftobiprole will be plotted on both linear and log scales. Mean and median plasma concentration-time profiles will be graphically presented for each subject group. Plasma and urine concentration data at each time point will be summarized per subject group with mean, median, standard deviation, minimum value, maximum value, and coefficient of variation (%).

All estimated plasma and urine PK parameters of ceftobiprole will be summarized per subject group with mean, median, geometric mean, minimum value, maximum value, standard deviation, and coefficient of variation (%).

* This section of the protocol has been revised. Please refer to the section of this document entitled PROTOCOL AMENDMENTS (Amendment INT-3, 22 December 2006) for a detailed description of the specific changes.

11.3. Analyses Methods*

11.3.1. Definition of Populations

The following subject populations will be defined for the analyses:

Intent-to-Treat (ITT) Population: The set of all randomized subjects.

Modified Intent-to-Treat (mITT) Population: The subset of randomized subjects with a confirmed pathogen in baseline samples.

Clinically Evaluable (CE) Population: The subset of the ITT population who received at least 1 dose of study drug, excluding those subjects with a clinical outcome of Unable to Evaluate at the TOC visit and who have <5 days of study treatment. Those who have at least 2 days of treatment but who are judged as clinical failures will be evaluable.

Microbiologically Evaluable (ME) Population: All randomized subjects who received at least 1 dose of study drug with a confirmed pathogen in baseline sample, excluding those subjects with a microbiological outcome of Unable to Evaluate at the TOC visit.

Safety Population: All randomized subjects who are exposed to study drug.

11.3.2. Definition of Parameters

Primary

- Clinical cure rate, defined as the ratio of the number of clinically cured subjects to the total number of subjects in the population, at TOC visit.

Secondary

- Microbiological eradication rate, defined as the ratio of the number of subjects with microbiological eradication or presumed eradication to the total number of subjects in the population at TOC visit.
- Clinical cure rate following treatment with ceftobiprole versus the comparator of hospitalized CAP subjects that require mechanical ventilation within the first 48 hours of enrollment

* This section of the protocol has been revised. Please refer to the section of this document entitled PROTOCOL AMENDMENTS (Amendment INT-3, 22 December 2006) for a detailed description of the specific changes.

- Microbiological eradication rate following treatment with ceftobiprole versus the comparator of hospitalized CAP subjects that require mechanical ventilation within the first 48 hours of enrollment.
- Clinical and microbiological relapse at LFU visit
- Thirty-day pneumonia-specific mortality rates following treatment with ceftobiprole or the comparator.

Other:

- Assessment of the PK of ceftobiprole in subjects treated with ceftobiprole
- Collection of MRU data that may be used in future economic modeling

Safety/Tolerability

- Safety and tolerability of treatment with ceftobiprole in subjects hospitalized with CAP.

11.3.3. Subject Baseline Characteristics and Disposition

Demographics and baseline characteristics will be summarized descriptively.

Subject disposition will be summarized, including the numbers of subjects who prematurely discontinued the trial treatment, who underwent the TOC visit and who returned for the LFU visit.

Subjects who discontinued treatment prematurely will be listed and summarized, including details of the reasons for discontinuation.

Concomitant treatments will be summarized descriptively.

11.3.4. Analysis of Primary Parameter

The primary analysis of clinical cure rate will be based on the CE population and the ITT population as coprimary populations.

A noninferiority hypothesis will be assessed based on clinical cure rates at the TOC visit. The hypotheses will be:

- H_0: The clinical cure rate of ceftobiprole is more than 10% inferior to that of the comparator.
- H_1: The clinical cure rate of ceftobiprole is not more than 10% inferior to that of comparator.

This hypothesis will be operationally tested by presenting the 2-sided 95% confidence limits of the between treatment difference in clinical cure rate at the TOC visit. In order to claim noninferiority, the lower bound of the 2−sided 95% confidence interval must be ≥-10%.

In addition, clinical outcome at the TOC visit will be summarized descriptively by initial pathogen, subject subgroups defined by age (<65 versus ≥65 years), by sex (men versus women), and by race.

The analysis of clinical cure rate will be performed in the mITT populations as well. Subjects in the ITT and mITT populations whose clinical outcome is assessed as missing or Not Evaluable, will be included in the analysis of clinical outcome as failures.

Exploratory analyses of clinical outcome (cure versus failure) based on logistic regression models adjusting for geographical region and baseline factors may be performed.

11.3.5. Analysis of Secondary Parameters

Microbiological eradication rate will be analyzed in a similar manner to clinical cure rate at the TOC visit. In addition, microbiological eradication rate at the TOC visit will be summarized descriptively by infection type and initial pathogen as well as by subjects with and without quantitative respiratory culture at baseline.

The status of subjects with clinical cure at the TOC visit with regard to relapse (failure at the LFU visit) will be summarized descriptively.

The correlation between cure and microbiological eradication rates will be presented descriptively in the form of frequency tables.

The 30-day pneumonia-specific mortality rate will be analyzed to compare the 2 treatment groups using a Fisher's exact test.

11.3.6. Analyses of Other Parameters

The duration of treatment and MRU data will be summarized descriptively.

Subgroup analysis (by geographical region or by country) of resistance status will be summarized descriptively.

11.4. Safety Analyses

All safety analyses will be performed on the safety population.

All safety parameters will be listed in individual subject listings, sorted by randomized trial treatment group, center, and subject number.

Adverse Events

The original terms used in the CRFs by investigators to identify adverse events will be coded using the Medical Dictionary for Regulatory Activities (MedDRA). The percentage of subjects with specific treatment-emergent adverse events will be summarized for each treatment group. Adverse events will be summarized in frequency tables by severity and relationship to study drug.

Special attention will be given to those subjects who have discontinued treatment due to an adverse event or who experienced a severe or a serious adverse event.

Clinical Laboratory Tests

Laboratory data will be summarized by the type of laboratory test. Normal reference ranges and markedly abnormal results (specified in the Statistical Analysis Plan) will be used in the summary of laboratory data. Descriptive statistics will be calculated for each laboratory analyte at baseline and at each scheduled time point. Changes from baseline results will be presented in pre- versus posttreatment cross tabulations (with classes for below, within, and above normal ranges). A listing of subjects with any laboratory results outside the reference ranges will also be provided.

Cardiovascular Safety

ECG data will be summarized using descriptive statistics.

Vital Signs and Physical Examination

Descriptive statistics will be provided to evaluate the changes at each scheduled time point.

12. ADVERSE EVENT REPORTING

Timely, accurate, and complete reporting and analysis of safety information from clinical studies are crucial for the protection of subjects, investigators, and the sponsor, and are mandated by regulatory agencies worldwide. The sponsor has established Standard Operating Procedures in conformity with regulatory requirements worldwide to ensure appropriate reporting of safety information; all clinical studies conducted by the sponsor or its affiliates will be conducted in accordance with those procedures.

12.1. Definitions

12.1.1. Adverse Event Definitions and Classifications

- **Adverse Event**
  An adverse event is any untoward medical occurrence in a clinical study subject administered a pharmaceutical product. An adverse event does not necessarily have a causal relationship with the treatment. An adverse event can therefore be any unfavorable and unintended sign (including an abnormal finding), symptom, or disease temporally associated with the use of a medicinal (investigational) product, whether or not related to the medicinal (investigational) product. (Definition per International Conference on Harmonisation [ICH])

  This includes any occurrence that is new in onset or aggravated in severity or frequency from the baseline condition, or abnormal results of diagnostic procedures, including laboratory test abnormalities.

  Note: The sponsor collects adverse events starting with the signing of the informed consent.

- **Serious Adverse Event**
  A serious adverse event as defined by ICH is any untoward medical occurrence that at any dose meets any of the following conditions:
  - results in death
  - is life-threatening
    (The subject was at risk of death at the time of the event. It does not refer to an event that hypothetically might have caused death if it were more severe.)
  - requires inpatient hospitalization or prolongation of existing hospitalization
  - results in persistent or significant disability/incapacity, or
  - is a congenital anomaly/birth defect

  Note: Medical and scientific judgment should be exercised in deciding whether expedited reporting is also appropriate in situations other than those listed above. For example, important medical events may not be immediately life threatening or result in death or hospitalization, but may jeopardize the subject or may require intervention to prevent one of the outcomes listed in the definition above. Any adverse event is considered a serious adverse event if it is associated with clinical signs or symptoms judged by the investigator to have a significant clinical impact.

- **Unlisted (Unexpected) Adverse Event**
  An unlisted adverse event, the nature or severity of which is not consistent with the applicable product information. For an investigational product, the expectedness of an adverse event will be determined by

whether or not it is listed in the Investigator's Brochure. For a comparator product with a marketing authorization, the expectedness of an adverse event will be determined by whether or not it is listed in the United Kingdom Summary of Product Characteristics (U.K. SmPC).

- **Associated With the Use of the Drug**
  An adverse event is considered associated with the use of the drug if the attribution is possible, probable, or very likely by the definitions listed in Section 12.1.2.

12.1.2. Attribution Definitions

- **Not related**
  An adverse event which is not related to the use of the drug.

- **Doubtful**
  An adverse event for which an alternative explanation is more likely, e.g., concomitant drug(s), concomitant disease(s), or the relationship in time suggests that a causal relationship is unlikely.

- **Possible**
  An adverse event which might be due to the use of the drug. An alternative explanation, e.g., concomitant drug(s), concomitant disease(s), is inconclusive. The relationship in time is reasonable; therefore, the causal relationship cannot be excluded.

- **Probable**
  An adverse event which might be due to the use of the drug. The relationship in time is suggestive (e.g., confirmed by dechallenge). An alternative explanation is less likely, e.g., concomitant drug(s), concomitant disease(s).

- **Very likely**
  An adverse event which is listed as a possible adverse reaction and cannot be reasonably explained by an alternative explanation, e.g., concomitant drug(s), concomitant disease(s). The relationship in time is very suggestive (e.g., it is confirmed by dechallenge and rechallenge).

12.2. Procedures

12.2.1. All Adverse Events

All adverse events will be reported from the time a signed and dated informed consent form is obtained until completion of the last study-related procedure (LFU visit [telephone contact]) will be reported. Progression of the disease under study (hospitalized CAP) will not constitute an adverse event (lack of efficacy or insufficient clinical response should not be recorded as an adverse event). Those meeting the definition of serious adverse events must be reported using the Serious Adverse Event Form, including serious adverse events spontaneously reported to the investigator

within 30 days after the subject has completed the study (including poststudy follow up). The sponsor will evaluate any safety information that is spontaneously reported by an investigator beyond the time frame specified in the protocol.

All adverse events, regardless of seriousness, severity, or presumed relationship to study therapy, must be recorded using medical terminology in the source document and the CRF. Whenever possible, diagnoses should be given when signs and symptoms are due to a common etiology (e.g., cough, runny nose, sneezing, sore throat, and head congestion should be reported as "upper respiratory infection"). Investigators must record in the CRF their opinion concerning the relationship of the adverse event to study therapy. All measures required for adverse event management must be recorded in the source document and reported according to sponsor instructions.

The sponsor assumes responsibility for appropriate reporting of adverse events to the regulatory authorities. The sponsor will also report to the investigator all serious adverse events that are unlisted and associated with the use of the drug. The investigator (or sponsor where required) must report these events to the appropriate Independent Ethics Committee/Institutional Review Board (IEC/IRB) that approved the protocol unless otherwise required and documented by the IEC/IRB.

Subjects (or their designees, if appropriate) must be provided with a "study card" indicating the name of the investigational product, the study number, the investigator's name, a 24-hour emergency contact number, and, if applicable, excluded concomitant medications.

12.2.2. Serious Adverse Events

All serious adverse events occurring during clinical studies must be reported to the appropriate sponsor contact person by investigational staff within 24 hours of their knowledge of the event.

Information regarding serious adverse events will be transmitted to the sponsor using the Serious Adverse Event Form, which must be signed by a member of the investigational staff. The initial report of a serious adverse event may be made by facsimile (fax) or telephone. It is preferable that serious adverse events be reported via fax. After a telephone report of a

serious adverse event, a Serious Adverse Event Form must be completed by the investigational staff and transmitted to the sponsor within 1 working day.

All serious adverse events that have not resolved by the end of the study, or that have not resolved upon discontinuation of the subject's participation in the study, must be followed until any of the following occurs:

- the event resolves
- the event stabilizes
- the event returns to baseline, if a baseline value is available
- the event can be attributed to agents other than the study drug or to factors unrelated to study conduct
- when it becomes unlikely that any additional information can be obtained (subject or health care practitioner refusal to provide additional information, lost to follow-up after demonstration of due diligence with follow-up efforts)

The cause of death of a subject in a clinical study, whether or not the event is expected or associated with the investigational agent, is considered a serious adverse event. Any event requiring hospitalization (or prolongation of hospitalization) that occurs during the course of a subject's participation in a clinical study must be reported as a serious adverse event, except hospitalizations for:

- initial admission to the hospital for this episode of pneumonia
- social reasons in absence of an adverse event
- surgery or procedure planned before entry into the study (must be documented in the CRF)

12.2.3. Pregnancies

Subject pregnancy must be reported by the investigational staff within 1 working day of their knowledge of the event using the pregnancy notification form. Any subject who becomes pregnant during the study must be promptly withdrawn from the study.

Follow-up information regarding the outcome of the pregnancy and any postnatal sequelae in the infant will be required.

12.3. Contacting Sponsor Regarding Safety

The names of the individuals (and corresponding phone numbers) who should be contacted regarding safety issues or questions regarding the study

are listed on the Contact Information page(s), which will be provided as a separate document.

13. STUDY DRUG INFORMATION

13.1. Physical Description of Study Drug(s)

The study drugs consist of ceftobiprole medocaril 500 mg, ceftriaxone 2 g, linezolid 600 mg, and cefuroxime axetil 500 mg, and all will be provided by the sponsor.

Ceftobiprole is supplied in 10 mL vials containing 500 mg of lyophilized powdered. The powder is white to slightly yellowish or brownish in appearance.

Ceftriaxone is a white to yellowish-orange crystalline powder, which is readily soluble in water, sparingly soluble in methanol and very slightly soluble in ethanol.

Linezolid is supplied in single-use, ready-to-use infusion bags, as a sterile isotonic solution for i.v. infusion. Each mL contains 2 mg of linezolid. Inactive ingredients are sodium citrate, citric acid, and dextrose in an aqueous vehicle for i.v. administration.

Cefuroxime axetil is supplied as capsule-shaped, film-coated tablets.

13.2. Packaging

Ceftobiprole will be supplied as vials of sterile lyophilized ceftobiprole medocaril containing 500 mg for i.v. administration.

Ceftriaxone will be supplied as vials containing 2 g of ceftriaxone sodium.

Study sites will provide placebo infusion solution.

The oral study drug (cefuroxime axetil) will be provided to the investigator sites in commercial packages labeled for use in this clinical trial.

Linezolid will be supplied in single-use, ready to use 300 mL infusion bags containing 600 mg linezolid.

13.3. Labeling

Study drug labels will contain information to meet the applicable regulatory requirements.

13.4. Preparation and Handling

Ceftobiprole, linezolid, ceftriaxone, and cefuroxime axetil will be administered according to instructions in the protocol. All 3 i.v. study drugs will be diluted in 5% dextrose (glucose) solution.

Preparation and administration of ceftobiprole and ceftriaxone

After reconstituting 500 mg ceftobiprole medocaril lyophilizate in the reconstitution solution provided (10 mL of reconstitution solution for the 500 mg vials), the resultant solution must be further diluted with 250 mL 5% dextrose (glucose) for infusion. The reconstituted solution should immediately (within 1 hour of preparation) be diluted in 5% dextrose infusion solution. Solutions of ceftobiprole medocaril should not be frozen. The ceftobiprole lyophilizate must be stored in a refrigerator between 2°C to 8°C. The reconstitution solution can be stored at ambient temperature. Ceftobiprole must be protected from direct sunlight (lyophilizate as well as solution). Solutions containing NaCl must not be administered through the same infusion line as ceftobiprole. Further details of the preparation and administration of ceftobiprole are presented in Attachment 2, Preparation and Administration of Intravenous Infusion Solutions of Ceftobiprole Medocaril (500 mg dose).

Ceftriaxone sterile powder must be stored at room temperature (25°C) or below and protected from light. Powder (2 g/vial) will be reconstituted using 5% dextrose (glucose) solution and be prepared for i.v. administration at a final dilution of 2 g/50 mL (40 mg/mL). This 50 mL solution will be infused over 30 minutes.

Linezolid infusion bags should be stored at room temperature and protected from light. Infusion bags should not be frozen.

Refer to Section 6, Dosage and Administration, for additional information.

Preparation and administration of ceftobiprole for severe renal impaired subjects

After reconstituting 500 mg ceftobiprole medocaril lyophilizate in the reconstitution solution provided (10 mL of reconstitution solution for the 500 mg vials), only 5 mL of resultant solution must be further diluted with 250 mL 5% dextrose (glucose) for infusion for administering 250 mg dose.

The reconstituted solution should immediately (within 1 hour of preparation) be diluted in 5% dextrose infusion solution. Solutions of ceftobiprole medocaril should not be frozen. The ceftobiprole lyophilizate must be stored in a refrigerator between 2°C to 8°C. The reconstitution solution can be stored at ambient temperature. Ceftobiprole must be protected from direct sunlight (lyophilizate as well as solution). Solutions containing NaCl must not be administered through the same infusion line as ceftobiprole. Further details of the preparation and administration of ceftobiprole are presented in Attachment 2, Preparation and Administration of Intravenous Infusion Solutions of Ceftobiprole Medocaril (500 mg dose).

13.5. Drug Accountability

An unblinded monitor will perform drug accountability. The clinical investigator is responsible for ensuring that all study drug received at the site is inventoried and accounted for throughout the study. The dispensing of study drug to the subject, and the return of study drug from the subject (if applicable), must be documented on the drug accountability form. Subjects or their legally acceptable representative must be instructed to return all original containers, whether empty or containing study drug. Study drug returned by study subjects will be stored and disposed of according to the sponsor's instructions. Contents of the study drug containers must not be combined.

Study drug must be handled strictly in accordance with the protocol and the container label and will be stored in a limited access area or in a locked cabinet under appropriate environmental conditions. Unused study drug and study drug returned by the subject must be available for verification by the sponsor's site monitor during on-site monitoring visits. The return to the sponsor of unused study drug, or used returned study drug for destruction, will be documented on the Drug Return Form.

Study drug should be dispensed under the supervision of the investigator, a qualified member of the investigational staff, or by a hospital/clinic pharmacist. Study drug will be supplied only to subjects participating in the study. Returned study drug must not be dispensed again, even to the same subject. Study drug may not be relabeled or reassigned for use by other subjects. The investigator agrees neither to dispense the study drug from, nor store it at, any site other than the study sites agreed upon with the sponsor.

14. STUDY-SPECIFIC MATERIALS

The investigator will be provided with the following supplies:

- United Kingdom summary of product characteristics for ceftriaxone, linezolid, and cefuroxime axetil
- Investigator's Brochure for ceftobiprole
- A separate laboratory manual for the collection, handling, and shipping of specimens
- E-test and discs for susceptibility testing to ceftobiprole
- Urine antigen test kit for *L. pneumophila*
- Pharmacokinetic laboratory manual

15. ETHICAL ASPECTS

15.1. Study-Specific Design Considerations

There are 5 aspects of this study design requiring ethical justification.

Surveillance studies of susceptibility patterns of microorganisms known to cause CAP suggest that both study regimens (ceftobiprole/placebo or ceftriaxone/linezolid) should be highly efficacious in treating such infections. The comparator regimen consists of approved antibiotics. Ceftriaxone is commonly recommended to treat CAP. Linezolid is included initially to provide treatment for resistant pathogens (e.g., PRSP, MRSA) that are included in the spectrum of activity of ceftobiprole. Once these resistant pathogens are ruled out as the cause for CAP, the linezolid will be discontinued in order to decrease exposure of subjects to unnecessary antibiotics. However neither treatment regimen is active against *Legionella* spp, which are uncommon causes of CAP but have the potential to cause severe, even fatal disease. An overwhelming number of cases of CAP due to *Legionella* spp are caused by *L. pneumophila*. The protocol excludes subjects with CAP known or suspected to be caused by *Legionella* spp and mandates a urine test for legionella antigen that is highly specific and sensitive.

The second issue is the selected dose of ceftobiprole. The PK modeling data of ceftobiprole suggest that the current dosing regimen should be highly efficacious in treating CAP due to *S. pneumoniae* (including penicillin and ceftriaxone-resistant strains), *S. aureus* (including MRSA and VRSA), *H. influenzae,* and the majority of strains of enteric gram-negative rods (e.g., *K. pneumoniae*).

The third issue is that there are no efficacy data in the use of ceftobiprole for treatment of pulmonary infections or infections due to *S. pneumoniae*. The only data in subjects is from the Phase 2 studies in complicated skin infections and used a different dose of ceftobiprole. However, in vitro and animal studies of ceftobiprole demonstrate that it behaves in a similar fashion to other cephalosporins. Cephalosporins have been used as first line therapy for CAP for over 2 decades and it is anticipated that ceftobiprole will be equally efficacious. Additionally, an IDMC will assess the efficacy data in an ongoing fashion. The same IDMC will also review efficacy data from the 2 ongoing Phase 3 studies in nosocomial pneumonia.

The fourth issue is that the safety profile of ceftobiprole is not fully established, and therefore subjects may be placing themselves at increased risk by enrolling in the study. At this point, there are no indications from the Phase 1 and 2 studies that the safety profile of ceftobiprole will be significantly worse than the ceftriaxone/linezolid comparator. The adverse event profile from Phase 1 and 2 studies of ceftobiprole suggest that the main adverse events are nausea and vomiting. Subject safety will be closely monitored throughout the study. In all cases, the investigator will receive detailed information about the study in order to insure that those individuals involved are fully informed. The IDMC will monitor safety (as well as efficacy) data on an ongoing basis to ensure the continuing safety of subjects enrolled in this study and also in the 2 ongoing Phase 3 studies in nosocomial pneumonia.

Finally, the fifth ethical issue in the current study relates to the consenting of a critically ill subject. In cases where the subject in not incapacitated, consent will be obtained directly from the subject though normal procedures of obtaining informed consent. In cases where a subject is incapacitated or becomes incapacitated during the conduct of the study, permission to either participate in the study or continue participation (in subjects already enrolled) will be provided by a legally authorized representative. When a subject is no longer incapacitated, informed consent will be obtained from the subject at that time.

15.2. Regulatory Ethics Compliance

15.2.1. Investigator Responsibilities

The investigator is responsible for ensuring that the clinical study is performed in accordance with the protocol, current ICH guidelines on Good Clinical Practice (GCP), and applicable regulatory requirements.

Good Clinical Practice is an international ethical and scientific quality standard for designing, conducting, recording, and reporting studies that involve the participation of human subjects. Compliance with this standard provides public assurance that the rights, safety, and well being of study subjects are protected, consistent with the principles that originated in the Declaration of Helsinki, and that the clinical study data are credible.

15.2.2. Independent Ethics Committee or Institutional Review Board

Before the start of the study, the investigator (or sponsor where required) will provide the IEC/IRB with current and complete copies of the following documents:

- final protocol and, if applicable, amendments
- sponsor-approved informed consent form (and any other written materials to be provided to the subjects)
- Investigator's Brochure (or equivalent information) and amendments
- sponsor-approved subject recruiting materials
- information on compensation for study-related injuries or payment to subjects for participation in the study, if applicable
- investigator's curriculum vitae or equivalent information (unless not required, as documented by IEC/IRB)
- information regarding funding, name of the sponsor, institutional affiliations, other potential conflicts of interest, and incentives for subjects
- any other documents that the IEC/IRB requests to fulfill its obligation

This study will be undertaken only after IEC/IRB has given full approval of the final protocol, amendments (if any), the informed consent form, applicable recruiting materials, and subject compensation programs, and the sponsor has received a copy of this approval. This approval letter must be dated and must clearly identify the documents being approved.

During the study the investigator (or sponsor where required) will send the following documents to the IEC/IRB for their review and approval, where appropriate:

- protocol amendments
- revision(s) to informed consent form and any other written materials to be provided to subjects
- if applicable, new or revised subject recruiting materials approved by the sponsor
- revisions to compensation for study-related injuries or payment to subjects for participation in the study, if applicable
- Investigator's Brochure amendments or new edition(s)
- summaries of the status of the study (at least annually or at intervals stipulated in guidelines of the IEC/IRB)
- reports of adverse events that are serious, unlisted, and associated with the investigational drug
- new information that may adversely affect the safety of the subjects or the conduct of the study
- deviations from or changes to the protocol to eliminate immediate hazards to the subjects
- report of deaths of subjects under the investigator's care
- notification if a new investigator is responsible for the study at the site
- any other requirements of the IEC/IRB

For protocol amendments that increase subject risk, the amendment and applicable informed consent form revisions must be submitted promptly to the IEC/IRB for review and approval before implementation of the change(s).

At least once a year, the IEC/IRB will be asked to review and reapprove this clinical study. This request should be documented in writing.

At the end of the study, the investigator (or sponsor where required) will notify the IEC/IRB about the study completion.

15.2.3. Informed Consent

Each subject (or a legally acceptable representative) must give written consent according to local requirements after the nature of the study has been fully explained. The consent form must be signed before performance of any

study-related activity. The consent form and assent form that is used must be approved by both the sponsor and by the reviewing IEC/IRB. The informed consent should be in accordance with principles that originated in the Declaration of Helsinki, current ICH and GCP guidelines, applicable regulatory requirements, and sponsor policy.

Before entry into the study, the investigator or an authorized member of the investigational staff must explain to potential subjects or their legally acceptable representatives the aims, methods, reasonably anticipated benefits, and potential hazards of the study, and any discomfort it may entail. Subjects will be informed that their participation is voluntary and that they may withdraw consent to participate at any time. They will be informed that choosing not to participate will not affect the care the subject will receive for the treatment of his/her disease. Finally, they will be told that the investigator will maintain a subject identification register for the purposes of long-term follow-up if needed and that their records may be accessed by health authorities and authorized sponsor staff without violating the confidentiality of the subject, to the extent permitted by the applicable law(s) or regulations. By signing the informed consent form the subject or legally acceptable representative is authorizing such access, and agrees to be recontacted after study completion, by health authorities and authorized sponsor staff, for the purpose of obtaining consent for additional safety evaluations if needed.

The subject or legally acceptable representative will be given sufficient time to read the informed consent form and the opportunity to ask questions. After this explanation and before entry into the study, consent should be appropriately recorded by means of either the subject's or his/her legally acceptable representative's dated signature. After having obtained the consent, a copy of the informed consent form must be given to the subject.

If the subject or legally acceptable representative is unable to read or write, an impartial witness should be present for the entire informed consent process (which includes reading and explaining all written information) and should personally date and sign the informed consent form after the oral consent of the subject or legally acceptable representative is obtained.

When prior consent of the subject is not possible and the subject's legally acceptable representative is not available, enrollment procedures should be described in the protocol with documented approval/favorable opinion by the

IEC/IRB to protect the rights, safety, and well being of the subject and to ensure compliance with applicable regulatory requirements. The subject or legally acceptable representative should be informed about the study as soon as possible and give consent to continue.

15.2.4. Privacy of Personal Data

The collection and processing of personal data from subjects enrolled in this study will be limited to those data that are necessary to investigate the efficacy, safety, quality, and utility of the investigational product(s) used in this study.

These data must be collected and processed with adequate precautions to ensure confidentiality and compliance with applicable data privacy protection laws and regulations.

The sponsor ensures that the personal data will be

- processed fairly and lawfully
- collected for specified, explicit, and legitimate purposes and not further processed in a way incompatible with these purposes
- adequate, relevant, and not excessive in relation to said purposes
- accurate and, where necessary, kept current

Explicit consent for the processing of personal data will be obtained from the participating subject (or his/her legally acceptable representative) before collection of data. Such consent should also address the transfer of the data to other entities and to other countries.

The subject has the right to request through the investigator access to his/her personal data and the right to request rectification of any data that are not correct or complete. Reasonable steps should be taken to respond to such a request, taking into consideration the nature of the request, the conditions of the study, and the applicable laws and regulations.

Appropriate technical and organizational measures to protect the personal data against unauthorized disclosures or access, accidental or unlawful destruction, or accidental loss or alteration must be put in place. Sponsor personnel whose responsibilities require access to personal data agree to keep the identity of study subjects confidential.

16. ADMINISTRATIVE REQUIREMENTS

16.1. Protocol Modifications

Neither the investigator nor the sponsor will modify this protocol without a formal amendment. All protocol amendments must be issued by the sponsor, and signed and dated by the investigator. Protocol amendments must not be implemented without prior IEC/IRB approval, or when the relevant competent authority has raised any grounds for non-acceptance, except when necessary to eliminate immediate hazards to the subjects, in which case the amendment must be promptly submitted to the IEC/IRB and relevant competent authority. When the change(s) involves only logistic or administrative aspects of the study, the IRB (and IEC where required) only needs to be notified.

In situations requiring a departure from the protocol, the investigator or other physician in attendance will contact the appropriate sponsor representative by fax or telephone (see Contact Information pages provided separately). If possible, this contact will be made before implementing any departure from the protocol. In all cases, contact with the sponsor must be made as soon as possible in order to discuss the situation and agree on an appropriate course of action. The data recorded in the CRF and source document will reflect any departure from the protocol, and the source documents will describe this departure and the circumstances requiring it.

16.2. Regulatory Documentation

16.2.1. Regulatory Approval/Notification

This protocol and any amendment(s) must be submitted to the appropriate regulatory authorities in each respective country, if applicable. A study may not be initiated until all local regulatory requirements are met.

16.2.2. Required Prestudy Documentation

The following documents must be provided to the sponsor before shipment of study drug to the investigational site:

- protocol and amendment(s), if any, signed and dated by the investigator
- a copy of the dated and signed written IEC/IRB approval of the protocol, amendments, informed consent form, any recruiting materials, and if applicable, subject compensation programs. This approval must clearly identify the specific protocol by title and number and must be signed by the chairman or authorized designee.

- name and address of the IEC/IRB including a current list of the IEC/IRB members and their function, with a statement that it is organized and operates according to GCP and the applicable laws and regulations. If accompanied by a letter of explanation from the IEC/IRB, a general statement may be substituted for this list. If an investigator or a member of the investigational staff is a member of the IEC/IRB, documentation must be obtained to state that this person did not participate in the deliberations or in the vote/opinion of the study.
- regulatory authority approval or notification, if applicable
- signed and dated statement of investigator (e.g., Form FDA 1572), if applicable
- documentation of investigator qualifications (e.g., curriculum vitae)
- completed investigator financial disclosure form from the investigator
- signed and dated clinical trial agreement, which includes the financial agreement
- any other documentation required by local regulations

The following documents must be provided to the sponsor before enrollment of the first subject:

- completed investigator financial disclosure forms from all subinvestigators
- documentation of subinvestigator qualifications (e.g., curriculum vitae)
- photocopy of the site signature log, describing delegation of roles and responsibilities at the start of the study
- name and address of any local laboratory conducting tests for the study, and a dated copy of current laboratory normal ranges for these tests
- local laboratory documentation demonstrating competence and test reliability (e.g., accreditation/license), if applicable.

16.3. Subject Identification Register and Subject Screening Log

The investigator agrees to complete a subject identification register to permit easy identification of each subject during and after the study. This document will be reviewed by the sponsor site contact for completeness.

The subject identification register will be treated as confidential and will be filed by the investigator in the trial center file. To ensure subject confidentiality, no copy will be made. All reports and communications relating to the study will identify subjects by initials and assigned number only.

The investigator must also complete a subject screening log, which reports on all subjects who were seen to determine eligibility for inclusion in the study.

16.4. Case Report Form Completion

Case report forms are provided for each subject in printed or electronic format.

Electronic Data Capture (EDC) will be used for this study. The majority of the study data will be transcribed by study personnel from the source documents onto an electronic CRF and transmitted in a secure manner to the sponsor. Worksheets may be provided for the capture of some data for easier transfer to the electronic CRF. The electronic file will be considered as the CRF.

All data relating to the study must be recorded in CRFs prepared by the sponsor. Data must be entered into CRFs in English. The CRFs are to be completed at the time of the subject's visit, with the exception of results of tests performed outside the investigator's office, so that they always reflect the latest observations on the subjects participating in the study.

The investigator must verify that all data entries in the CRFs are accurate and correct.

All CRF entries, corrections, and alterations must be made by the investigator or other authorized study-site personnel. The correct data must be inserted, dated, and initialed by the investigator or an authorized member of the investigational staff. Completed CRFs will be submitted according to the sponsor's instructions and reviewed by the sponsor to determine their acceptability.

16.5. Data Quality Assurance

Steps to be taken to ensure the accuracy and reliability of data include the selection of qualified investigators and appropriate study centers, review of protocol procedures with the investigator and associated personnel before the study, periodic monitoring visits by the sponsor, and direct transmission of clinical laboratory data from a central laboratory into the sponsor's data base. Written instructions will be provided for collection, preparation, and shipment of blood, plasma, and urine samples. CRF completion guidelines

will be provided and reviewed with study personnel before the start of the study. The sponsor will review CRFs for accuracy and completeness during on-site monitoring visits and after their return to the sponsor; any discrepancies will be resolved with the investigator or designee, as appropriate. The data will be entered into the clinical study database and verified for accuracy.

16.6. Record Retention

In compliance with the ICH/GCP guidelines, the investigator/institution will maintain all CRFs and all source documents that support the data collected from each subject, as well as all study documents as specified in ICH/GCP Section 8, Essential Documents for the Conduct of a Clinical Trial, and all study documents as specified by the applicable regulatory requirement(s). The investigator/institution will take measures to prevent accidental or premature destruction of these documents.

Essential documents must be retained until at least 2 years after the last approval of a marketing application in an ICH region and until there are no pending or contemplated marketing applications in an ICH region or until at least 2 years have elapsed since the formal discontinuation of clinical development of the investigational product. These documents will be retained for a longer period if required by the applicable regulatory requirements or by an agreement with the sponsor. It is the responsibility of the sponsor to inform the investigator/institution as to when these documents no longer need to be retained.

If the responsible investigator retires, relocates, or for other reasons withdraws from the responsibility of keeping the study records, custody must be transferred to a person who will accept the responsibility. The sponsor must be notified in writing of the name and address of the new custodian. Under no circumstance shall the investigator relocate or dispose of any study documents before having obtained written approval from the sponsor.

For CRFs completed on NCR paper, one copy is to be retained in the archives of the sponsor from the country in which the study is performed. A second copy must be archived by the investigator.

If it becomes necessary for the sponsor or the appropriate regulatory authority to review any documentation relating to this study, the investigator must permit access to such reports.

16.7. Monitoring

The sponsor will perform on-site monitoring visits as frequently as necessary. The monitor will record dates of the visits in a study center visit log that will be kept at the site. The first post-initiation visit will usually be made as soon as possible after enrollment has begun. At these visits, the monitor will compare the data entered into the CRFs with the hospital or clinic records (source documents). The nature and location of all source documents will be identified to ensure that all sources of original data required to complete the CRF are known to the sponsor and investigational staff and are accessible for verification by the sponsor site contact. If electronic records are maintained at the investigational site, the method of verification must be discussed with the investigational staff. At a minimum, source documentation must be available to substantiate: subject identification, eligibility, and participation; proper informed consent procedures; dates of visits; adherence to protocol procedures; records of safety and efficacy parameters; adequate reporting and follow-up of adverse events; administration of concomitant medication; drug receipt/dispensing/ return records; study drug administration information; and date of subject completion, discontinuation from treatment, or withdrawal from the study, and the reason if appropriate. Specific items required as source documents will be reviewed with the investigator before the study.

If data are recorded directly into the CRF, at a minimum there should be an entry in the medical record that each of the assessments was done, and by whom and the date it was done. It is recommended that the author of an entry in the source documents be identifiable.

Direct access to source documentation (medical records) must be allowed for the purpose of verifying that the data recorded in the CRF are consistent with the original source data. Findings from this review of CRFs and source documents will be discussed with the investigational staff. The sponsor expects that, during monitoring visits, the relevant investigational staff will be available, the source documentation will be available, and a suitable environment will be provided for review of study-related documents. The

monitor will meet with the investigator on a regular basis during the study to provide feedback on the study conduct.

If corrections to a CRF are needed after removal of the original CRF copy from the investigational site, a DCF will be used.

16.8. Study Completion/Termination

16.8.1. Study Completion

The study is considered completed with the last visit of the last subject undergoing the study. The final data from the investigational site will be sent to the sponsor (or designee) no more than 48 hours following completion of the final subject visit at that site.

16.8.2. Study Termination

The sponsor reserves the right to close the investigational site or terminate the study at any time. Investigational sites will be closed upon study completion. An investigational site is considered closed when all required documents and study supplies have been collected and a site closure visit has been performed.

The investigator may initiate site closure at any time, provided there is reasonable cause and sufficient notice is given in advance of the intended termination.

Reasons for the early closure of an investigational site by the sponsor or investigator, or termination of a study by the sponsor, may include but are not limited to:

- failure of the investigator to comply with the protocol, the sponsor's procedures, or GCP guidelines
- safety concerns
- sufficient data suggesting lack of efficacy
- inadequate recruitment of subjects by the investigator

16.9. On-Site Audits

Representatives of the sponsor's clinical quality assurance department may visit the site to carry out an audit of the study in compliance with regulatory guidelines and company policy. These audits will require access to all study records, including source documents, for inspection and comparison with the CRFs. Subject privacy must, however, be respected.

Similar auditing procedures may also be conducted by agents of any regulatory body reviewing the results of this study in support of a regulatory submission. The investigator should immediately notify the sponsor if they have been contacted by a regulatory agency concerning an upcoming inspection.

16.10. Use of Information and Publication

All information, including but not limited to information regarding ceftobiprole or the sponsor's operations (e.g., patent application, formulas, manufacturing processes, basic scientific data, prior clinical data, and formulation information) supplied by the sponsor to the investigator and not previously published, and any data generated as a result of this study, are considered confidential and remains the sole property of the sponsor. The investigator agrees to maintain this information in confidence and use this information only to accomplish this study, and will not use it for other purposes without the sponsor's prior written consent.

The investigator understands that the information developed in the clinical study will be used by the sponsor in connection with the continued development of ceftobiprole, and thus may be disclosed as required to other clinical investigators or regulatory agencies. To permit the information derived from the clinical studies to be used, the investigator is obligated to provide the sponsor with all data obtained in the study.

The results of the study will be reported in a Clinical Study Report generated by the sponsor and will contain all data from all investigational sites. Any work created in connection with performance of the study and contained in the data that can benefit from copyright protection (except any publication by the investigator as provided for below) shall be the property of the sponsor as author and owner of copyright in such work.

Sponsor shall have the right to publish such data and information without approval from the investigator. If an investigator wishes to publish information from the study, a copy of the manuscript must be provided to the sponsor for review at least 60 days before submission for publication or presentation. Expedited reviews will be arranged for abstracts, poster presentations, or other materials. If requested by the sponsor in writing, the investigator will withhold such publication for up to an additional 60 days to allow for filing of a patent application. In the event that issues arise

regarding scientific integrity or regulatory compliance, the sponsor will review these issues with the investigator. The sponsor will not mandate modifications to scientific content and does not have the right to suppress information. The investigator will recognize the integrity of a multicenter study by not publishing data derived from the individual site until the combined results from the completed study have been published in full, within 12 months after conclusion, abandonment, or termination of the study at all sites, or the sponsor confirms there will be no multicenter study publication. Authorship of publications resulting from this study will be based on generally accepted criteria for major medical journals.

17. REFERENCES*

1. Mandell L, Bartlett J, Dowell S, et al. Update of practice guidelines for the management of community-acquired pneumonia in immunocompetent adults. Clin Infect Dis 2003;37:1405-1433.

2. File T, Garau J, Blasi F, et al. Guidelines for empiric antimicrobial prescribing in community-acquired pneumonia. Chest 2004;125:1888-1901.

3. Davies T, Bush K. Activity of BAL9141 and other β-lactams against *Streptococcus pneumoniae* in U.S. clinical isolates with defined substitutions in penicillin binding proteins (PBP) PBP1a, PBP2b, and PBP2x. Antimicrob Agents Chemother 2006 (in press).

4. Ceftobiprole Medocaril (JNJ-30982081, BAL5788); Johnson & Johnson Pharmaceutical Research & Development Investigator's Brochure, Edition 5.0; Document ID No: EDMS-PSDB-4316052 (30 November 2005).

5. Hebeisen P, Heinze-Krauss I, Angehrn P, et al. In vitro and in vivo properties of Ro63-9141, a novel broad-spectrum cephalosporin with activity against methicillin-resistant staphylococci. Antimicrob Agents Chemother 2001;45:825-836.

6. Deshpande LM, Jones RN, Biedenbach DJ, Beach ML, Antimicrobial potency and spectrum for Ro63-9141 (RO), a novel cephalosporin with activity against methicillin-resistant staphylococci (MRS). Abstracts of the 41st Interscience Conference on Antimicrobial Agents and Chemotherapy, September and December 2001: Poster F-375, page 211.

7. Entenza JM, Hohl P, Heinze-Krauss I, et al. BAL9141, a novel extended-spectrum cephalosporin active against methicillin-resistant *Staphylococcus aureus* in treatment of experimental endocarditis. Antimicrob Agents Chemother 2002;46:171-177.

8. Jones RN, Lalitagauri LM, Mutnick AH, Biedenbach DJ. In vitro evaluation of BAL9141, a novel parenteral cephalosporin active against oxacillin-resistant staphylococci. J Antimicrob Chemother 2002;50:915-932.

9. Rocephin® [package insert]. Nutley, NJ: Roche Laboratories, Inc.; 2004.

10. Zyvox® [package insert]. Kalamazoo, MI: Pharmacia & UpJohn; 2001.

11. Ceftin® (cefuroxime axetil) [package insert]. New York, NY: GlaxoSmithKline; 2003

12. Mandell, LA. Epidemiology and etiology of community-acquired pneumonia. Infect Dis Clin North Am 2004;18:761-776.

13. Fine MJ, Auble TE, Yealy DM, et al. A prediction rule to identify low-risk patients with community-acquired pneumonia. N Engl J Med 1997;336:243-250.

14. Ginesu F, Pirana P, Deiola G, et al. Etiology and therapy of community-acquired pneumonia. J Chemother 1997;9:285-292.

* This section of the protocol has been revised. Please refer to the section of this document entitled PROTOCOL AMENDMENTS (Amendment INT-3, 22 December 2006) for a detailed description of the specific changes.

15. Ewig S, Ruiz M, Mensa J, et al. Severe community-acquired pneumonia. Assessment of severity criteria. Am J Respir Crit Care Med 1998;158:1102-1108.

16. Reynolds H. Respiratory infections: Community-acquired pneumonia and newer microbes. Lung 1996;174:207-224.

17. So H. Severe community-acquired pneumonia. Anaesth Intensive Care 1997;25:222-234.

18. Marie T. Community-acquired pneumonia: Epidemiology, etiology, treatment. Infect Dis Clin North Am 1998;12:723.

19. Ewig S, Torres A. Severe community-acquired pneumococcal pneumonia-what might be done better. Intensive Care Med 1999;25:143-145.

20. Torres A, Serra-Batlles J, Ferrer A, et al. Severe community-acquired pneumonia. Epidemiology and prognostic factors. Am Rev Respir Dis 1991;144:312-318.

21. Pachon J, Prados M, Capote F, et al. Severe community-acquired pneumonia: etiology, prognosis, and treatment. Am Rev Respir Dis 1990;142:369-373.

22. Baba T, Takeuchi F, Kuroda M, et al. Genome and virulence determinants of high virulence community-acquired MRSA. Lancet 2002;359:1819-1827.

23. Daum RS, Ito T, Hiramatsu K, et al. A novel methicillin-resistant cassette in community-acquired methicillin-resistant Staphylococcus aureus isolates of diverse genetic backgrounds. J Infect Dis 2002;186:1344-1347.

24. Naimi TS, LeDell KH, Como-Sabietti K, et al. Comparison of community-acquired and health care-associated methicillin-resistant Staphylococcus aureus infection; JAMA 2003;290:2976-2984.

25. Gillet Y, Issartel P, Fournet J-C, et al. Association between *Staphylococcus aureus* strains carrying gene for Pantone-Valentine leukocidin and highly lethal necrotizing pneumonia in young immunocompetent patients. Lancet 2002;359:753-759.

26. Gorak EJ, Yamada SM, Brown JD. Community-acquired methicillin-resistant Staphylococcus aureus in hospitalized adults and children without known risk factors. Clin Infect Dis 1999;29:801-802.

27. Dufour P, Gillet Y, Bes M, et al. Community-acquired methicillin-resistant Staphylococcus aureus infections in France: emergence of a single clone that produces Pantone-Valentine leukocidin. Clin Infect Dis 2002;35:819-924.

28. Mongkolrattanothai K, Boyle S, Kahana MD, Daum RS. Severe Staphylococcus aureus infections caused by clonally related community-acquired methicillin-susceptible and methicillin-resistant isolates. Clin Infect Dis 2003;15:1050–1058.

29. File M Jr. Community-associated methicillin-resistant Staphylococcus aureus: not only a cause of skin infections, also a new cause of pneumonia: editorial review. Curr Opin Infect Dis 2005;18(2):123-124.

30. Gillet Y, Issartel B, Vanhems P, et al. Association between Staphylococcus aureus strains carrying gene for Panton–Valentine leukocidin and highly lethal necrotizing pneumonia in young immunocompetent patients. Lancet 2002;359:753–759.

31. Peleg AY, Munchkhof WJ. Fatal necrotizing pneumonia due to community-acquired methicillin-resistant Staphylococcus aureus. Med J Aust 2004;181:228–229.

32. Francis JS, Doherty MC, Lopatin U, et al. Severe Community-onset pneumonia in healthy adults caused by methicillin-resistant Staphylococcus aureus carrying the Panton-Valentine leukocidin genes. Clin Infest Dis 2005;40:100–107.

33. Hageman JC, Francis J, Uyeki TM, et al. Emergence of methicillin-resistant Staphylococcus aureus as a cause of community-acquired pneumonia during the influenza season, 2003-2004. Program and abstracts of the 42nd Annual Meeting of the Infectious Diseases Society of America. Boston, 30 September–3 October 2004 [Abstract LB-8].

34. NOW® *Legionella* Urinary Antigen Test [product instructions]. Portland, ME: Binax, Inc.; 2003.

35. Stralin K, Kaltoft MS, Konradsen HB, et al. Comparison of two urinary antigen tests for establishment of pneumococcal etiology of adult community-acquired pneumonia. J Clin Microbiol 2004;42(8):3620-3625.

36. Drusano G. Personal communication 2005.

37. Wunderink RG, Rello J, Cammarata SK, et al. Linezolid versus vancomycin: Analysis of two double-blind studies of patients with methicillin-resistant *Staphylococcus aureus* nosocomial pneumonia. Chest 2003;124:1789-1797.

38. Blasi F. Atypical pathogens and respiratory tract infections. Eur Respir J 2004;24:171-181.

39. American Thoracic Society. Guidelines for the management of adults with community-acquired pneumonia. Am J Respir Crit Care Med 2001;163:1730-1754.

40. Thibodeau KP, Ziera AJ. Atypical pathogens and challenges in community-acquired pneumonia. Am Fam Physician 2004;69:1699-1706.

41. Helbig J, Uldam S, Bernander S, et al. Clinical utility of urinary antigen detection for diagnosis of community-acquired, travel-associated, and nosocomial legionnaire's disease. J. Clin Microbiol 2003;41(2):838-840.

42. Marston B, Plouffe J, File T, et al. Incidence of community acquired pneumonia requiring hospitalizations: results of a population-based active surveillance study in Ohio. Community-Based Pneumonia Incidence Study Group. Arch Intern Med 1997;157(15):1709-1718.

43. Stout JE, Yu VL. Legionellosis. N Engl J Med 1997;337(10):682-687.

44. Ramirez JA. Switch therapy in adult patients with pneumonia. Clin Pulm Med 1995;2:327-333.

45. Ramirez J, Bordon J. Early switch from intravenous to oral antibiotics in hospitalized patients with bacteremic Streptococcus pneumoniae community-acquired pneumonia. Arch Intern Med 2001;161:848-850.

46. Halm E, Fine M, Marrie T, et al. Time to clinical stability in patients hospitalized with community-acquired pneumonia; implications for practice guidelines. JAMA 1998;279:1452-1457.

47. The Criteria Committee of the New York Heart Association. Nomenclature and Criteria for Diagnosis of Disease of the Heart and Great Vessels. 9th ed. Boston, Mass: Little, Brown & Co.; 1994:253-256.

48. Niederman S, Mandell L, Anzueto A, et al. Guidelines for the management of adults with community-acquired pneumonia: Diagnosis assessment of severity, antimicrobial therapy, and prevention. Am J Respir Crit Care Med 2001;163:1730-1754.

49. Yzerman EPF, den Boer JW, Lettinga KD, et al. Sensitivity of three urinary antigen tests associated with clinical severity in a large outbreak of legionnaires' disease in the Netherlands. J Clin Microbiol 2002;40:3232-3236.

ATTACHMENTS

Attachment 1*:
Pneumonia Outcomes Research Trial (PORT) Severity Index (PSI)

Complete the following to determine the PSI:

Step 1: Answer the following questions	YES	NO
1. Is the subject more than 50 years old?	___	___
2. Does the subject have a history of any of the following?		
Neoplastic disease	___	___
Congestive heart failure	___	___
Cerebrovascular disease	___	___
Renal disease	___	___
Liver disease	___	___
3. Does the subject have any of the following abnormalities on physical examination?		
Altered mental status	___	___
Pulse ≥125 beats/minute	___	___
Respiratory rate ≥30/min	___	___
Systolic blood pressure <90 mmHg	___	___
Temperature <35°C or ≥40°C	___	___

If **all** of the above are "NO", the subject has a score of 0 and is in Class I.

If **any** of the above are "YES", proceed to Step 2 to assign to Class II – V.

* This section of the protocol has been revised. Please refer to the section of this document entitled PROTOCOL AMENDMENTS (Amendment INT-3, 22 December 2006) for a detailed description of the specific changes.

Step 2: Continue to caculate the PSI if any of the items above in step 1 are "YES".

For each true condition, assign the indicated points.		Points assigned
Age		
Male	age in years	____
Female	age in years minus 10	____
Nursing home resident	+10	____
Coexisting illnesses		
Neoplastic disease[a]	+30	____
Liver disease[b]	+20	____
Congestive heart failure[c]	+10	____
Cerebrovascular disease[d]	+10	____
Renal disease[e]	+10	____
Physical examination findings		
Altered mental status[f]	+20	____
Respiratory rate >30 breaths/min	+20	____
Systolic blood pressure <90 mmHg	+20	____
Temperature <35°C or >40°C	+15	____
Pulse >125 beats/min	+10	____
Laboratory and radiographic findings		
Arterial pH <7.35[g]	+30	____
BUN >30 mg/dL (11 mmol/L)	+20	____
Sodium <130 mmol/L	+20	____
Glucose >250 mg/dL (14 mmol/L)	+10	____

Attachment 1: (Continued)

Pneumonia Outcomes Research Trial (PORT) Severity Index (PSI)

Hematocrit <30%	+10	____
PaO^2 <60 mmHg[h]	+10	____
Pleural effusion	+10	____
**SUM TO OBTAIN PSI**		______________

[a] Any cancer except basal or squamous cell cancer of the skin that was active at the time of presentation or diagnosed within 1 year of presentation.

[b] Clinical or histologic diagnosis of cirrhosis or another form of chronic liver disease, such as chronic active hepatitis.

[c] Systolic or diastolic ventricular dysfunction documented by history, physical examination, and chest radiography, echocardiogram, multiple gated acquisition scan, or left ventriculogram.

[d] Clinical diagnosis of stroke or transient ischemic attack or stroke documented by magnetic resonance imaging or computed tomography.

[e] History of chronic renal disease or abnormal blood urea nitrogen and creatinine concentrations documented in the medical record.

[f] Disorientation with respect to person, place, or time that is not known to be chronic, stupor, or coma.

[g] Venous blood pH may be used if arterial blood pH is not available.

[h] If the oxygen saturation on pulse oximetry is greater than 90%, this may be used as an indication of a PaO^2 of greater than 60 mmHg if PaO^2 is not available.

<u>PSI Classification</u>

PSI = 0	Class I (see Step 1)
PSI ≤ 70	Class II
PSI 71-90	Class III
PSI 91-130	Class IV
PSI >130	Class V

Subjects will be randomized to either PSI ≤90 (class I-III) or PSI ≥91 (Class IV-V)

Attachment 2:
Preparation and Administration of Intravenous Infusion Solutions of Ceftobiprole Medocaril (500 mg dose)

General Instructions:

- Ceftobiprole medocaril lyophilizate must only be reconstituted with the reconstitution solution provided by the sponsor.
- Reconstituted ceftobiprole medocaril must not be administered as a bolus, but must be further diluted in 250 mL 5% dextrose (glucose) for i.v. infusion over 120 minutes.
- Do NOT use infusion solutions containing NaCl as this may result in precipitation of the drug.
- Do NOT use glass containers for study drug infusion.

Precautions:

- Protect ceftobiprole medocaril from direct sunlight (lyophilizate as well as solution).
- Do NOT freeze the reconstituted solution.

Personal Protection:

Caution should be exercised when preparing ceftobiprole medocaril for administration.

- The use of plastic gloves and safety glasses is recommended to avoid exposure in case of breakage or spillage of the vial.
- If the lyophilized or diluted ceftobiprole medocaril contacts skin or mucus membranes, immediately rinse thoroughly with soap and water; rinse eyes thoroughly with plain water.

Preparation of the Infusion Solution:

The infusion solution must be prepared and administered according to GCP standards.

1. For each infusion use 1 vial of 500 mg ceftobiprole medocaril lyophilizate and the corresponding reconstitution solution.
2. Inject 10 mL reconstitution solution into the vial containing 500 mg ceftobiprole medocaril.
3. Enter the exact time of start of dissolution into the appropriate preparation record.
4. Shake each vial well until complete dissolution of the drug. A yellowish solution is obtained.
5. Visually inspect the reconstituted solution for particulate matter. If any particulate matter is observed, discard the vial.
6. The resulting reconstituted solution in each vial allows the withdrawal of 10 mL, equivalent to 500 mg ceftobiprole per vial. The 5 mL withdrawal of reconstituted solution from this vial is equivalent to 250 mg ceftobiprole.

Attachment 2 (Continued):
Preparation and Administration of Intravenous Infusion Solutions of Ceftobiprole Medocaril (500 mg dose)

1. Immediately (within 1 hour after reconstitution) add 10 mL of the reconstituted ceftobiprole medocaril to a 250 mL 5% dextrose infusion bag.
2. Agitate the bag carefully to yield a homogeneous infusion solution.
3. Visually inspect the infusion solution for particulate matter. If any particulate matter is observed, discard the bag.
4. The infusion solution containing the equivalent of 500 mg of ceftobiprole is now ready for infusion.
5. The infusion solution may be stored:
    - at room temperature (25°C) and used within 6 hours, or
    - stored for up to 16 hours under refrigeration (5°C) and used within 3 hours after removal from the refrigerator
    - solutions of ceftobiprole medocaril should not be frozen.
6. The entire content of the bag has to be infused to administer 500 mg of ceftobiprole.

Preparation and administration of intravenous infusion solutions of ceftobiprole medocaril (250 mg dose) for severe renal impairment subjects:

1. Immediately (within 1 hour after reconstitution) withdraw only 5 mL of ceftobiprole medocaril reconstituted solution from the vials and add to a 250 mL 5% dextrose infusion bag.
2. Agitate the bag carefully to yield a homogeneous infusion solution.
3. Visually inspect the infusion solution for particulate matter. If any particulate matter is observed, discard the bag.
4. The infusion solution containing the equivalent of 250 mg of ceftobiprole is now ready for infusion.
5. The infusion solution may be stored:
    - at room temperature (25°C) and used within 6 hours, or
    - stored for up to 16 hours under refrigeration (5°C) and used within 3 hours after removal from the refrigerator
    - solutions of ceftobiprole medocaril should not be frozen.

The entire content of the bag has to be infused to administer 250 mg of ceftobiprole.

Administration:

The infusion solutions will be labeled with e.g., the protocol number, CRF number, and the date of preparation of the infusion solution.

Attachment 2 (Continued):
Preparation and Administration of Intravenous Infusion Solutions of Ceftobiprole Medocaril (500 mg dose)

Infusion:

Administer the properly diluted ceftobiprole medocaril infusion solution over 120 minutes using a constant-rate infusion pump, if possible.

1. Ensure that the infusion pump has been correctly calibrated before first use.
2. Use tubing with a low retention volume to connect the infusion pump to the venous catheter.
3. Prime the tubing with the drug solution before starting the infusion.

Administer the medication through a catheter into a vein with adequate venous flow, in order to reduce the risk of phlebitis.

Attachment 3*:

Procedure for Collection, Culture, Susceptibility Testing, Storage, and Shipment of Microbiological Samples

Routine processing	Study specific tasks	Investigator communication
Reception	Sputum, respiratory material, or blood cultures should arrive labeled as "study sample".<br>Transfer to local laboratory within 2 hours of collection.<br>If not possible, samples must be stored at 2°C to 8°C and sent refrigerated to local laboratory within 24 hours.<br>Blood cultures must be stored at 37°C.	--
Microscopy	Perform Gram stain on all respiratory specimens per local laboratory procedures<br>Sputum: WBC ≥25 per low power field (LPF); squamous epithelial cells <10 per LPF classifies as valid sample.<br>*Stick study label on all slides*	If sample is not valid, ask investigator to consider taking a new sample
Initiate culture	Initiate culture as usual per local laboratory procedures.<br>perform semi-quantitative plating for sputum specimens and quantitative culture on BAL washings and protected brushings, include culture medium for *Legionella*<br>*(if available, please add a Staphylococcus selective agar)*<br>*Store "original sample" for 7 days at 4°C*<br>*Study labels to stick on plates and samples are available*	--
Identification	Identify pathogens as usual per local laboratory procedures.	*If Legionella spp are isolated, inform the investigator, as subject must be discontinued If Extended spectrum beta-lactamase (ESBL) producers, Proteus vulgaris or*<br>*- ceftriaxone- or ceftobiprole-resistant nonfermenters isolated. Inform the investigator to consider withdrawing the subject*
Susceptibility testing	Perform susceptibility testing as usual<br>In addition, ceftobiprole E-test® and disks are made available for susceptibility testing (see Section A for specifics and interpretation)<br>*Study labels to stick on plates are available*<br>*File report copy until end of study*	--
Issue report	Issue a laboratory report as usual	Ceftobiprole susceptibility can be reported in e.g., the comment section
Storage and shipment	*Store duplicates of frozen isolates from relevant primary and secondary pathogen (see Section C)*<br>*Complete laboratory requisition form Micro isolates*	Specify primary pathogen<br>(and secondary if necessary) and which strains will be shipped

* This section of the protocol has been revised. Please refer to the section of this document entitled PROTOCOL AMENDMENTS (Amendment INT-3, 22 December 2006) for a detailed description of the specific changes.

Attachment 3 (Continued):
Procedure for Collection, Culture, Susceptibility Testing, Storage, and Shipment of Microbiological Samples

A: Susceptibility Testing of Ceftobiprole (BPR)

Your local monitor will provide ceftobiprole 30 µg disks labeled with BPR and E-test strips

MIC range: BPR Etest strips are either 0.002 to 32 µg/m or 0.016 – 256 µg/mL.

Disks of ceftobiprole (BAL9141) are stable for at least 3 years when stored at -20°C. Disk vials in use can be stored at 2°C to 4°C for 1 week as indicated on label. E-tests should be stored at –20°C. If a blister package is opened, store in airtight container with desiccant at –20°C.

These test materials are validated according to Clinical and Laboratory Standards Institute (CLSI) guidelines. Please refer to the Guidance for Susceptibility Testing for details.

B: Selection of Pathogens to be Stored and Shipped to the Central Laboratory

"BAL and protected brush are preferred sampling techniques; sputa are acceptable e.g., if other techniques are not possible. If sputa are obtained in baseline or postbaseline assessments, the presence or absence of leucocytes and density of epithelia must be recorded in the CRF. Cultures from sputa will only be deemed evaluable in the presence of moderate or abundant leucocytes and low or absent epithelia in the sample".

A sputum sample must contain ≥25 WBC per LPF and <10 per LPF epithelial cells to be valid

Microscopy —Gram stain (WBC ≥25 per LPF, Squamous cells <10 per LPF)	Culture result	Appropriate Action
OK	Pathogens (e.g., *S. aureus, S. pneumoniae*)	Send primary (and secondary) pathogen to central laboratory<br>If more than one pathogen, send the one(s) that match best with microscopy
OK	Commensal (Oral) flora	Do <u>not send</u> any isolate that is<br>- a typical contaminant from oral commensal flora<br>Or<br>- Unlikely to cause pneumonia in other than severely immunocompromised patient (e.g., enterococci, *candida* spp.)
Not OK	Pathogens (e.g., *S. aureus, S. pneumoniae*)	Sample not valid<br>Send only MRSA or PRSP or follow-up isolates from baseline pathogen (for MIC comparison, not efficacy)
Not OK	Commensal (Oral) flora	Sample not valid

Attachment 3 (Continued):
Procedure for Collection, Culture, Susceptibility Testing, Storage, and Shipment of Microbiological Samples

C: Shipment and Storage

Isolates not considered as pathogens in this study, should NOT be shipped to the central laboratory. All potential pathogens should be stored at –70°C in cryovials for shipment to the central laboratory.

(If storage at –70°C is not possible, TSBS tubes provided for the study should be used for storage at –20°C)

Use the provided label-sticker for the respective isolates.

Duplicate strains must be stored at the local laboratory until the study is completed.

D: Requisition Form

Please complete the requisition form for each organism that has been isolated.

Please see separate instructions for storage and shipment to the central laboratory.

E. Guidance for Susceptibility Testing

<u>Technical specifications</u>

E-test Ceftobiprole (former BAL9141)

The 3-letter code BPR will be used to identify ceftobiprole, BAL9141 on E-test strips.

The E-test MIC range is either 0.016 to 256 µg/mL or 0.002 to 32 µg/mL.

Product specifications are:

Article No: 51003538

Pack size: 100 strips

Delivered to local laboratories as single blister packs of 10 strips.

Ceftobiprole Disks for Disk Diffusion

The disks contain 30 µg of ceftobiprole (BAL9141). The 3-letter code BPR will be used to identify ceftobiprole (BAL9141) on disks.

<u>Transportation and shelf-life recommendations for E-test strips and disks</u>

a) Unopened packages to be stored at -20°C will have a shelf-life based on the expiration date of the disks or strips, no longer than 1 year from the date of delivery.

Attachment 3 (Continued):
Procedure for Collection, Culture, Susceptibility Testing, Storage, and Shipment of Microbiological Samples

b) E-test strips from opened packages must be placed in airtight storage tubes or storage containers with desiccant and stored at -20°C.

c) Strips can be transported at room temperature (+22°C to 30°C) for a maximum of 1 week.

d) Disks should be stored frozen at –20°C. A working supply of disks "in use" can be stored at 2°C to 4°C for 1 week.

E-test and disks must be immediately stored at -20°C on arrival. Cold transport is recommended for transporting to hot and humid countries, if delivery, handling and custom clearance are unpredictable.

Procedure

Medium

Mueller Hinton agar or equivalent medium should be used for E-test MIC and disk diffusion testing.

Inoculum

Sixteen to 18-hour colonies should be suspended in 0.85% saline to obtain a turbidity equivalent to 0.5 McFarland turbidity standard. This suspension will be swabbed on the agar plates 3 times, each time rotating the agar plate 60° completely covering the agar surface with inoculum.

E-test: Before placement of E-test strip, allow the inoculum to dry on agar surface for approximately 10 minutes.

DISK: Before placement of disk, allow the inoculum to dry on the agar surface for 3 to 5 minutes.

Placement of E-test Strip

Using forceps, deposit an E-test strip on the inoculated agar plate making sure the strip is in complete contact with the surface. Do not move or remove the strip once it is in place, since the antimicrobial agents is released immediately onto the agar surface.

Placement of Disk

Using forceps or disk dispenser, place a disk onto the surface of the inoculated agar plate. A disk should not be relocated once it comes into contact with the agar surface.

Incubation for both E-test and Disk Diffusion

Plates are inverted and incubated at +35°C under ambient conditions for 16 to 20 hours. If deemed necessary, *Streptococci and Haemophilus* spp. should be investigated using appropriate media and atmosphere.

Attachment 3 (Continued):
Procedure for Collection, Culture, Susceptibility Testing, Storage, and Shipment of Microbiological Samples

Quality control

To validate E-test runs and disk diffusion testing, at least 1 closely related CLSI quality control strain should be tested in parallel with the subject's isolate. If susceptibility testing is set up more than once a day, ceftobiprole QC should be tested at least daily if there are subject isolates to be tested. Quality control strain results should be within the QC ranges listed below.

Interpretation of results

Interpret measured zones of inhibition or Etest result by reference to the following table to classify test isolate as Susceptible (S).

Proposed breakpoint for ceftobiprole (BAL9141)

**Proposed breakpoint for ceftobiprole (BAL9141)**		
Interpretation	MIC testing	Disk Diffusion testing
Susceptible	≤4 µg/mL	≥20 mm

Quality control

The list below illustrates a range of performance control strains in routine use.

Organism	Tentative MIC range (µg/mL)[a]	Tentative disk diffusion range (mm)[a]
S. aureus ATCC 29213	0.25 - 1	--
S. aureus ATCC 25923	--	26-34
E. faecalis ATCC 29212	0.06 - 0.5	--
E. coli ATCC 25922	0.03 - 0.12	30-36
P. aeruginosa ATCC 27853	1-4	--
S. pneumoniae ATCC 49619	0.004 - 0.03	--
H. influenzae ATCC 49247	0.12 - 1	--
H. influenzae ATCC 49766	0.015 - 0.06	30-38

[a] Approved by CLSI in June 2005. Considered to be tentative for 1 year after publication in January 2006.

User quality control: Check for signs of deterioration. Quality control must be performed with at least one organism to demonstrate a correct susceptibility pattern. Do not use the product if the reactions with the control organisms are incorrect.

Limitations

Any deviation from the prescribed method may produce incorrect results.

THE LATEST PUBLISHED VERSION OF THE METHOD USED SHOULD BE CONSULTED FOR COMPLETE DETAILS OF TEST PROCEDURES AND INTERPRETIVE CRITERIA.

LAST PAGE
